# Synergistic cascade catalysis by metal nanoparticles and Lewis acids in hydrogen autotransfer†
†Electronic supplementary information (ESI) available: General procedures, materials, and instrumentation; synthesis, characterization and relevant spectra/charts; procedures and results for optimization and additional experiments. See DOI: 10.1039/c4sc03627a



**DOI:** 10.1039/c4sc03627a

**Published:** 2014-12-17

**Authors:** Gerald C. Y. Choo, Hiroyuki Miyamura, Shū Kobayashi

**Affiliations:** a Department of Chemistry , School of Science , The University of Tokyo , Hongo, Bunkyo-ku , Tokyo 113-0033 , Japan . Email: shu_kobayashi@chem.s.u-tokyo.ac.jp

## Abstract

Of the many types of catalysis involving two or more catalysts, synergistic catalysis is of great interest because novel reactions or reaction pathways may be discovered when there is synergy between the catalysts. Herein, we describe a synergistic cascade catalysis, in which immobilized Au/Pd bimetallic nanoparticles and Lewis acids work in tandem to achieve the *N*-alkylation of primary amides to secondary amides with alcohols *via* hydrogen autotransfer. When Au/Pd nanoparticles were used with metal triflates, a significant rate acceleration was observed, and the desired secondary amides were obtained in excellent yields. The metal triflate is thought to not only facilitate the addition of primary amides to aldehydes generated *in situ*, but also enhance the returning of hydrogen from nanoparticles to hydrogen-accepting intermediates. This resulted in a more rapid turnover of the nanoparticle catalyst, and ultimately translated into an increase in the overall rate of the reaction. The two catalysts in this co-catalytic system work in a synergistic and cascade fashion, resulting in an efficient hydrogen autotransfer process.

## Abstract 2

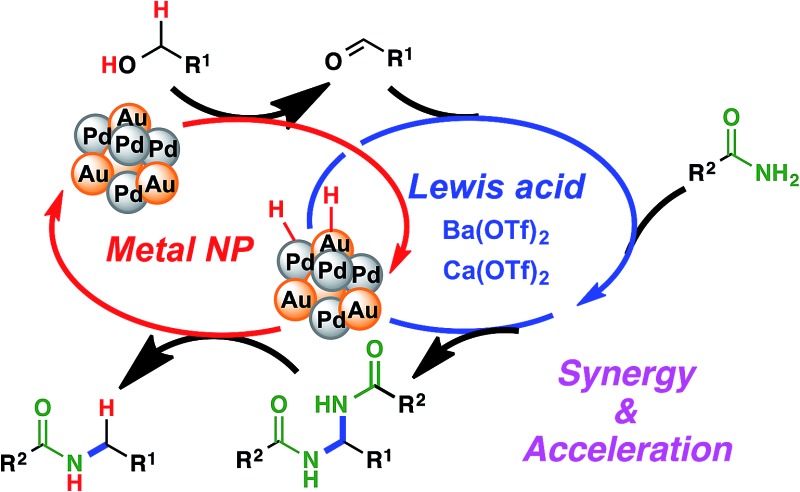
Synergistic cascade catalysis of Au/Pd nanoparticles/Lewis acids achieved *N*-alkylation of primary amides to secondary amides with alcohols *via* hydrogen autotransfer.

## Introduction

Catalysis with metal nanoparticles^1,2^ is a hot research field that has gained much attention. Metal nanoparticles have great potential as catalysts because of their facile heterogenization, robustness, and unique reactivity and selectivity that result from their characteristic electronic state. From the early reports of Au nanoparticle-catalyzed aerobic oxidation reactions^3–14^ to recent reports of bond-forming reactions, chiral^15–19^ or otherwise, metal nanoparticles have been widely investigated as extremely active catalysts, and applied to many reactions.^20–26^ The concept of employing two distinct catalysts in one reaction system is a powerful strategy in organic synthesis to accelerate reactions efficiently in a synergistic manner,^27–29^ but the use of metal nanoparticles in such systems is less well-developed possibly due to catalyst incompatibility; a second catalyst sometimes deactivates metal nanoparticles. If compatibility issues are ironed out,^30^ metal nanoparticles show great potential and possibility for use in synergistic catalysis.^27^

Hydrogen autotransfer, also known as “borrowing hydrogen,” is a useful methodology for the formation of C–C and C–N bonds. The attractiveness of hydrogen autotransfer lies in its high atom economy because no external oxidant is required for the activation of substrates, and no external reductant is required for the reduction of intermediates generated *in situ*. The hydrogen autotransfer methodology has been developed using homogeneous Ir, Rh, Pd and Ru metal complexes as catalysts.^31–38^ However, the recovery and reuse of the precious metals in these reactions are usually difficult. The use of metal with an organocatalyst^39^ for hydrogen autotransfers has been reported recently. Metal nanoparticles have also been demonstrated to be effective catalysts for the hydrogen autotransfer process.^40,41^ The alkylation of amines using alcohols *via* hydrogen autotransfer has been widely reported but reports of the *N*-alkylation of primary amides *via* hydrogen autotransfer are quite limited compared to those of the alkylation of amines despite the potential synthetic utility of the reaction.^38,42–45^ This could be because amides are generally unreactive when compared to amines so the nucleophilic attack of a primary amide to an aldehyde generated *in situ* during the hydrogen autotransfer process is difficult.

**Scheme 1:**
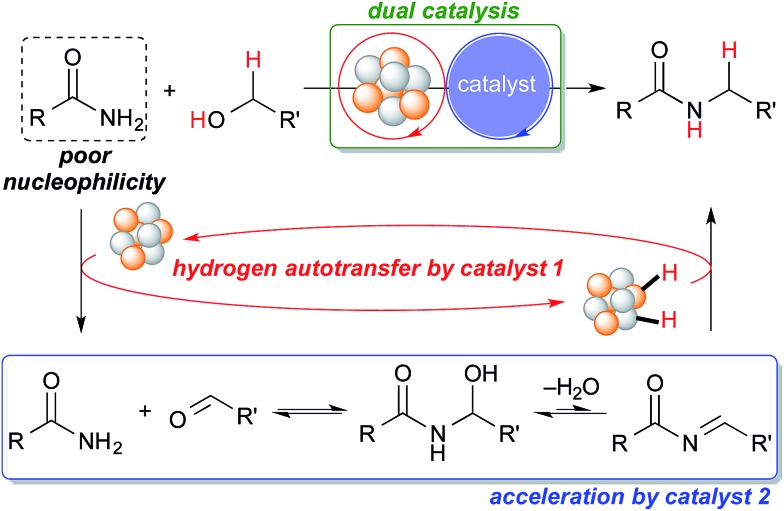
Proposed dual catalysis for the challenging *N*-alkylation of primary amides *via* hydrogen autotransfer.

Our group has been investigating polymer-incarcerated (PI) metal nanoparticles as catalysts for a variety of reactions such as coupling reactions, aerobic oxidation of alcohols to aldehydes/ketones, hydrogenation/reduction reactions and tandem oxidation processes.^46–49^ In many cases, the reaction conditions are mild because the immobilized metal nanoparticles are very active and facilitate the above-mentioned reactions effectively. More recently, we have been interested in employing immobilized metal nanoparticles and other functional molecules in reactions systems, the synergy of which has paved the way for many interesting reactions^50^ and tandem oxidation processes.^30,51^ We were, therefore, interested in the synergistic catalysis between the PI metal nanoparticle catalyst and a second catalyst for the challenging hydrogen autotransfer reaction between primary amides and alcohols. We expected the PI metal nanoparticle catalyst to be an effective catalyst for hydrogen autotransfer because we are able to immobilize various metal nanoparticles, including multi-metallic nanoparticles,^17,30,50–55^ and therefore, we are able to tune catalytic activity easily by choosing appropriate metal sources.^53–55^ The second catalyst is expected to enhance the efficiency of the overall reaction by facilitating the nucleophilic addition of the primary amide to the carbonyl compound generated *in situ*, which is a key but slow step due to the poor nucleophilicity of primary amides (Scheme 1).

## Results and discussion

### Discovery of a suitable immobilized nanoparticle catalyst for hydrogen autotransfer between benzamide and benzyl alcohol

With benzamide (**1a**) and benzyl alcohol (**2a**) as substrates for the model reaction, initial screening of various polymer-incarcerated metal nanoparticle catalysts with carbon black as a secondary support (PI/CB-M catalysts) was conducted. Initially, we followed an earlier report and adopted benzyl alcohol as the solvent.^56^ Under the reaction conditions shown in Table 1, the reaction did not proceed when typical metals for hydrogen autotransfer, such as Ir, Ru and Rh, were used. No product was observed for PI/CB-Ni or PI/CB-Co either (entry 1). We then turned our attention to Au^3–14^ and Pd^24,57–68^ nanoparticle catalysts because these catalysts have been widely investigated and demonstrated to be effective catalysts for aerobic oxidation, dehydrogenative oxidation,^68^ hydrogenation and bond forming reactions. While PI/CB-Au did not afford any product (entry 2), a trace amount of product was detected with PI/CB-Pd (entry 3).

**Table 1:** Effect of oxygen and additive on the reaction

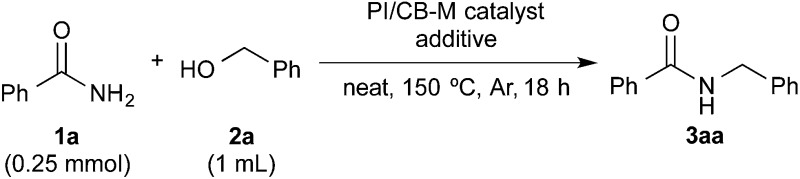			
Entry	M^*a*^	Additive	Yield^*b*^ (%)
1	Ir, Ru, Rh, Ni or Co	—	n.d^*c*^
2	Au	—	n.d^*c*^
3	Pd	—	Trace^*c*^
4^*d*^	Au	—	0
5^*d*^	Pd	—	7
6^*d*^	Au (2 mol%) + Pd (2 mol%)	—	24
7^*d*^	Au/Pd (Au : Pd = 1 : 1)	—	43
8^*d*^	Au/Pd (Au : Pd = 1 : 1)	MgSO_4_ (1.66 eq.)	89
9^*d*^	—	MgSO_4_ (1.66 eq.)	0
10^*d*^	—	—	0

^*a*^Catalyst loading was set to 2 mol%. In the case of bimetallic catalysts, the catalyst loading was set to 2 mol% with respect to the first metal stated.

^*b*^Determined by GC analysis with dodecane as the internal standard.

^*c*^Determined by GCMS analysis of crude after the stipulated reaction time (n.d. = not detected).

^*d*^Deoxidized benzyl alcohol was used.

After many attempts at improving the yield, the desired *N*-benzylbenzamide product (**3aa**) was obtained in 7% yield with PI/CB-Pd, using benzyl alcohol that was carefully degassed (entry 5), which was a marked improvement over the trace amount obtained earlier. PI/CB-Au, however, still did not afford any product (entry 4), interestingly, a physical mixture of PI/CB-Au and PI/CB-Pd catalysts afforded more of the desired product, although the yield, at 24%, was still unsatisfactory (entry 6).

When the PI/CB-Au/Pd bimetallic nanoparticle catalyst was employed, we observed a dramatic increase in yield to 43% (entry 7). The metal nanoparticles in the catalyst was confirmed to be alloyed by scanning transmission electron microscopy (STEM) and energy-dispersive X-ray spectroscopy (EDS) analyses; the catalyst is not a mixture of Au and Pd nanoparticles that are independent of each other. Alloyed bimetallic nanoparticles often demonstrate catalytic properties that are unique from their monometallic counterparts;^69–73^ Au is reported to have a promotional effect on Pd such that when the two are combined, it results in a more active catalyst.^74,75^ We believe that the promotional effect of Au is more pronounced when the metal nanoparticles are bimetallic alloy nanoparticles, due to the proximity of Au to Pd. In addition, the better catalytic activity may also be attributed to the polarization of electric charge on the surface of the alloyed bimetallic nanoparticle arising from the difference in electronegativity between Au and Pd.^69,71^

In spite of all our subsequent attempts, it was difficult to improve the yield beyond 43%. We then decided to examine the postulated mechanism of the reaction, and focused our attention on water that was formed as a byproduct (bottom of Scheme 1). We hypothesized that removing water from the reaction system would favor the formation of the acylimine intermediate and improve the yield. Pleasingly, when MgSO_4_ (50 mg, 1.66 eq.) was employed as an additive, the yield improved significantly to 89% (Table 1, entry 8). A control experiment in which only MgSO_4_ was employed without the catalyst confirmed that MgSO_4_ was not the main catalyst because no product **3aa** was observed (entry 9).

### Lewis acid effect outweighs desiccant effect

Based on these initial results, we proceeded to reduce the amount of benzyl alcohol required from solvent amount to 4 equivalents (see ESI 4-2†). We found that toluene, in place of benzyl alcohol (**2a**) as the solvent, was effective for the reaction. After optimization of the reaction conditions using toluene as the solvent, we obtained the desired amide quantitatively with 4 equivalents of benzyl alcohol (Table 2, entry 1). It should be noted that the amide alkylation reaction proceeded under neutral conditions. Decreasing the amount of MgSO_4_ from excess to catalytic resulted in a decrease in yield (see ESI† 4-2†). Despite this result, we were, at this juncture, unable to rule out MgSO_4_ working as a Lewis acid for the addition of benzamide to benzaldehyde. Several other additives were screened to determine their effect on the reaction and also to determine if there was a more effective additive that could be used in catalytic amounts (Table 2, entries 2–17).

**Table 2:** Screening of additives and equivalents of benzyl alcohol

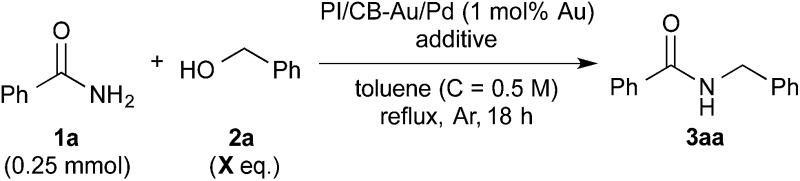				
Entry	Additive	Amount	*X* = 4^*a*^	*X* = 3^*a*^
1	MgSO_4_	1.66 eq.	Quant.	91
2	MgZ_2_ (Z = Fl, Cl, Br)	1.66 eq.	1–17	—
3	MgO	1.66 eq.	31	—
4	Mg(OH)_2_	1.66 eq.	31	—
5	Mg(OTf)_2_	1.66 eq.	97	—
6	Mg(OTf)_2_	0.5 eq.	Quant.	—
7	Mg(OTf)_2_	5 mol%	Quant.	Quant. (64)^*c*^
8	Ca(OTf)_2_	5 mol%	99	Quant. (63)^*c*^
**9**	**Ba(OTf)**_**2**_	**5 mol%**	**98**	**Quant. (95)** ^*b*^ **(85)** ^*c*^ **(94)** ^*d*^
10	LiOTf	5 mol%	Quant.	90
11	NaOTf	5 mol%	98	—
12	KOTf	5 mol%	73	—
13	Sc(OTf)_3_	5 mol%	98	92
14	Yb(OTf)_3_	5 mol%	Quant.	Quant.
15	TfOH	5 mol%	70	—
16^*e*^	MS 3 Å or MS 4 Å	20 mg	<10	—
17^*e*^	MS 5 Å	20 mg	87	—
18	—	—	50	—

^*a*^Yield was determined by GC analysis with dodecane as the internal standard.

^*b*^2.5 eq. of benzyl alcohol were used.

^*c*^GC yield obtained when the reaction was conducted at 120 °C (hot plate temperature).

^*d*^4-Methylbenzyl alcohol used as the substrate; a 5 : 1 ratio of toluene : H_2_O was used as the solvent.

^*e*^Catalyst loading: 2 mol% Au; solvent: xylene (*C* = 0.25 M).

Initially, Mg salts were examined. Neither Mg halides nor MgO nor Mg(OH)_2_ worked well as additives for the reaction (entries 2–4). However, the reaction proceeded smoothly to afford the desired product almost quantitatively with Mg(OTf)_2_ (entry 5). Encouraged by this result, we reduced the amount of Mg(OTf)_2_ in subsequent experiments to determine if Mg was acting as a Lewis acid for the reaction. Satisfyingly, Mg(OTf)_2_ worked well as an additive even at 0.5 equivalents and 5 mol% (entries 6 and 7). That Mg(OTf)_2_ could be employed catalytically as a co-catalyst while MgSO_4_ could not was probably due to a difference in Lewis acidity arising from the difference in counteranions.^76^ Other Group 2 metal triflates were also screened and they were found to be effective co-catalysts for the reaction as well (entries 8 and 9). We then examined some metal triflates from the neighboring groups and most of them worked well (entries 10–14); >95% of the desired product was obtained, with the exception of KOTf.

When water was deliberately introduced into the reaction vessel, the reaction still proceeded to give the desired product in good yield (entry 9 with footnote *d*, see ESI 4-5†). Furthermore, when molecular sieves were used as the additive (entries 16 and 17), only molecular sieves 5 Å gave good yield (entry 17), suggesting that rather than the dehydrating properties of the additive, it was the acidity of the additive that was crucial for the reaction. Thus, the results from these control experiments ruled out MgSO_4_ working as a desiccant.

When triflic acid was examined as the co-catalyst, the yield was 70% (entry 15). This yield, which was higher than when no co-catalyst was employed (entry 18), demonstrated that acidity was important for the reaction but it also suggested that Lewis acidity is more crucial than Brønsted acidity because the yield was still lower than when a Lewis acid such as Mg(OTf)_2_ was employed.

We then further optimized the reaction by employing the effective Lewis acid co-catalysts to the model reaction with 3 equivalents of benzyl alcohol (*X* = 3 column in Table 2). Group 2 metal triflates worked extremely well for the reaction, affording the desired product quantitatively (Table 2, entries 7–9). On the other hand, LiOTf (entry 10) and Sc(OTf)_3_ (entry 13) did not perform as well. Yb(OTf)_3_ also gave the desired product quantitatively (entry 14), albeit with the formation of several side products. Under a lower temperature of 120 °C (heating plate), Ba(OTf)_2_ outperformed the other Group 2 metal triflates screened (entries 7–9). In addition, because excellent yield (95%) was also achieved with 2.5 equivalents of benzyl alcohol with Ba(OTf)_2_ (entry 9 with footnote *b*), we decided to adopt it as the co-catalyst for our reaction system.

### Substrate scope: Excellent yields achieved for difficult aliphatic amide substrates

With the optimized reaction conditions in hand, we proceeded to examine various substrates for the reaction (Table 3). In general, when benzyl alcohol (**2a**) was used, benzamide (**1a**) and its analogs worked well to afford the products in excellent yields, especially those with electron-donating substituents on the aromatic ring (entries 1–4). This can be attributed to the increased nucleophilicity of the amide. Even with a *p*-fluoro-substitution, the reaction proceeded well to afford the desired product in high yield (entry 6). Heteroaromatic benzamide analogs were also applicable to this reaction system, albeit with modified reaction conditions to improve the yields (entries 7 and 8). We suspect that the heteroatom on the aromatic ring could have coordinated to the Au/Pd bimetallic nanoparticles or the Lewis acid, resulting in a slight deactivation of the desired catalysis. We then turned our attention to aliphatic substrates (compound **1**, R^1^ = alkyl), which are difficult substrates in hydrogen autotransfer. Satisfyingly, all aliphatic substrates afforded the desired products in more than 90% yield (entries 10–14), with the exception of acetamide, for which more benzyl alcohol (**2a**) was required to obtain a good yield of 77% (entry 9). This is a marked improvement over earlier reports that used aliphatic substrates (**1i–1n**) with benzyl alcohol in hydrogen autotransfer^38,44^ and this highlights one of the advantages of our synergistic catalytic system.

**Table 3:** Substrate scope

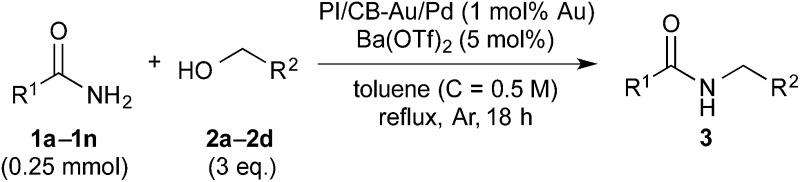				
Entry		R	**3**	Yield^*a*^ (%)
1	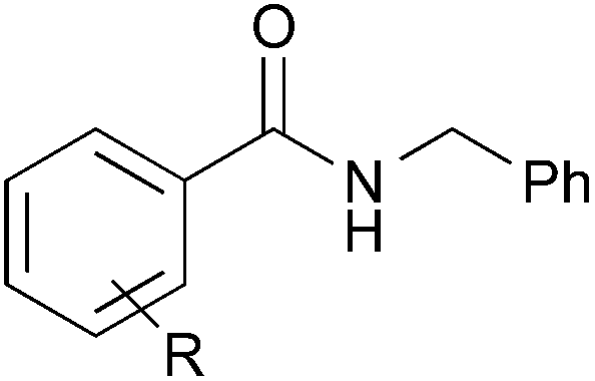	H	**3aa**	95
2		*p*-Me	**3ba**	98
3		*p*-MeO	**3ca**	Quant.
4		*o*-EtO	**3da**	89
5		*o*-OH	**3ea**	53
6		*p*-F	**3fa**	90

7^*b*^	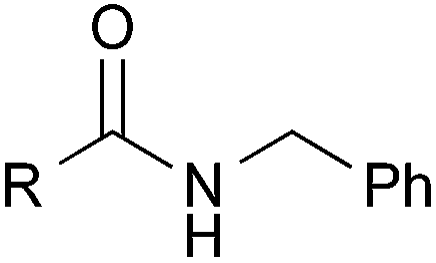	2-Pyridyl	**3ga**	63
8^*c*^		3-Pyridyl	**3ha**	52
9^*b*^		Me	**3ia**	77
10		*n*-C_5_H_11_	**3ja**	95
11		i-Pr	**3ka**	94
12		*t*-Bu	**3la**	90
13		Bn	**3ma**	91
14		*c*-Hex	**3na**	Quant.

15	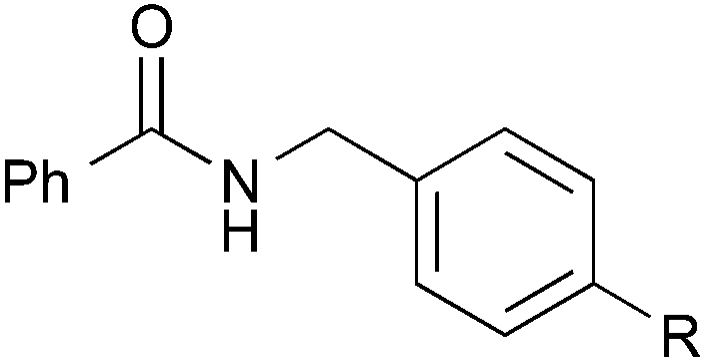	Me	**3ab**	Quant.
16^*d*^		CO_2_Me	**3ad**	61
17	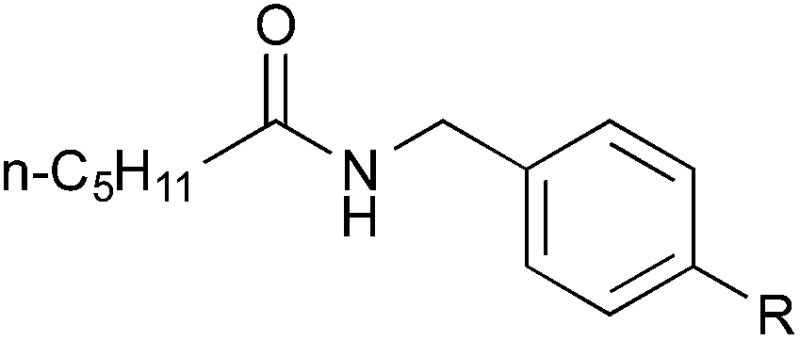	Me	**3jb**	86
18		MeO	**3jc**	44
19		CO_2_Me	**3jd**	68

^*a*^Isolated yield.

^*b*^5 eq. of **2a** were used.

^*c*^2 mol% Au and 10 mol% Ba(OTf)_2_ were used.

^*d*^With some impurity (alcohol starting material); refer to ESI.

Other benzyl alcohol analogs were then employed in the reaction (entries 15–19). For *p*-methyl-substituted benzyl alcohol, high yields were obtained for both benzamide and hexanamide (entries 15 & 17). When an even more electron-rich benzyl alcohol analog was employed, the yields were moderate (entry 18), possibly due to the reduced electrophilicity of the carbon on the carbonyl moiety of the corresponding aldehyde generated *in situ*. Conversely, when an electron-poor benzyl alcohol analog was employed, the yields were slightly higher (entries 16 & 19). Attempts at using aliphatic alcohols have proven futile and examination of the reaction mixture indicated to us that the problem was the conversion of the alcohol (*vide infra*).

### Reuse of heterogeneous immobilized gold–palladium nanoparticle catalyst

We then proceeded to examine the reusability of our PI/CB-Au/Pd catalyst in our reaction system (Table 4). With the addition of Ba(OTf)_2_ for each run (Table 4, upper row), the heterogeneous catalyst could be reused in run 2 with no pre-treatment required but in run 3, a significant decrease in yield was observed. We believed that the Au/Pd bimetallic nanoparticles might have been deactivated. Taking cues from our previous work,^10,17,53–55^ the recovered catalyst from run 3 was reactivated by heating it at 170 °C for 5 hours under open air before it was used in run 4. Remarkably, catalytic activity recovered and excellent yields were obtained in subsequent runs; the recovered catalyst had to be treated by the method mentioned above only when the conversion of benzyl alcohol showed signs of slowing down (after runs 6 and 9). High yields of >95% were achieved for all runs thereafter up to run 11 and we confirmed no leaching of both Au and Pd in each and every run, demonstrating the robustness of the heterogeneous PI/CB-Au/Pd nanoparticle catalyst for the hydrogen autotransfer process.

**Table 4:** Reusing of the heterogeneous catalyst – PI/CB-Au/Pd

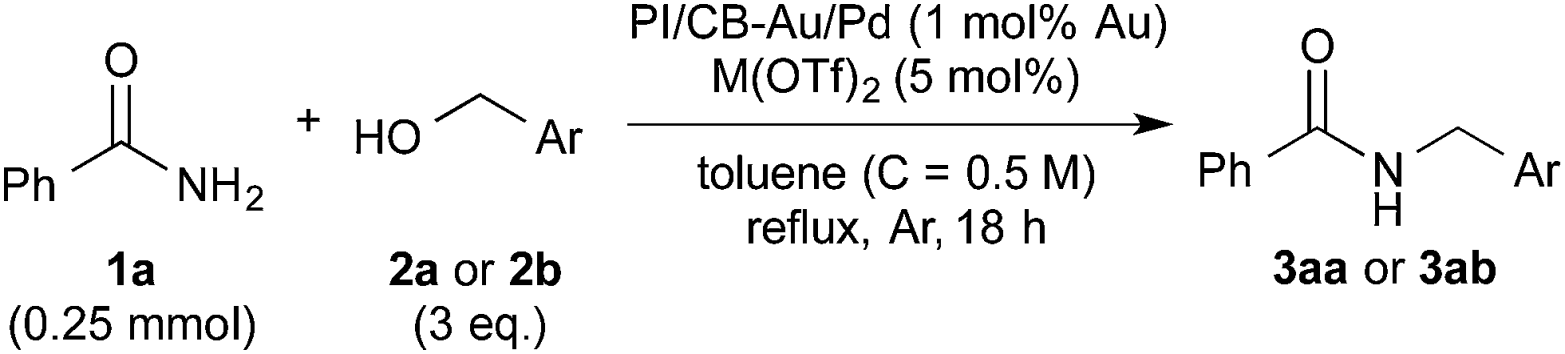						
**2a**; M = Ba	Run	1^*b*^	2^*b*^	3^*b*^	4–11^*b*^ ^,^ ^*c*^	
	Yield^*a*^ (%)	>99	99	53	95 to >99	
**2b**; M = Ca	Run	1	2^*d*^	3^*d*^	4^*c*^ ^,^ ^*d*^	5^*d*^
	Yield^*a*^ (%)	93	95	93	89	93

^*a*^Determined by GC analysis with dodecane as the internal standard.

^*b*^No leaching of Au or Pd was detected (under detection limit; determined by ICP analysis).

^*c*^The recovered catalyst from the previous run was reactivated before use in runs 4, 7 and 10.

^*d*^Recovered catalyst was treated with DCM and no additional Lewis acid was added for the new run.

Reusability of the heterogeneous catalyst is not restricted to the reaction conditions where Ba(OTf)_2_ was used in combination with benzyl alcohol (**2a**). Gratifyingly, when Ca(OTf)_2_ was used as the co-catalyst and 4-methylbenzyl alcohol (**2b**) was used as the substrate, the heterogeneous catalyst could be reused up to 5 times (Table 4, lower row). We also discovered that if the reaction work-up and the recovery of the catalyst was performed using dichloromethane, no additional Ca(OTf)_2_ was required for each run, indicating that the Lewis acid was also recovered in the process (footnote *d* in Table 4 and ESI 3-3†).

### Capturing reaction intermediates and demonstrating *N*-alkylation of amides involving aliphatic substrates

**Scheme 2:**
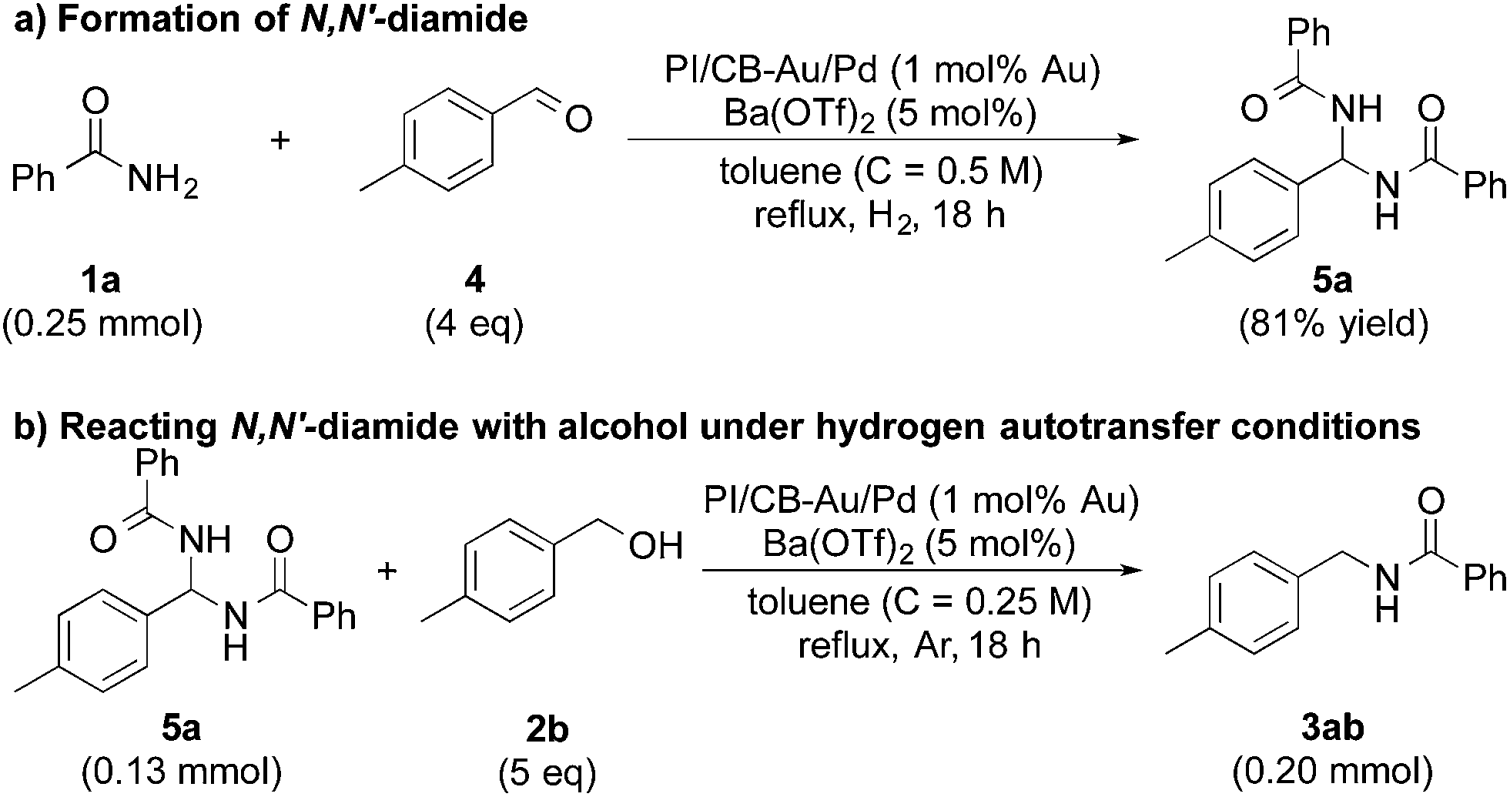
*N*,*N′*-Diamide as a key intermediate of the reaction.

In our quest to gain some insights into the reaction mechanism, we conducted an experiment starting from tolualdehyde (**4**) and benzamide (**1a**) under hydrogen atmosphere. We obtained neither alcohol **2b** nor desired product **3ab**, which suggested that even if hydrogen gas was generated during the reaction (from the hydrogen accepted by the nanoparticle catalyst), hydrogen gas cannot serve as a reductant, and that any reduction that occurs in our reaction system was due to transfer hydrogenation (see ESI 4-6 and 4-14†). Instead, we isolated a solid, in large amounts, that was highly insoluble in many solvents, and we identified it to be *N*,*N*′-(*p*-methylphenylmethylene)dibenzamide (*N*,*N*′-diamide **5a**), formed from one molecule of tolualdehyde (**4**) and two molecules of benzamide (**1a**) (Scheme 2a).^77^ Interestingly, this unexpected compound was also formed under argon atmosphere, regardless of whether Lewis acid was added or not (see ESI 4-7, 4-8 and 4-9†). When **5a** was subjected to the optimized conditions with 4-methylbenzyl alcohol (**2b**), the desired product (**3ab**) was obtained (Scheme 2b). This strongly implied that *N*,*N*′-diamide **5a** could be a key intermediate in the reaction.

**Scheme 3:**
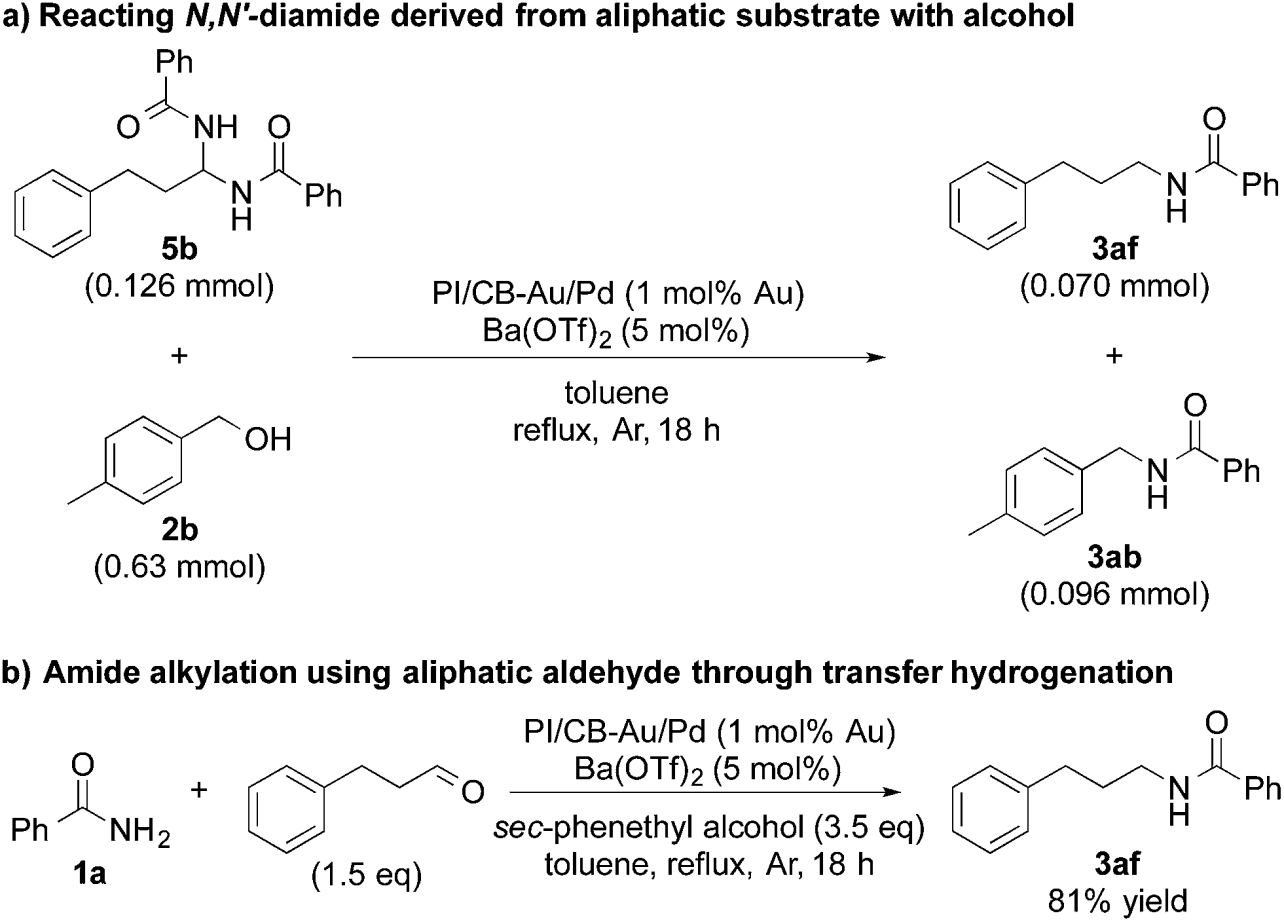
Amide alkylation using aliphatic substrates.

In addition, when we synthesized *N*,*N*′-diamide (**5b**) using 3-phenylpropionaldehyde (aliphatic aldehyde) and benzamide (**1a**), and subjected the newly formed *N*,*N*′-diamide (**5b**) to the optimized conditions with 4-methylbenzyl alcohol (**2b**), we obtained two secondary amides – *N*-(4-methylbenzyl)benzamide (**3ab**) and *N*-(3-phenylpropyl)benzamide (**3af**) (Scheme 3a). Interestingly, when we started out with benzamide (**1a**) and an aliphatic aldehyde, and used a secondary alcohol as the reductant, benzamide (**1a**) was *N*-alkylated smoothly and the desired secondary amide (**3af**) was isolated in 81% yield under our dual catalysis conditions (Scheme 3b). This result demonstrated that our catalytic system is also effective for the *N*-alkylation of amides *via* transfer hydrogenation, when both benzylic and aliphatic aldehydes are used. Furthermore, this reinforces the notion that aliphatic alcohols do not work for our reaction system not because the addition of an amide to an aldehyde is problematic, but because there is difficulty in the initial conversion of the aliphatic alcohol to the corresponding aldehyde.

### Evidence of synergy between metal nanoparticle and Lewis acid

**Fig. 1:**
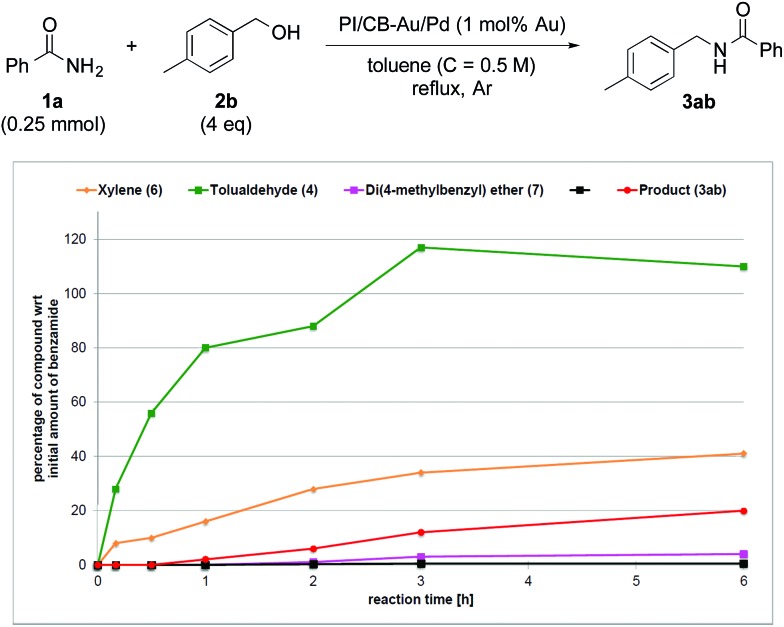
Reaction profile with 4-methylbenzyl alcohol (**2b**) as substrate and no Lewis acid as co-catalyst.

**Fig. 2:**
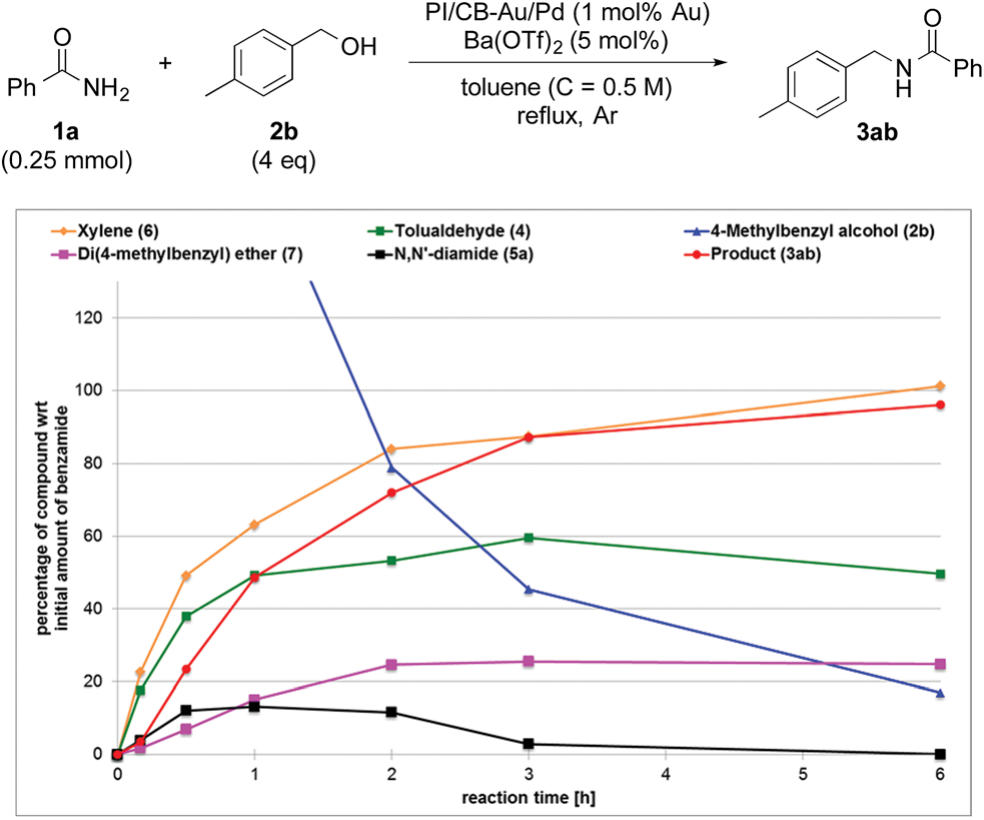
Reaction profile with 4-methylbenzyl alcohol (**2b**) as substrate and with Ba(OTf)_2_ as the co-catalyst.

**Scheme 4:**
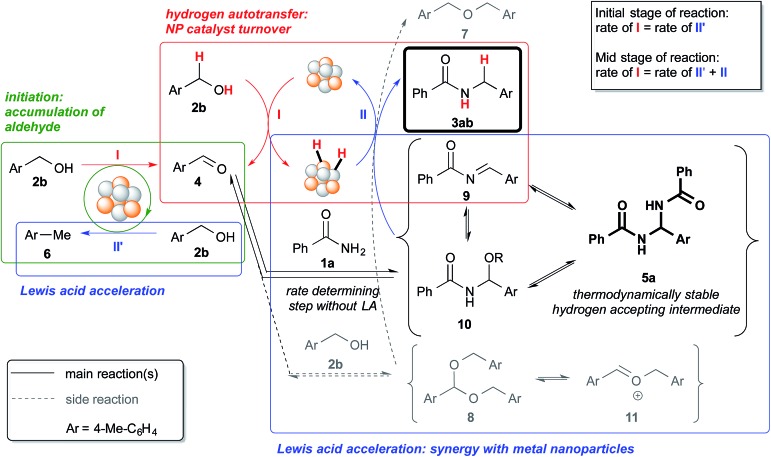
Schematic representation of the processes within the reaction system, which includes an initiation process and Lewis acid acceleration.

To clarify the synergistic effect between metal nanoparticles and a Lewis acid, we monitored the hydrogen autotransfer reactions under various reaction conditions. The reaction profile when 4 equivalents of 4-methylbenzyl alcohol (**2b**) were employed was examined. We monitored the formation/consumption of **2b**, tolualdehyde (**4**), the *N*,*N*′-diamide (**5a**) and the desired product (**3ab**). The resulting reaction profiles obtained without Ba(OTf)_2_ (Fig. 1) and with Ba(OTf)_2_ (Fig. 2) were then compared. During our monitoring, we also observed the formation of two side products – xylene (**6**) and di(4-methylbenzyl) ether (**7**). From control experiments (see ESI 4-12, 4-13 and 4-15†), it was clear that tolualdehyde (**4**) and these two side products were formed from 4-methylbenzyl alcohol (**2b**), only when PI/CB-Au/Pd was present, due to a possible disproportionation-like reaction (steps I and II′ in Scheme 4, ESI 4-4†).^78^ No reaction was observed when an attempt was made to reduce the ether (**7**) using either 4-methylbenzyl alcohol (**2b**) as the hydrogen source, or using hydrogen gas with the PI/CB-Au/Pd catalyst (see ESI 4-16 and 4-17†).

**Fig. 3:**
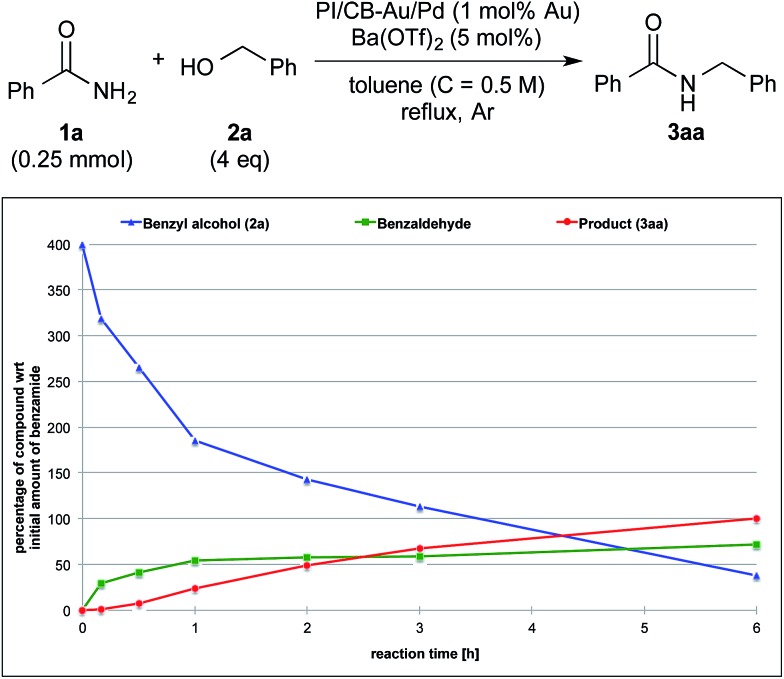
Reaction profile with benzyl alcohol (**2a**) as the substrate and Ba(OTf)_2_ as the co-catalyst.

**Fig. 4:**
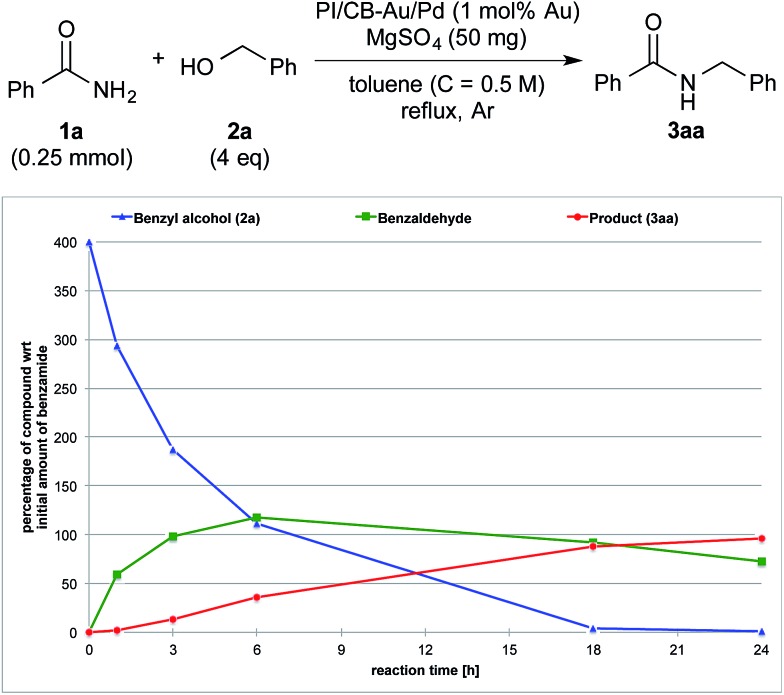
Reaction profile with benzyl alcohol (**2a**) as the substrate and MgSO_4_ as the co-catalyst.

When Ba(OTf)_2_ was absent, a large amount of tolualdehyde (**4**) (approx. 120%, based on the amount of benzamide) was formed after 3 h while the formation of the desired product (**3ab**) was slow, reaching only a mere 20% after 6 h. In addition, xylene (**6**) was steadily formed, reaching 40% after 6 h, and almost no *N*,*N*′-diamide (**5a**) was observed (Fig. 1).

In contrast, when Ba(OTf)_2_ was present (Fig. 2), the rate of formation of the desired product (**3ab**), xylene (**6**) and the ether (**7**) was accelerated. In particular, for the same duration of 6 h, the amount of the desired product (**3ab**) and ether (**7**) formed was almost 4–5 times as much. As a result of this Lewis acid acceleration, the consumption of 4-methylbenzyl alcohol (**2b**) was also much faster. After 30 min into the reaction, we observed the formation of the *N*,*N*′-diamide (**5a**), the concentration of which remained steady until 3 h into the reaction, and then returned to almost zero thereafter. Concurrently, there was a swift increase in the yield of the desired product (**3ab**) during the same period. The amount of tolualdehyde (**4**) increased during this period and reached a steady-state concentration of 50%. This implied that an induction period existed, and that a certain amount of the aldehyde had to be first accumulated before the desired product (**3ab**) started to form. A similar phenomenon was observed even when a Lewis acid was not present (Fig. 1).

We also made a comparison between reaction profiles obtained with 5 mol% of Ba(OTf)_2_ (Fig. 3) and 1.66 equivalents of MgSO_4_ (Fig. 4), with the focus on the alcohol, the aldehyde and the desired product. We observed a dramatic rate acceleration with Ba(OTf)_2_ than with MgSO_4_ (see ESI 4-23†) because the reaction was almost complete after 6 h and the formation of the desired product began much earlier. In addition, we observed a lower concentration of the aldehyde at the steady-state for Ba(OTf)_2_, which implied that the initiation of the reaction was faster and that the induction period was shorter. The results demonstrate that the choice of Lewis acid is important.

### Proposed reaction mechanism

Based on the observations made from the reaction profiles and various control experiments, we propose the reaction mechanism shown in Scheme 4. The reaction begins with the accumulation of the aldehyde (“initiation” process within green box, step I) through a disproportionation-like reaction, which results in the formation of toluene or xylene, and the sacrificial consumption of an alcohol (step II′). The aldehyde then reacts with the primary amide or the alcohol to form hydrogen-acceptors – *N*,*N*′-diamide (**5a**) or acetal (**8**), which equilibrate with the respective acylimine (**9**)/hemiaminal (**10**)/*N*,*O*-acetal (**10′**) or oxocarbenium ion (**11**). Concurrently, hydrogen is being abstracted from the alcohol by the nanoparticle catalyst (step I), and that hydrogen is then “returned” to the hydrogen-acceptors generated in the system to afford the desired product or the ether (step II). The *N*,*N*′-diamide (**5a**) is the most thermodynamically stable compound among the potential hydrogen accepting intermediates (**5a**, **9** and **10** in Scheme 4) because only the *N*,*N*′-diamide was observed in the control experiments starting from aldehydes and primary amides (see ESI 4-6† and Scheme 2a).

The Au/Pd nanoparticle catalyst plays the crucial role of transferring hydrogen from the alcohol to the various hydrogen-acceptors. The Lewis acid, on the other hand, must be involved in both the formation of the *N*,*N*′-diamide^79^ and the hydrogen “returning” process after the said formation. We postulate this based on our observations of the different steady state concentrations of the *N*,*N*′-diamide and the different rates at which the desired product was formed, for experiments with and without the Lewis acid (*vide infra*).

Without a Lewis acid in the system, we expect the formation of the *N*,*N*′-diamide to be the rate-determining step because the *N*,*N*′-diamide is very quickly consumed after it is produced, resulting in the close to zero concentration observed (Fig. 1). We inferred this from the fact that the *N*,*N*′-diamide was formed in high concentration under thermodynamic control even without a Lewis acid when the aldehyde and benzamide was heated under reflux with toluene (see ESI 4-9†). Therefore, that we did not observe any *N*,*N*′-diamide when no Lewis acid was present in our reaction system must imply that there exists a very fast step after the formation of the *N*,*N*′-diamide that leads to the desired product. When a Lewis acid was present, however, the *N*,*N*′-diamide is at steady-state, indicating that the formation of the *N*,*N*′-diamide is no longer the rate-determining step (Fig. 2). The overall rate of the sequential reaction is then governed by the turnover rate of the nanoparticle catalyst, in particular, by the rate of hydrogen “returning”.^80^

The catalytic cycle of the Au/Pd nanoparticle involves two steps – hydrogen abstraction (step I) and returning (steps II′ & II), which are interdependent processes. While there is competition between the desired reaction pathway and the side reaction pathways with regard to accepting hydrogen from the Au/Pd-H_2_ catalyst, the presence of a Lewis acid would lead to the production of various highly reactive hydrogen acceptors (**5a**, **9**, and **10**),^81^ which would result in a faster turnover (step II) for the catalyst from Au/Pd-H_2_ (resting state) to Au/Pd nanoparticle. In turn, that would lead to the production of more aldehyde and thus more hydrogen-acceptor intermediates. As a result of the acceleration of various steps within the reaction system, and also the faster catalytic turnover of the Au/Pd nanoparticle catalyst, the consumption of 4-methylbenzyl alcohol is significantly quickened. However, in the absence of an amide, even with a Lewis acid, consumption of the alcohol was not full even after 18 h (see ESI 4-13 *vs.* 4-24†). This implies that hydrogen abstraction itself is not accelerated by the Lewis acid. Furthermore, the concentration of the aldehyde at steady-state is lower with a more efficient Lewis acid because that lower concentration is presumably sufficient for the hydrogen acceptors to form (Fig. 1 *vs.* Fig. 2 and Fig. 3 *vs.* Fig. 4).

## Conclusion

We have discovered a synergistic cascade catalytic system that employs immobilized Au/Pd nanoparticles and Ca(OTf)_2_/Ba(OTf)_2_ Lewis acid for the *N*-alkylation of primary amides with benzyl alcohol and its analogs *via* hydrogen autotransfer. The choice of metal(s) for the nanoparticle catalyst and choice of Lewis acid is the key to achieve an efficient system. In particular, the performance of the Lewis acid is crucial for overall efficient catalytic turnover. This is a very unique catalytic system in which metal nanoparticles and Lewis acid work synergistically within a complex and elaborated catalytic cycle. This is also the first example of a metal nanoparticle-catalyzed hydrogen autotransfer process that employs primary amides as substrate. The substrate scope was broad and in particular, excellent yields were observed for many difficult aliphatic primary amide substrates. Both metal nanoparticle and Lewis acid were reusable and no leaching of Au and Pd to the product was observed. We strongly believe that such a synergistic system paves the way for us to achieve reactions that with only heterogeneous catalysts, including metal nanoparticles, are currently either impossible or inefficient.
