# Supplementary material for: Synergistic cascade catalysis by metal nanoparticles and Lewis acids in hydrogen autotransfer
Source: Chem Sci. 2014 Dec 17;6(3):1719–27. doi: 10.1039/c4sc03627a (PMC5639791; doi:10.1039/c4sc03627a)
Supplement: Supplementary file 1 [file SC-006-C4SC03627A-s001.pdf]

Electronic Supplementary Material (ESI) for Chemical Science.
This journal is © The Royal Society of Chemistry 2014

Synergistic cascade catalysis by metal nanoparticles and Lewis acids in hydrogen autotransfer

Gerald C. Y. Choo, Hiroyuki Miyamura, Shū Kobayashi*

Department of Chemistry, School of Science, The University of Tokyo
Hongo, Bunkyo-ku, Tokyo 113-0033 (Japan)

Electronic Supplementary Information

1 General Remarks 3

2 Preparation of PI/CB-Au/Pd 4

2-1 Preparation of co-polymer used in the fabrication of PI/CB-Au/Pd 4

2-2 PI/CB-Au/Pd 4

3 Procedure for the *N*-alkylation of primary amides and characterisation of products 8

3-1 General procedure for the *N*-benzylation of benzamide (GC analysis) 8

3-2 N-Alkylation of primary amides and characterisation of products 8

3-3 Recovery and reuse of catalyst & leaching test 11

4 Additional data 13

4-1 Screening of PI/CB catalysts 13

4-2 Optimisation with $MgSO_4$ 13

4-3 Screening of additives 14

4-4 Possible disproportionation-like reaction during the initiation stages of the reaction 14

4-5 Control experiment (1) – Reaction with water 14

4-6 Control experiment (2) – Reductive amidation (Formation of *N*,*N*'-(*p*-methylphenylmethylene)dibenzamide (*N*,*N*'-diamide)) 15

4-7 Control experiment (3) –Formation of *N*,*N*'-(*p*-methylphenylmethylene)dibenzamide (*N*,*N*'-diamide) 15

4-8 Control experiment (4) –Formation of *N*,*N*'-(*p*-methylphenylmethylene)dibenzamide (*N*,*N*'-diamide) 16

4-9 Control experiment (5) –Formation of *N*,*N*'-(*p*-methylphenylmethylene)dibenzamide (*N*,*N*'-diamide) 16

4-10 Control experiment (6) – Transfer hydrogenation from the *N*,*N*'-diamide 16

4-11 Control experiment (7) – Transfer hydrogenation from *N*,*N*'-diamide derived from aliphatic aldehyde 17

4-12 Control experiment (8) - Disproportionation reaction 17

4-13 Control experiment (9) – Disproportionation reaction with $Ba(OTf)_2$ 18

4-14 Control experiment (10) – Hydrogenation of aldehyde 18

4-15 Control experiment (11) – Hydrogenation of alcohol 18

4-16 Control experiment (12) – Hydrogenation of ether 18

4-17 Control experiment (13) – Transfer hydrogenation of ether 19

4-18 Procedure for profiling of reaction 19

4-19 Reaction profile without $Ba(OTf)_2$ 20

4-20 Reaction profile with $Ba(OTf)_2$ 21

4-21 Reaction profile when $MgSO_4$ was employed with benzamide and benzyl alcohol 22

4-22 Reaction profile when $Ba(OTf)_2$ was employed with benzamide and benzyl alcohol 22

4-23 Comparison of reaction profiles ($MgSO_4$ and $Ba(OTf)_2$) 23

4-24 Full consumption of alcohol when benzamide is present 23

5 NMR Spectra 24

6 References 47

1 General Remarks

- ^{1}H NMR and ^{13}C NMR spectra were recorded on JEOL JMN-LA500 or 600 spectrometers operating at 500 MHz or 600 MHz (^{1}H) respectively. Chemical shifts are reported in parts per million (δ) and are reported relative to internal references of the deuterated solvent ($CDCl_3$ – 7.24/77.0 ppm, DMSO-d_6 – 2.50/39.5 ppm) or tetramethylsilane (0 ppm). Structures of known compounds were confirmed by comparison with commercially available compounds or data obtained from literature. Multiplicity: s = singlet, d = doublet, t = triplet, q = quartet, m = multiplet, br = broad.
- IR spectra were recorded on JASCO FT/IR-6100 spectrometer.
- Direct Analysis in Real Time (DART) mass spectra were recorded on JEOL JMS-T100TD mass spectrometer.
- Inductively coupled plasma (ICP) analysis was performed with Shimadzu ICPS-7510 equipment.
- Melting point was determined on a standard melting point apparatus and is uncorrected.
- STEM/EDS images were obtained using a JEOL JEM-2100F instrument operating at 200 kV. All STEM specimens were prepared by placing a drop of the suspension on carbon-coated Cu grids and dried in air (without staining).
- GC analysis was performed with Shimadzu GC-2010 instrument. Column = J & W SCIENTIFIC DB-1 0.25 mm ID, 0.25 m, 60.0 m; Gas pressure: 157.5 kPa, Total flow: 41.3 mL/min, Column flow: 0.93 mL/min, Velocity: 21.1 cm/sec; Purge flow: 3.0 mL/min; Split ratio: 40.1; Injector: 300 °C, FID: 300 °C; Column program: starting from 100 ˚C, 10 min hold, then 10 ˚C/min to 300 ˚C, 15 min hold.
- GCMS analysis was performed with Shimadzu GCMS-QP2010 Ultra instrument. Column = J & W SCIENTIFIC DB-1 0.25 mm ID, 0.25 m, 60.0 m; Gas pressure: 157.5 kPa, Total flow: 48.3 mL/min, Column flow: 1.10 mL/min, Velocity: 27.6 cm/sec; Purge flow: 3.0 mL/min; Split ratio: 40.1; Injector: 300 °C, FID: 300 °C; Column program: starting from 100 ˚C, 10 min hold, then 10 ˚C/min to 300 ˚C, 70 min hold.
- $AuClPPh_3$ and $Pd(OAc)_2$ were purchased from Strem Chemical Inc.
- $NaBH_4$ was purchased from Wako Pure Chemical Industries, recrystallised with diglyme by heating according to the literature[1] and stored in an argon-filled glove box. The entire recrystallisation process was carried out under argon atmosphere.
- Ketjenblack (carbon black) EC300J was purchased from Lion Corporation.
- Aqua regia for ICP sample preparation was made by mixing concentrated hydrochloric acid and concentrated nitric acid in a volume ratio of 3:1.
- Thin layer chromatography (TLC) was performed on TLC plates (TLC Silica gel 60 F_{254} glass plate) purchased from Merck KGaA and was visualised by UV light or by staining with 5–10% phosphomolybdic acid in ethanol followed by heating.
- Silica gel 60 from Merck KGaA was used for flash column chromatography with technical grade solvents.
- Preparative TLC (PTLC) was performed using Wakogel® B-5F from Wako Pure Chemical Industries.
- Dry and deoxidized toluene was purchased from Wako Pure Chemical Industries.
- Amides used in substrate scope were recrystallised before use.
- Benzyl alcohol and benzyl alcohol analogs were distilled before use.
- $MgSO_4$ was dried by heating it with over a Bunsen burner for at least 5 minutes before it was put under reduced pressure immediately. Afterwards, it was stored under argon until used.
- All other reagents were either purchased from Tokyo Chemical Industry (TCI), Sigma-Aldrich, Kanto Chemicals or Wako Pure Chemical Industries, and used as is without further purification.

Unless otherwise stated, all reactions were carried out under argon atmosphere.

2 Preparation of PI/CB-Au/Pd

2-1 Preparation of co-polymer used in the fabrication of PI/CB-Au/Pd

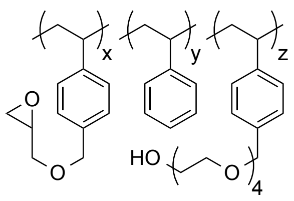

(x : y : z = 1 : 1 : 1)

2,2’-Azobis(4-methoxy)-2,4-dimethylvaleronitrile (V-70, 212.5 mg) was added to a stirred solution of styrene (2.4 g, 23.0 mmol), 4-vinylbenzyl glycidyl ether (4.4 g, 23.1 mmol) and 2-(2-(2-(2-(4-vinylbenzyloxy)ethoxy)ethoxy)ethoxy)ethanol (7.0 g, 22.6 mmol) in chloroform (12 mL). The mixture was then degassed by sonication under argon. After stirring for 48 hours at room temperature, the resulting viscous polymer solution was slowly poured into diethyl ether. The solvent layer was then decanted and the remaining precipitated polymer was washed with diethyl ether several times. The polymer was then dissolved in THF and the same precipitation, decantation and washing procedure was repeated two more times before being dried *in vacuo* to afford the co-polymer as a colourless viscous liquid (7.4 g, 54%).[2] The molar ratio of the components was determined by ^{1}H NMR analysis in $CDCl_3$ (x: y: z = 32 : 34 : 34). Note: The stabilisers in commercially available styrene were removed by passing it through a short column of basic alumina before use.

2-2 PI/CB-Au/Pd

Fabrication:

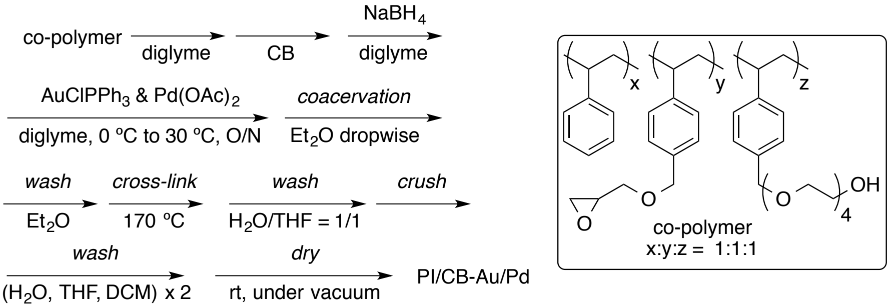

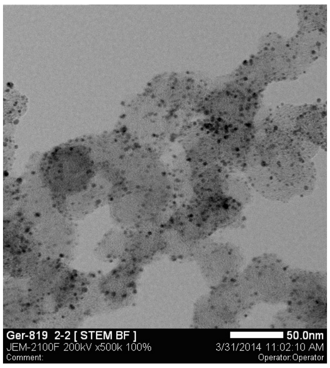

Diglyme (30 mL) and Ketjenblack EC300J (500.0 mg) were added to a 200 mL round bottom flask charged with the co-polymer (500.0 mg) and a stirring bar. Then, a diglyme solution of $NaBH_4$ (107.0 mg, 2.82 mmol) was added. The suspension was cooled in an ice-bath and stirred under open-air until the Ketjenblack was evenly dispersed. To this suspension was slowly added a diglyme solution (6 mL) of $AuClPPh_3$ (138.4 mg, 0.28 mmol) and $Pd(OAc)_2$ (63.0 mg, 0.28 mmol) mixed together. The ice-bath was removed and the mixture was allowed to stir overnight at 30 ºC. Diethyl ether (200 mL) was then added dropwise over one hour and the resulting mixture was vacuum-filtered. The black solid cake obtained at the top of the funnel was rinsed carefully with diethyl ether. The solid was crushed lightly with a mortar and pestle before being transferred to a 100 mL round bottom flask. The flask was flushed with argon and then placed in an oil bath (pre-heated to 170 ºC) for 4 h. After allowing the flask to cool to room temperature, THF and water (100 mL respectively) were added and the suspension was stirred vigorously overnight to remove boron. The suspension was then vacuum-filtered and the residue on top of the filter funnel was rinsed with water, THF and DCM in that order two times. The residue was then ground with a mortar and pestle before being dried *in vacuo* overnight to afford PI/CB-Au/Pd (1.01 g). Au loading: 0.240 mmol/g, Pd loading: 0.214 mmol/g.

Procedure for the preparation of the sample for ICP analysis:
Approximately 10 mg of PI/CB-Au/Pd measured carefully (amount measured was recorded) and heated to 200 ºC in H_2SO_4 (1 mL). HNO_3 was added dropwise until all solid had dissolved and no more brown fumes were observed. The mixture was cooled to room temperature and aqua regia (1 mL) was added slowly. The resulting mixture was made up to 50 mL with water in a volumetric flask and the resulting solution was subjected to ICP analysis.

STEM/EDS analysis:
STEM analysis indicated that metal nanoparticle size distribution was good and EDS mapping analysis indicated that alloyed bimetallic Au/Pd nanoparticles had formed.

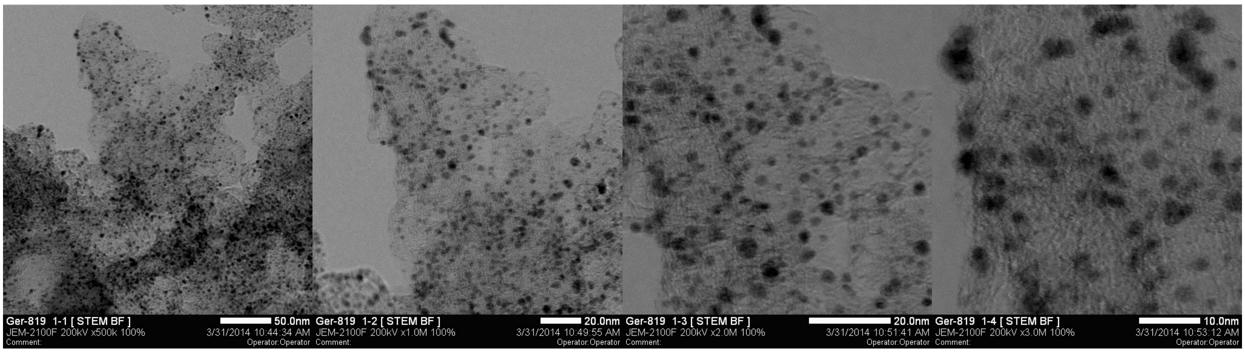

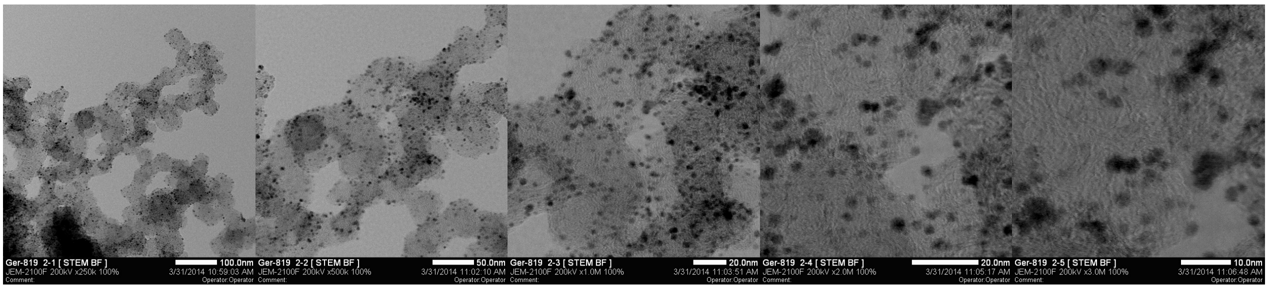

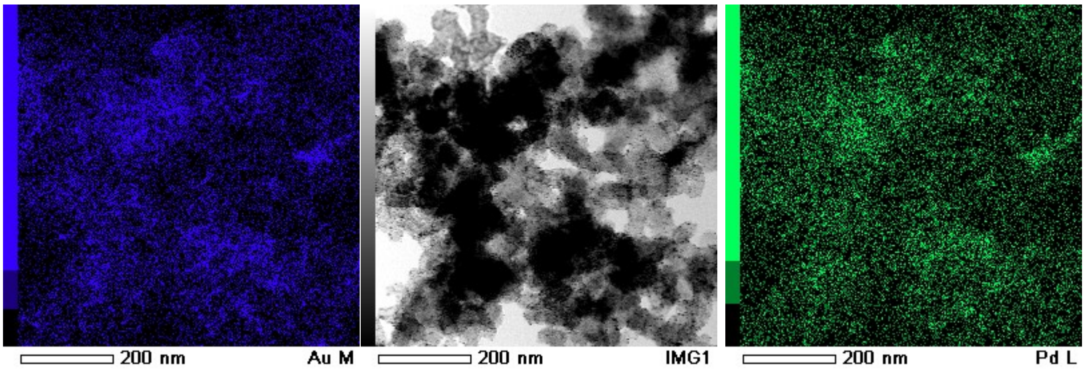

EDS Line analysis indicated that the ratio of Au:Pd within the bimetallic nanoparticles is approximately 1:1.

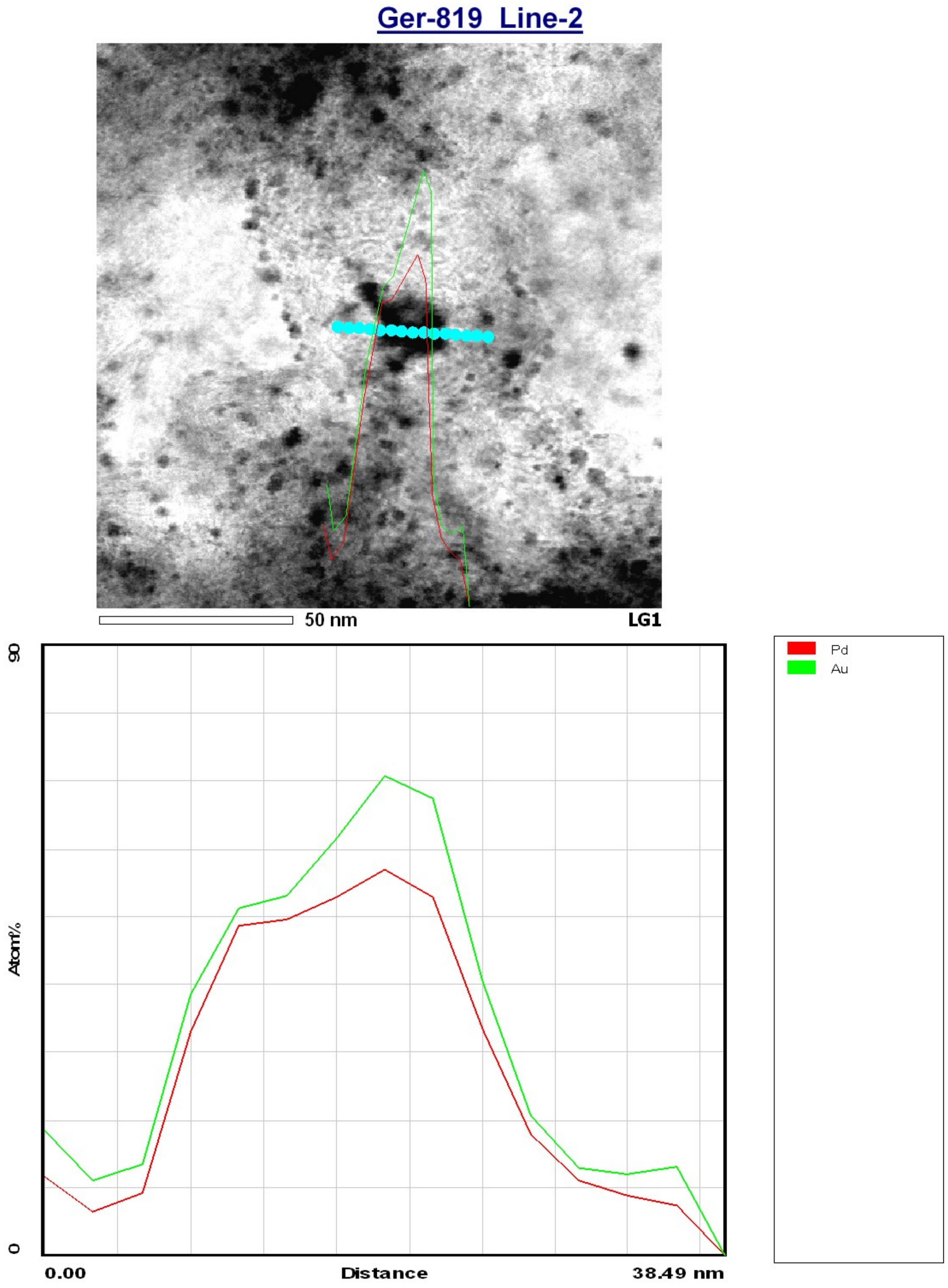

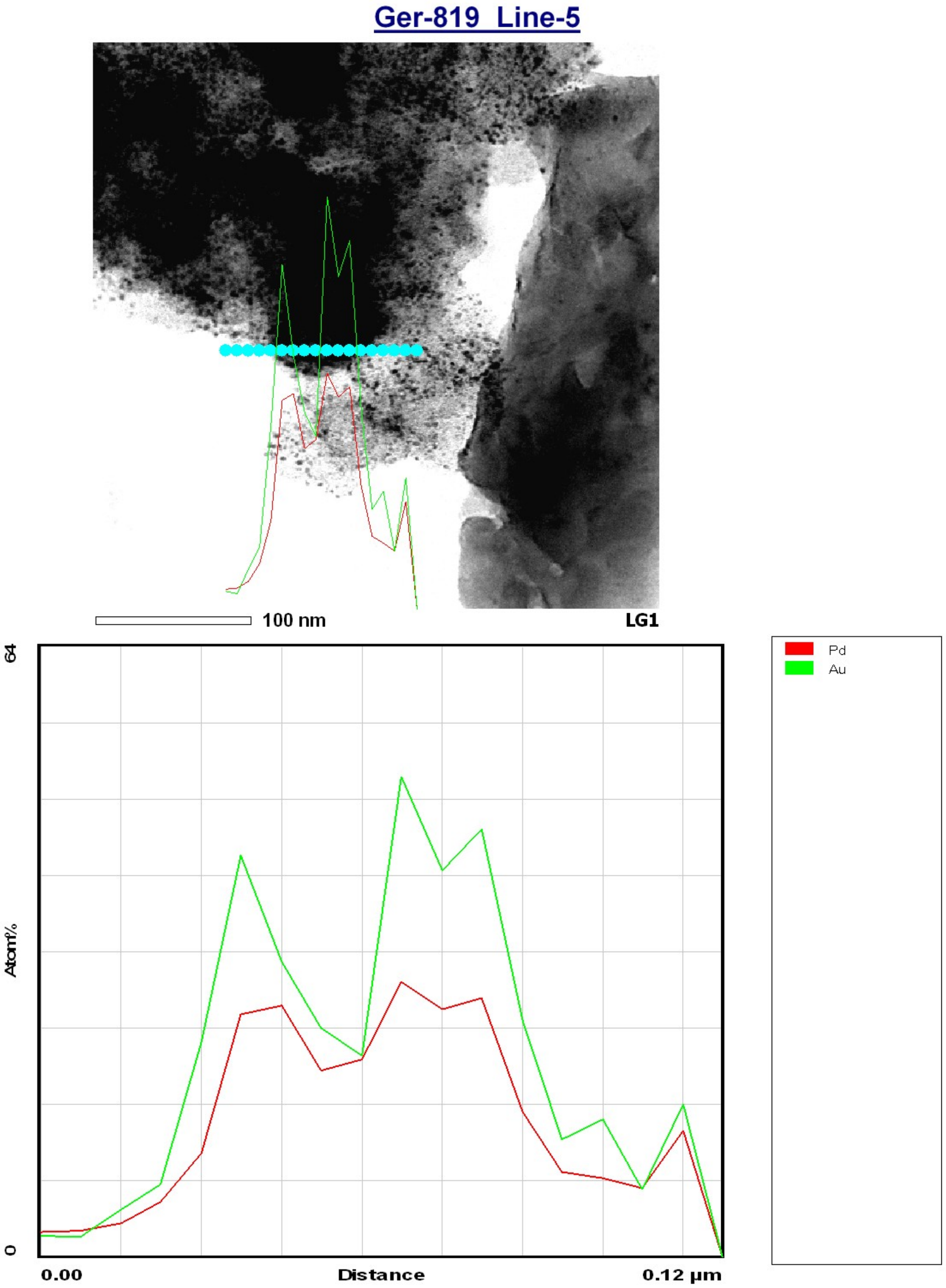
Ger-819 Line-5
100 nm
LG1
64
0
Atom%
0.00
Distance
0.12 µm
Pd
Au

3 Procedure for the *N*-alkylation of primary amides and characterisation of products

3-1 General procedure for the *N*-benzylation of benzamide (GC analysis)

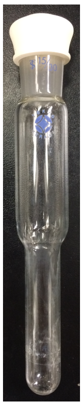

A Carousel® tube (image on the left) was charged with benzamide (30.3 mg, 0.25 mmol), PI/CB-Au/Pd (11.1 mg, 0.0025 mmol wrt Au) and a stirring bar. The carousel tube was then placed in an argon-filled glovebox. In the glovebox, additives, if any, were added (e.g. $Ca(OTf)_2$ (4.2 mg, 0.0125 mmol)) and the tube was sealed tightly with a septum. The septum was also taped tightly to the tube and the tube was then removed from the glovebox. Benzyl alcohol (81.1 mg, 0.75 mmol) and toluene (0.5 mL) were added, and then the part of the septum where the syringe needle went through was covered with tape. The tube was then placed on a Carousel® (parallel synthesiser from Radleys) that had been preheated to 150 ºC and it was left to stir for 18 hours, after which it was allowed to cool to room temperature. Ethyl acetate was added to dilute the mixture and the GC internal standard (dodecane) was added. An aliquot was passed through Celite into a GC phial and the clear solution was subjected to GC analysis.

3-2 N-Alkylation of primary amides and characterisation of products

General procedure as exemplified using benzamide and benzyl alcohol:

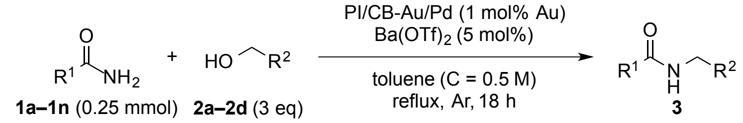

A Carousel® tube was charged with benzamide (**1a**, 30.5 mg, 0.25 mmol), PI/CB-Au/Pd (10.6 mg, 0.0025 mmol wrt Au) and a stirring bar. The carousel tube was then placed in an argon-filled glovebox. In the glovebox, $Ba(OTf)_2$ (5.1 mg, 0.0117 mmol) was added and the tube was sealed tightly with a septum. The septum was also taped tightly to the tube and the tube was then removed from the glovebox. Benzyl alcohol (**2a**, 81.0 mg, 0.75 mmol) and deoxidised toluene (0.5 mL) were added, and then the part of the septum where the syringe needle went through was covered with tape. The tube was then placed on a Carousel® (parallel synthesiser from Radleys) that had been preheated to 150 ºC and it was left to stir for 18 hours, after which it was allowed to cool to room temperature. Ethyl acetate was added to dilute the mixture and the catalyst was filtered off via vacuum filtration. The filtrate was concentrated and the crude obtained was purified by preparative TLC. After eluting with ethyl acetate, the solvent was removed via reduced pressure and then the pure product was dried under high vacuum for more than 1 day. White solid obtained (**3aa**, 50.5 mg, 95%).

N-benzylbenzamide (**3aa**)[3]

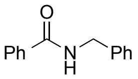

^{1}H NMR (600 MHz, $CDCl_3$): δ 7.79 (d, J = 7.5 Hz, 2H), 7.49 (t, J = 7.2 Hz, 1H), 7.42 (t, J = 7.7 Hz, 2H), 7.35 (d, J = 4.3 Hz, 4H), 7.30–7.28 (m, 1H), 6.51 (br s, 1H), 4.63 (d, J = 5.5 Hz, 2H); **^{13}C NMR (150 MHz, $CDCl_3$):** δ 167.3, 138.2, 134.3, 131.5, 128.8, 128.6, 127.9, 127.6, 126.9, 44.1.

N-benzyl-4-methylbenzamide (**3ba**)[3]

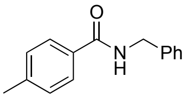

Used: 33.7 mg (0.25 mmol) of **1b**. Obtained: 54.8 mg (98%) of **3ba** as a white solid. **^{1}H NMR (600 MHz, $CDCl_3$):** δ 7.69 (d, J = 7.8 Hz, 2H), 7.37–7.34 (m, 4H), , 7.30–7.28 (m, 1H), 7.22 (d, J = 7.7 Hz, 2H), 6.44 (br s, 1H), 4.63 (d, J = 5.4 Hz, 2H), 2.39 (s, 3H); **^{13}C NMR (150 MHz, $CDCl_3$):** δ 167.3, 141.9, 138.3, 131.5, 129.2, 128.7, 127.9, 127.5, 126.9, 44.0, 21.4.

N-benzyl-4-methoxybenzamide (**3ca**)[4]

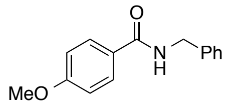

Used: 37.8 mg (0.25 mmol) of **1c**. Obtained: 60.9 mg (quant) of **3ca** as a white solid. **^{1}H NMR (600 MHz, CDCl$_3$):** δ 7.76 (d, J = 8.3 Hz, 2H), 7.34–7.29 (m, 4H), 7.31–7.28 (m, 1H), 6.91 (d, J = 8.3 Hz, 2H), 6.40 (br s, 1H), 4.63 (d, J = 5.6 Hz, 2H), 3.84 (s, 3H); **^{13}C NMR (150 MHz, CDCl$_3$):** δ 166.8, 162.2, 138.4, 128.7, 127.9, 127.5, 126.6, 113.7, 55.4, 44.0.

N-benzyl-2-ethoxybenzamide (**3da**)

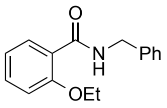

Used: 41.1 mg (0.25 mmol) of **1d**. Obtained: 56.5 mg (89%) of **3da** as a colourless oil. **^{1}H NMR (600 MHz, CDCl$_3$):** δ 8.34 (br s, 1H), 8.25 (dd, J = 7.8, 1.9 Hz, 1H), 7.42–7.34 (m, 5H), 7.29–7.26 (m, 1H), 7.06 (t, J = 7.5 Hz, 1H), 6.91 (d, J = 8.3 Hz, 1H), 4.66 (d, J = 5.4 Hz, 2H), 4.10 (q, J = 7.0 Hz, 2H), 1.31 (t, J = 7.0 Hz, 3H); **^{13}C NMR (150 MHz, CDCl$_3$):** δ 165.1, 156.9, 138.4, 132.6, 132.2, 128.5, 127.7, 127.3, 121.2, 121.1, 112.1, 64.5, 43.9, 14.5; **HRMS (DART):** calculated for $C_{16}H_{18}NO_2^+$ [M+H$^+$] 256.13375, found 256.13403; **IR (neat):** 3388, 2980, 1654, 1601, 1531, 1452, 1297, 1235, 1162, 1116, 1035, 755, 700 cm^{-1}.

N-benzyl-salicylamide (**3ea**)[5]

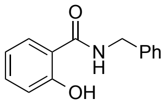

Used: 34.5 mg (0.25 mmol) of **1e**. Obtained: 30.2 mg (53%) of **3ea** as a white solid. **^{1}H NMR (500 MHz, CDCl$_3$):** δ 12.31 (s, 1H), 7.42–7.32 (m, 7H), 7.00 (dd, J = 8.4, 0.7 Hz, 1H), 6.83 (ddd, J = 8.1, 7.0, 0.9 Hz, 1H), 6.57 (br s, 1H), 4.63 (d, J = 5.7 Hz, 2H); **^{13}C NMR (125 MHz, CDCl$_3$):** δ 169.8, 161.6, 137.4, 134.3, 128.9, 127.9, 125.3, 118.6, 114.0, 43.6.

N-benzyl-4-fluorobenzamide (**3fa**)[6]

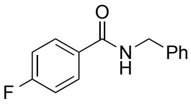

Used: 34.6 mg of **1f**. Obtained: 51.3 mg (90%) of **3fa** as a white solid. **^{1}H NMR (600 MHz, CDCl$_3$):** δ 7.81–7.79 (m, 2H), 7.38–7.30 (m, 5H), 7.10 (t, J = 8.5 Hz, 2H), 6.37 (br s, 1H), 4.64 (d, J = 5.6 Hz, 2H); **^{13}C NMR (150 MHz, CDCl$_3$):** δ 166.3, 164.7 (d, J = 248.3 Hz), 138.0, 130.51, 129.3 (d, J = 8.5 Hz), 128.8, 128.0, 127.7, 115.6 (d, J = 21.7 Hz), 44.2; **^{19}F NMR (600 MHz, CDCl$_3$):** δ –108.0.

N-benzylpicolinamide (**3ga**)[4]

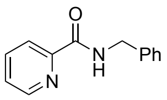

Used: 30.8 mg (0.25 mmol) of **1g** and 135.1 mg (1.25 mmol) of **2a**. Obtained: 33.6 mg (63%) of **3ga** as an off-white solid. **^{1}H NMR (500 MHz, CDCl$_3$):** δ 8.52 (d, J = 4.8 Hz, 1H), 8.40 (br s, 1H), 8.24 (d, J = 7.8 Hz, 1H), 7.85 (td, J = 7.7, 1.7 Hz, 1H), 7.42 (ddd, J = 7.6, 4.7, 1.1 Hz, 1H), 7.38–7.26 (m, 5H), 4.68 (d, J = 6.1 Hz, 2H); **^{13}C NMR (125 MHz, CDCl$_3$):** δ 164.2, 149.8, 148.0, 138.2, 137.3, 128.7, 127.8, 127.4, 126.2, 122.3, 43.4.

N-benzylnicotinamide (**3ha**)[7]

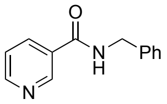

Used: 30.8 mg (0.25 mmol) of **1h** with twice the amount of catalyst and Ba(OTf)$_2$. Obtained: 28.1 mg (52%) of **3ha** as a white solid. **^{1}H NMR (600 MHz, CDCl$_3$):** δ 8.94 (br s, 1H), 8.63 (br s, 1H), 8.13 (d, J = 7.9 Hz, 1H), 7.35–7.27 (m, 6H), 7.09 (br s, 1H), 4.62 (d, J = 5.5 Hz, 2H); **^{13}C NMR (150 MHz, CDCl$_3$):** δ 165.4, 151.9, 147.8, 137.7, 135.4, 130.1, 128.7, 127.9, 127.7, 123.5, 44.1.

N-benzylacetamide (**3ia**)[4]

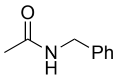

Used: 14.9 mg (0.25 mmol) of **1i** and 134.3 mg (1.24 mmol) of **2a**. Obtained: 28.8 mg (77%) of **3ia** as a white solid. **^{1}H NMR (500 MHz, CDCl$_3$):** δ 7.34–7.26 (m, 5H), 6.10 (br s, 1H), 4.40 (d, J = 5.7 Hz, 2H), 1.99 (s, 3H); **^{13}C NMR (125 MHz, CDCl$_3$):** δ 170.0, 138.2, 128.6, 127.8, 127.4, 43.6, 23.1.

N-benzylhexanamide (**3ja**)[8]

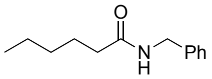

Used: 28.7 mg (0.25 mmol) of **1j**. Obtained: 48.8 mg (95%) of **3ja** as a white solid. **^{1}H NMR (500 MHz, CDCl$_3$):** *δ* 7.33–7.24 (m, 5H), 6.14 (br s, 1H), 4.39 (d, *J* = 5.8 Hz, 2H), 2.18 (t, *J* = 7.7 Hz, 2H), 1.66–1.60 (m, 2H), 1.33–1.26 (m, 4H), 0.88 (t, *J* = 6.7 Hz, 3H); **^{13}C NMR (125 MHz, CDCl$_3$):** *δ* 173.1, 138.4, 128.5, 127.6, 127.3, 43.4, 36.6, 31.4, 25.4, 22.3, 13.9.

N-benzylisobutyramide (**3ka**)[9]

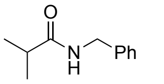

Used: 21.5 mg (0.25 mmol) of **1k**. Obtained: 41.1 mg (94%) of **3ka** as a white solid. **^{1}H NMR (500 MHz, CDCl$_3$):** *δ* 7.32–7.24 (m, 5H), 6.08 (br s, 1H), 4.40 (d, *J* = 5.7 Hz, 2H), 2.39 (m, *J* = 6.8 Hz, 1H), 1.16 (d, *J* = 6.9 Hz, 6H); **^{13}C NMR (125 MHz, CDCl$_3$):** *δ* 176.8, 138.5, 128.6, 127.6, 127.3, 43.3, 35.5, 19.5.

N-benzylpivalamide (**3la**)[10]

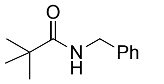

Used: 25.6 mg (0.25 mmol) of **1l**. Obtained: 43.6 mg (90%) of **3la** as a white solid. **^{1}H NMR (500 MHz, CDCl$_3$):** *δ* 7.35–7.25 (m, 5H), 5.96 (br s, 1H), 4.44 (d, *J* = 5.6 Hz, 2H), 1.23 (s, 9H); **^{13}C NMR (125 MHz, CDCl$_3$):** *δ* 178.3, 138.6, 128.7, 127.6, 127.4, 43.5, 38.7, 27.6.

N-benzyl-2-phenylacetamide (**3ma**)[8]

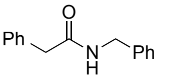

Used: 33.9 mg (0.25 mmol) of **1m**. Obtained: 51.6 mg (91%) of **3ma** as a white solid. **^{1}H NMR (500 MHz, CDCl$_3$):** *δ* 7.34–7.22 (m, 8H), 7.16 (d, *J* = 7.4 Hz, 2H), 5.88 (br s, 1H), 4.39 (d, *J* = 5.8 Hz, 2H), 3.59 (s, 2H); **^{13}C NMR (125 MHz, CDCl$_3$):** *δ* 170.8, 138.1, 134.7, 129.4, 129.0, 128.6, 127.4, 127.33, 127.30, 43.7, 43.5.

N-benzylcyclohexanecarboxamide (**3na**)[4]

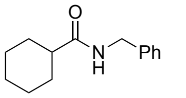

Used: 32.1 mg (0.25 mmol) of **1n**. Obtained: 57.1 mg (quant) of **3na** as a white solid. **^{1}H NMR (500 MHz, CDCl$_3$):** *δ* 7.33–7.23 (m, 5H), 6.04 (br s, 1H), 4.40 (d, *J* = 5.8 Hz, 2H), 2.11 (tt, *J* = 11.9, 3.6 Hz, 1H), 1.88–1.85 (m, 2H), 1.80–1.76 (m, 2H), 1.67–1.64 (m, 1H), 1.49–1.41 (m, 2H), 1.29–1.18 (m, 3H); **^{13}C NMR (125 MHz, CDCl$_3$):** *δ* 175.9, 138.5, 128.5, 127.6, 127.3, 45.4, 43.2, 29.6, 25.6.

N-(4-methylbenzyl)benzamide (**3ab**)[7]

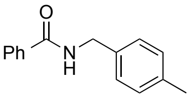

Used: 30.3 mg (0.25 mmol) of **1a** and 91.2 mg (0.75 mmol) of **2b**. Obtained: 56.9 mg (quant) of **3ab** as a white solid. **^{1}H NMR (500 MHz, CDCl$_3$):** *δ* 7.78 (d, *J* = 7.8 Hz, 2H), 7.50–7.47 (m, 1H), 7.42–7.39 (m, 2H), 7.24 (d, *J* = 7.7 Hz, 2H), 7.15 (d, *J* = 7.7 Hz, 2H), 6.50 (br s, 1H), 4.58 (d, *J* = 5.6 Hz), 2.34 (s, 3H); **^{13}C NMR (125 MHz, CDCl$_3$):** *δ* 167.3, 137.3, 135.1, 134.4, 131.4, 129.4, 128.5, 127.9, 126.9, 43.8, 21.1.

N-((methyl 4-carboxylate)benzyl)benzamide (**3ad**)

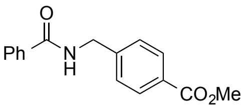

Used: 30.6 mg (0.25 mmol) of **1a** and 124.2 mg (0.75 mmol) of **2d**. Obtained: 41.1 mg (61%) of **3ad** as a white solid after two purifications. However, a small amount (ratio of impurity to product is 0.065 : 1) of **2d** still remained in the product as an impurity. **^{1}H NMR (500 MHz, CDCl$_3$):** *δ* 7.98 (d, *J* = 8.1 Hz, 2H), 7.81 (d, *J* = 7.8 Hz, 2H), 7.51–7.49 (m, 1H), 7.43–7.37 (m, 4H), 6.83 (br s, 1H), 4.67 (d, *J* = 5.9 Hz, 2H), 3.90 (s, 3H); **^{13}C NMR (125 MHz, CDCl$_3$):** *δ* 167.5, 166.8, 143.5, 134.0, 131.7, 130.0, 129.3, 128.6, 127.5, 127.0, 52.1, 43.6; **HRMS (DART):** calculated for $C_{16}H_{16}NO_3^+$ [*M*+H$^+$] 270.11302, found 270.11295; **IR (KBr):** 3298, 1713, 1639, 1543, 1317, 1283, 1113, 754, 698 cm^{-1}; **m.p.** = 142–146 °C.

N-(4-methylbenzyl)hexanamide (**3jb**)

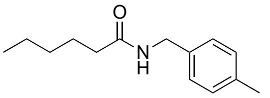

Used: 28.6 (0.25 mmol) of **1j** and 91.9 mg (0.75 mmol) of **2b**. Obtained: 47.1 mg (86%) of **3jb** as a white solid. **^{1}H NMR (500 MHz, $CDCl_3$):** δ 7.15 (q, J = 7.8 Hz, 4H), 5.83 (br s, 1H), 4.38 (d, J = 5.6 Hz, 2H), 2.33 (s, 3H), 2.18 (t, J = 7.7 Hz, 2H), 1.64 (m, J = 7.3 Hz, 2H), 1.33–1.28 (m, 4H), 0.89 (t, J = 6.7 Hz, 3H); **^{13}C NMR (125 MHz, $CDCl_3$):** δ 172.9, 137.1, 135.4, 129.3, 127.8, 43.3, 36.7, 31.4, 25.4, 22.3, 21.0, 13.9; **HRMS (DART):** calculated for $C_{14}H_{22}NO^+$ [M+H^+] 220.17014, found 220.16931; **IR (KBr):** 3291, 2953, 2928, 2869, 1634, 1545, 1462, 1422, 1214, 803, 719 cm^{-1}; **m.p.** = 63–65 ºC.

N-(4-methoxybenzyl)hexanamide (**3jc**)

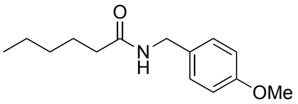

Used: 29.0 mg (0.25 mmol) of **1j** and 104.1 (0.75 mmol) of **2c**. Obtained: 26.0 mg (44%) of **3jc** as a white solid. **^{1}H NMR (500 MHz, $CDCl_3$):** δ 7.20 (d, J = 8.4 Hz, 2H), 6.86 (d, J = 8.4 Hz, 2H), 5.78 (br s, 1H), 4.36 (d, J = 5.6 Hz, 2H), 3.79 (s, 3H), 2.18 (t, J = 7.6 Hz, 2H), 1.64 (m, J = 7.3 Hz, 2H), 1.34–1.28 (m, 4H), 0.89 (t, J = 6.7 Hz, 3H); **^{13}C NMR (125 MHz, $CDCl_3$):** δ 172.9, 158.9, 130.5, 129.1, 114.0, 55.2, 43.0, 36.7, 31.4, 25.4, 22.4, 13.9; **HRMS (DART):** calculated for $C_{14}H_{22}NO_2^+$ [M+H^+] 236.16505, found 236.16484; **IR (KBr):** 3290, 2954, 2930, 2855, 1632, 1554, 1513, 1435, 1255, 1174, 1110, 1031, 814, 758 cm^{-1}; **m.p.** = 86–89 ºC.

N-((methyl 4-carboxylate)benzyl)hexanamide (**3jd**)

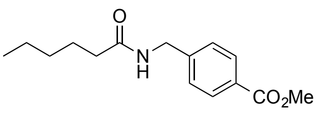

Used: 28.9 mg (0.25 mmol) of **1j** and 124.5 (0.75 mmol) of **2d**. Obtained: 45.1 mg (68%) of **3jd** as a white solid. **^{1}H NMR (500 MHz, $CDCl_3$):** δ 7.96 (d, J = 8.0 Hz, 2H), 7.30 (d, J = 8.0 Hz, 2H), 6.28 (br s, 1H), 4.46 (d, J = 6.0 Hz, 2H), 3.90 (s, 3H), 2.22 (t, J = 7.4 Hz, 2H), 1.65 (m, J = 7.7 Hz, 2H), 1.32–1.28 (m, 4H), 0.89 (t, J = 6.6 Hz, 3H); **^{13}C NMR (125 MHz, $CDCl_3$):** δ 173.3, 166.8, 143.8, 129.8, 129.1, 127.4, 52.0, 43.0, 36.5, 31.4, 25.4, 22.3, 13.9; **HRMS (DART):** calculated for $C_{15}H_{22}NO_3^+$ [M+H^+] 264.15997, found 264.15866; **IR (KBr):** 3289, 2954, 2929, 2869, 1719, 1639, 1548, 1433, 1284, 1221, 1191, 1117, 1021, 851, 758, 699 cm^{-1}; **m.p.** = 87–90 ºC.

3-3 Recovery and reuse of catalyst & leaching test

At the end of the reaction, the Carousel® tube was removed from the Carousel® parallel synthesiser and allowed to cool to room temperature. The crude (with catalyst) was then diluted with ethyl acetate and spiked with dodecane (GC internal standard). An aliquot was passed through a thin pad of Celite into a GC vial for GC analysis. The remaining solution/suspension was vacuum filtered carefully with 8 mm filter paper (made for the Kiriyama funnel) to separate the catalyst from the organic compounds. The catalyst was then washed carefully with ethyl acetate on top of the funnel (with the vacuum still turned on) and then dried *in vacuo* while it was still on the filter paper and funnel. After 1 day, the recovered catalyst was transferred carefully to a new Carousel® tube for the next run. In the case of $Ba(OTf)_2$, an appropriate amount of additional $Ba(OTf)_2$ was added in each run. On the other hand, in the case of $Ca(OTf)_2$, no additional $Ca(OTf)_2$ was added in the subsequent runs after the first.

For the reactivation of the catalyst, after the transfer of the recovered catalyst to a new Carousel® tube, the catalyst was heated by lowering the tube (unsealed) into a 170 ºC oil bath for 5 hours and then cooled to room temperature before reagents were added.
The scale of the reaction was changed according to the amount of catalyst recovered from each run.

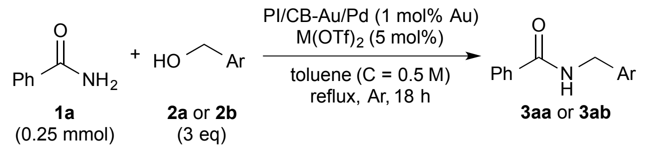

**2a**; M	**run**	1[b]	2[b]	3[b]	4[b, c]	5[b]	6[b]	7[b, c]	8[b]	9[b]	10[b, c]	11[b]
= Ba	**yield (%)[a]**	>99	99	53	>99	>99	99	>99	>99	95	>99	>99
**2b**; M	**run**	1	2[d]	3[d]	4[c, d]	5[d]						
= Ca	**yield (%)[a]**	93	95	93	89	93						

[a] Determined by GC analysis with dodecane as the internal standard. [b] No leaching of Au or Pd was detected (under detection limit; determined by ICP analysis). [c] The recovered catalyst from the previous run was reactivated before use. [d] Recovered catalyst was treated with DCM and no additional Lewis acid was added for the new run.

Work-up procedures using only dichloromethane:

The crude mixture (with catalyst) was diluted with dichloromethane and dodecane (GC internal standard) was added. An aliquot was passed through a thin pad of Celite into a GC vial for GC analysis. The remaining solution/suspension was vacuum-filtered carefully with 8 mm filter paper (made for the Kiriyama funnel) to separate the catalyst from the organic compounds. The catalyst was then washed carefully with dichloromethane on top of the funnel (with the vacuum still turned on) and then dried *in vacuo* while it was still on the filter paper and funnel. The subsequent procedures are the same as those described above.

Determination of metal(s) leaching during reaction

The filtrate obtained from each run was concentrated and then diluted with a small amount of MeOH. The methanol solution was then passed through a membrane filter (0.25 or 0.45 μm) into a new test tube. The solvent was then removed under reduced pressure. The solid obtained in the test tube was then heated to 200 °C and 0.2 mL of concentrated H_2SO_4 was added. After further heating for 1 hour, drops of concentrated HNO_3 were added at regular intervals (45~60 min) until the resulting solution was lightly coloured (yellow or orange, not brown) and clear. Brown fumes should not be observed at this point when addition HNO_3 is added. If brown fumes are still observed, the addition of HNO_3 at regular intervals should be continued.

To the cooled clear solution obtained, 0.2 mL of aqua regia was added and the resulting mixture was shaken lightly at first and then more vigorously. It was then made up to 10 mL with water and the resulting diluted solution was filtered into a vial to remove any remaining solids. The clear solution was then subjected to ICP analysis.

4 Additional data

4-1 Screening of PI/CB catalysts

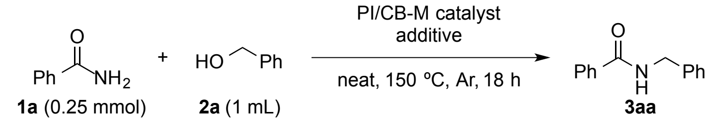

entry	M[a]	additive	yield[b] [%]
1	Ir, Ru, Rh, Ni or Co	–	n.d.[c]
2	Au	–	n.d.[c]
3	Pd	–	trace[c]
4[d]	Au	–	0
5[d]	Pd	–	7
6[d]	Au (2 mol%) + Pd (2 mol%)	–	24
7[d]	Au/Pd (Au:Pd = 1:1)	–	43
**8[d]**	**Au/Pd (Au:Pd = 1:1)**	**$MgSO_4$ (50 mg)**	**89**
9[d]	–	$MgSO_4$ (50 mg)	0
10[d]	–	–	0

[a] Catalyst loading was set to 2 mol%. In the case of bimetallic catalysts, the catalyst loading was set to 2 mol% with respect to the first metal stated. [b] Determined by GC analysis with dodecane as the internal standard. [c] Determined by GCMS analysis of crude after the stipulated reaction time (n.d. = not detected). [d] Deoxidised benzyl alcohol was used.

4-2 Optimisation with $MgSO_4$

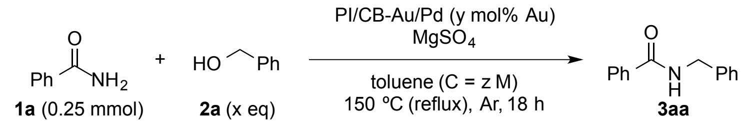

entry	x	y	$MgSO_4$	z	yield[a] [%]
1[b]	4	2	50 mg	0.25	90
2	4	1	50 mg	0.25	76
3	4	2	25 mg	0.25	75
4	4	2	10 mg	0.25	55
5[c]	4	2	50 mg	0.25	95
6	4	2	50 mg	0.5	quant
**7[d]**	**4**	**1**	**50 mg**	**0.5**	**quant**
8	3	1	50 mg	0.5	91
9	2	1	50 mg	0.5	59
10	1.1	1	50 mg	0.5	33

[a] Determined by GC analysis with dodecane as the internal standard. [b] Average of 3 experiments. [c] Reaction time = 24 hours. [d] Average of 2 experiments.

4-3 Screening of additives

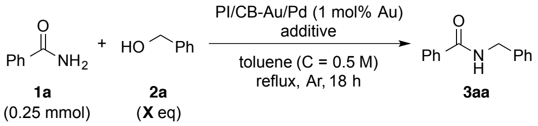

entry	additive	amount	X = 4[a]	X = 3[a]
1[b]	$MgSO_4$	1.66 eq	quant	91
2	MgX_2 (X = Fl, Cl, Br)	1.66 eq	1–17	–
3	MgO	1.66 eq	31	–
4	$Mg(OH)_2$	1.66 eq	31	–
5	$Mg(OTf)_2$	1.66 eq	97	–
6	$Mg(OTf)_2$	0.5 eq	quant	–
7	$Mg(OTf)_2$	5 mol%	quant	quant (64)[d]
8	$Ca(OTf)_2$	5 mol%	99	quant (63)[d]
**9**	**$Ba(OTf)_2$**	**5 mol%**	**98**	**quant (95)[c] (85)[d] (94)[e]**
10	LiOTf	5 mol%	quant	90
11	NaOTf	5 mol%	98	–
12	KOTf	5 mol%	73	–
13	$Sc(OTf)_3$	5 mol%	98	92
14	$Yb(OTf)_3$	5 mol%	quant	quant
15	TfOH	5 mol%	70	–
16[f]	MS 3A or MS 4A	20 mg	<10	–
17[f]	MS 5A	20 mg	87	–
18	–	–	50	–

[a] Yield was determined by GC analysis with dodecane as the internal standard. [b] Average of 2 experiments. [c] 2.5 eq of benzyl alcohol were used. [d] GC yields obtained when the reaction was conducted at 120 °C (hot plate temperature), [e] 4-Methylbenzyl alcohol used as the substrate; a 5:1 ratio of toluene:H_2O was used as the solvent, [f] Catalyst loading: 2 mol% Au; solvent: xylene (C = 0.25 M).

4-4 Possible disproportionation-like reaction during the initiation stages of the reaction

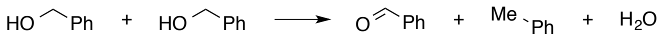

4-5 Control experiment (1) – Reaction with water

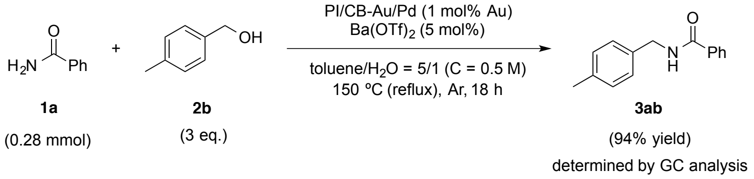

A Carousel® tube was charged with PI/CB-Au/Pd (12.0 mg, 0.0025 mmol wrt Au), 4-Methylbenzyl alcohol (99.6 mg, 0.84 mmol), benzamide (33.8 mg, 0.28 mmol) and a stirring bar. The carousel tube was then placed in an argon-filled glovebox. In the glovebox, $Ba(OTf)_2$ (5.7 mg, 0.013 mmol) was added and the tube was sealed tightly with a septum. The septum was also taped tightly to the tube and the tube was then removed from the glovebox. Deoxidized toluene (0.5 mL) and water (0.1 mL) were added, and then the part of the septum where the syringe needle went through was covered with tape. The tube was then placed on a Carousel® (parallel synthesiser from Radleys) that had been preheated to 150 ºC and it was left to stir for 18 hours, after which it was allowed to cool to room temperature. Ethyl acetate was added to dilute the mixture and the GC internal standard (dodecane) was added. An aliquot was passed through Celite into a GC phial and the clear solution was subjected to GC analysis.

4-6 Control experiment (2) – Reductive amidation (Formation of *N,N'*-(*p*-methylphenylmethylene)dibenzamide (*N,N'*-diamide))

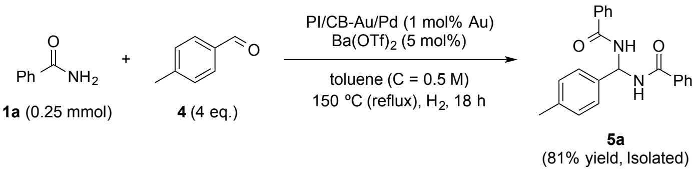

A Carousel® tube was charged with PI/CB-Au/Pd (12.5 mg, 0.0025 mmol wrt Au), benzamide (31.3 mg, 0.26 mmol) and a stirring bar. The carousel tube was then placed in an argon-filled glovebox. In the glovebox, $Ba(OTf)_2$ (6.2 mg, 0.014 mmol) was added. Deoxidised toluene (0.5 mL) and 4-methylbenzaldehyde (119.6 mg, 0.98 mmol) was added, and then a three-way cock was attached to the tube. A balloon filled with H_2 was attached and the tube was flushed with hydrogen gas 10 times. The tube was then placed on a Carousel® (parallel synthesiser from Radleys) that had been preheated to 150 ºC and it was left to stir for 18 hours, after which it was allowed to cool to room temperature. 10 mL of 1 to 1 mixture of ethyl acetate and hexane was added to dilute the mixture and a suspension of a white solid was obtained. The suspension was then vacuum-filtered and the residue on top of the filter funnel was rinsed with 1 to 1 mixture of ethyl acetate and hexane. The residue was collected and dried under vacuum. *N,N'*-(*p*-Tolylmethylene)dibenzamide (**5a**) was obtained as white solid (35.9 mg, 81%). No *N*-(4-methylbenzyl)benzamide (**3ab**) was obtained.

^{1}H NMR (600 MHz, DMSO-d$_6$): δ 8.94 (d, J = 7.8 Hz, 2H), 7.90 (d, J = 7.7 Hz, 4H), 7.56 (t, J = 7.3 Hz, 2H), 7.48 (t, J = 7.7 Hz, 4H), 7.36 (d, J = 7.8 Hz, 2H), 7.19 (d, J = 7.8 Hz, 2H), 7.01 (t, J = 8.0 Hz, 1H), 2.30 (s, 3H); **^{13}C NMR (150 MHz, DMSO-d$_6$):** δ 165.4, 137.4, 136.8, 133.9, 131.5, 128.8, 128.3, 127.4, 126.3, 58.5, 20.6.[11]

4-7 Control experiment (3) –Formation of *N,N'*-(*p*-methylphenylmethylene)dibenzamide (*N,N'*-diamide)

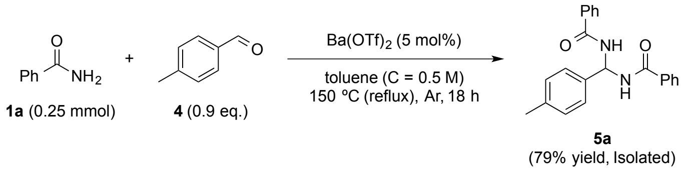

A Carousel® tube was charged with benzamide (31.5 mg, 0.26 mmol) and a stirring bar. The carousel tube was then placed in an argon-filled glovebox. In the glovebox, $Ba(OTf)_2$ (6.0 mg, 0.014 mmol) was added and the tube was sealed tightly with a septum. The septum was also taped tightly to the tube and the tube was then removed from the glovebox. 4-Methylbenzaldehyde (28.6 mg, 0.24 mmol) and deoxidized toluene (0.5 mL) were added, and then the part of the septum where the syringe needle went through was covered with tape. The tube was then placed on a Carousel® (parallel synthesiser from Radleys) that had been preheated to 150 ºC and it was left to stir for 18 hours, after which it was allowed to cool to room temperature. 10 mL of 1 to 1 mixture of ethyl acetate and hexane was added to dilute the mixture and white solid suspension was obtained. The suspension was then vacuum-filtered and the residue on top of the filter funnel was rinsed with 1 to 1 mixture of ethyl acetate and hexane. The residue was collected and dried under vacuum. *N,N'*-(*p*-methylphenylmethylene)dibenzamide (*N,N'*-diamide) (**5a**) was obtained as white solid (35.4 mg, 79% based on the amount of benzamide).

4-8 Control experiment (4) –Formation of *N,N*'-(*p*-methylphenylmethylene)dibenzamide (*N,N*'-diamide)

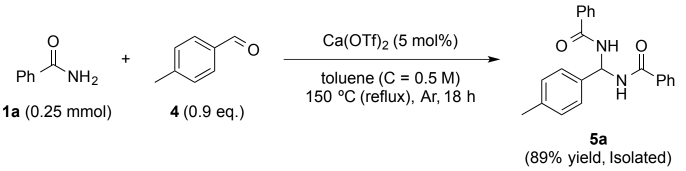

A Carousel® tube was charged with benzamide (31.8 mg, 0.26 mmol) and a stirring bar. The carousel tube was then placed in an argon-filled glovebox. In the glovebox, $Ca(OTf)_2$ (4.0 mg, 0.012 mmol) was added and the tube was sealed tightly with a septum. The septum was also taped tightly to the tube and the tube was then removed from the glovebox. 4-Methylbenzaldehyde (28.6 mg, 0.24 mmol) and deoxidized toluene (0.5 mL) were added, and then the part of the septum where the syringe needle went through was covered with tape. The tube was then placed on a Carousel® (parallel synthesiser from Radleys) that had been preheated to 150 ºC and it was left to stir for 18 hours, after which it was allowed to cool to room temperature. 10 mL of 1 to 1 mixture of ethyl acetate and hexane was added to dilute the mixture and a white solid suspension was obtained. The suspension was then vacuum-filtered and the residue on top of the filter funnel was rinsed with 1 to 1 mixture of ethyl acetate and hexane. The residue was collected and dried under vacuum. *N,N*'-(*p*-methylphenylmethylene)dibenzamide (*N,N*'-diamide) (**5a**) was obtained as white solid (40.1 mg, 89% based on the amount of benzamide).

4-9 Control experiment (5) –Formation of *N,N*'-(*p*-methylphenylmethylene)dibenzamide (*N,N*'-diamide)

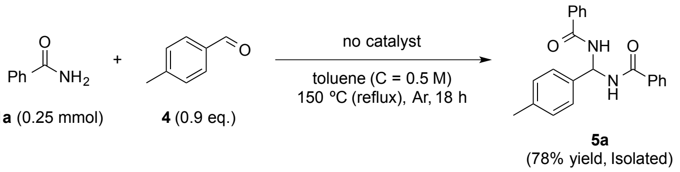

A Carousel® tube was charged with benzamide (31.3 mg, 0.26 mmol) and a stirring bar. The carousel tube was then placed in an argon-filled glovebox. In the glovebox, the tube was sealed tightly with a septum. The septum was also taped tightly to the tube and the tube was then removed from the glovebox. 4-Methylbenzaldehyde (28.6 mg, 0.24 mmol) and deoxidized toluene (0.5 mL) were added, and then the part of the septum where the syringe needle went through was covered with tape. The tube was then placed on a Carousel® (parallel synthesiser from Radleys) that had been preheated to 150 ºC and it was left to stir for 18 hours, after which it was allowed to cool to room temperature. 10 mL of 1 to 1 mixture of ethyl acetate and hexane was added to dilute the mixture and a white solid suspension was obtained. The suspension was then vacuum-filtered and the residue on top of the filter funnel was rinsed with 1 to 1 mixture of ethyl acetate and hexane. The residue was collected and dried under vacuum. *N,N*'-(*p*-methylphenylmethylene)dibenzamide (*N,N*'-diamide) (**5a**) was obtained as white solid (34.7 mg, 78% based on the amount of benzamide).

4-10 Control experiment (6) – Transfer hydrogenation from the *N,N*'-diamide

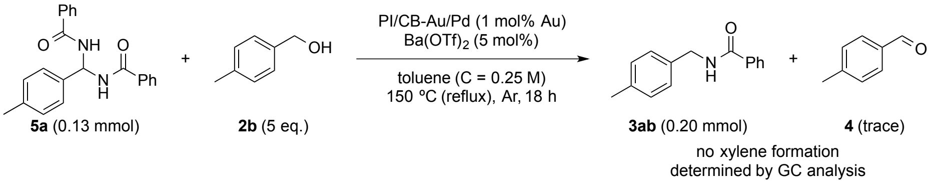

A Carousel® tube was charged with PI/CB-Au/Pd (10.3 mg, 0.0025 mmol wrt Au), 4-Methylbenzyl alcohol (86.0 mg, 0.70 mmol), *N,N*'-(*p*-Tolylmethylene)dibenzamide (45.0 mg, 0.13 mmol) and a stirring bar. The carousel tube was then placed in an argon-filled glovebox. In the glovebox, $Ba(OTf)_2$ (7.7 mg, 0.017 mmol) was added and the tube was sealed tightly with a septum. The septum was also taped tightly to the tube and the tube was then removed from the glovebox. Deoxidized toluene (0.5 mL) were added, and then the part of

the septum where the syringe needle went through was covered with tape. The tube was then placed on a Carousel® (parallel synthesiser from Radleys) that had been preheated to 150 ºC and it was left to stir for 18 hours, after which it was allowed to cool to room temperature. Ethyl acetate was added to dilute the mixture and the GC internal standard (dodecane) was added. An aliquot was passed through Celite into a GC phial and the clear solution was subjected to GC analysis.

4-11 Control experiment (7) – Transfer hydrogenation from *N*,*N*'-diamide derived from aliphatic aldehyde

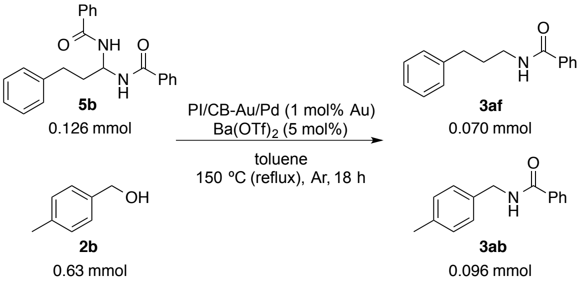

Similar procedure as elaborated in Section "4-10 Control experiment (6) – Transfer hydrogenation from the *N*,*N*'-diamide". The crude mixture obtained was concentrated and then purified using preparative TLC. However, the two products were inseparable and we determined the yields by ^{1}H NMR analysis.

Preparation of *N*,*N*'-(3-phenylpropane-1,1-diyl)dibenzamide (**5b**)

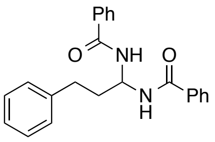

This compound was prepared using the method shown in Section 4-9 with benzamide and 3-phenylpropanal as the starting materials.
^{1}H NMR (600 MHz, DMSO-d$_6$): δ 8.61 (d, *J* = 7.5 Hz, 2H), 7.87 (d, *J* = 7.7 Hz, 4H), 7.54 (t, *J* = 7.2 Hz, 2H), 7.47 (t, *J* = 7.5 Hz, 4H), 7.29–7.25 (m, 4H), 7.18 (t, *J* = 7.0 Hz, 1H), 5.85 (quint, *J* = 7.3 Hz, 1H), 2.69 (t, *J* = 7.9 Hz, 2H), 2.17 (q, *J* = 7.8 Hz, 2H); **^{13}C NMR (150 MHz, DMSO-d$_6$):** δ 165.6, 141.4, 134.3, 131.2, 128.24, 128.20, 128.1, 127.3, 125.7, 57.0, 35.7, 31.2. **HRMS (DART):** calculated for $C_{23}H_{23}N_2O_2^+$ [*M*+H$^+$] 359.17595, found 359.17523.

N-(3-Phenylpropyl)benzamide (**3af**)[12]

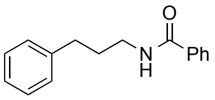

^{1}H NMR (500 MHz, CDCl$_3$): δ 7.65 (d, *J* = 7.7 Hz, 2H), 7.47–7.18 (m, 8H), 6.19 (br s, 1H), 3.48 (dt, *J* = 7.1, 7.1 Hz, 2H), 2.71 (t, *J* = 7.4 Hz, 2H), 1.95 (dq, *J* = 7.0, 7.0 Hz, 2H); **^{13}C NMR (150 MHz, CHCl$_3$):** δ 167.4, 141.4, 134.6, 131.3, 128.52, 128.46, 128.4, 126.8, 126.0, 39.8, 33.5, 31.1.

4-12 Control experiment (8) - Disproportionation reaction

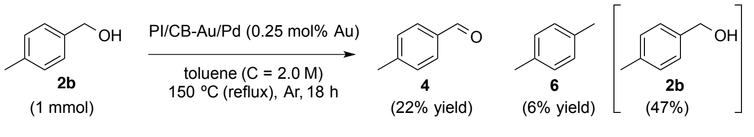

determined by GC analysis

A Carousel® tube was charged with 4-methylbenzylalcohol (**1b**, 113.9 mg, 0.93 mmol), PI/CB-Au/Pd (12.0 mg, 0.0025 mmol wrt Au) and a stirring bar. The carousel tube was then placed in an argon-filled glovebox. In the glovebox, the tube was sealed tightly with a septum. The septum was also taped tightly to the tube and the tube was then removed from the glovebox. Deoxidised toluene (0.5 mL) were added, and then the part of the septum where the syringe needle went through was covered with tape. The tube was then placed on a Carousel® (parallel synthesiser from Radleys) that had been preheated to 150 ºC and it was left to stir for 18 hours, after which it was allowed to cool to room temperature. Ethyl acetate was added to dilute the mixture and dodecane (GC internal standard) was added. An aliquot was passed through a thin pad of Celite into a GC vial for GC analysis. Yields of products were determined by GC analysis.

4-13 Control experiment (9) – Disproportionation reaction with $Ba(OTf)_2$

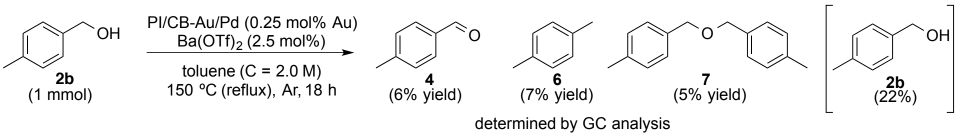

A Carousel® tube was charged with PI/CB-Au/Pd (11.8 mg, 0.0025 mmol wrt Au) and a stirring bar. The carousel tube was then placed in an argon-filled glovebox. In the glovebox, $Ba(OTf)_2$ (12.0 mg, 0.0275 mmol) was added and the tube was sealed tightly with a septum. The septum was also taped tightly to the tube and the tube was then removed from the glovebox. 4-Methylbenzyl alcohol (125.3 mg, 1.03 mmol) and deoxidized toluene (0.5 mL) were added, and then the part of the septum where the syringe needle went through was covered with tape. The tube was then placed on a Carousel® (parallel synthesiser from Radleys) that had been preheated to 150 ºC and it was left to stir for 18 hours, after which it was allowed to cool to room temperature. Ethyl acetate was added to dilute the mixture and the GC internal standard (dodecane) was added. An aliquot was passed through Celite into a GC phial and the clear solution was subjected to GC analysis.

4-14 Control experiment (10) – Hydrogenation of aldehyde

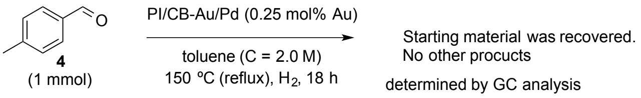

A Carousel® tube was charged with 4-methylbenzaldehyde (123.8 mg, 1.03 mmol), PI/CB-Au/Pd (11.6 mg, 0.0025 mmol wrt Au) and a stirring bar. Deoxidised toluene (0.5 mL) was added, and a three-way cock was attached to the tube. A balloon filled with H_2 was attached and the tube was flushed with hydrogen gas 10 times. The tube was then placed on a Carousel® (parallel synthesiser from Radleys) that had been preheated to 150 ºC and it was left to stir for 18 hours, after which it was allowed to cool to room temperature. Ethyl acetate was added to dilute the mixture and dodecane (GC internal standard) was added. An aliquot was passed through a thin pad of Celite into a GC vial for GC analysis. Yields of products were determined by GC analysis.

4-15 Control experiment (11) – Hydrogenation of alcohol

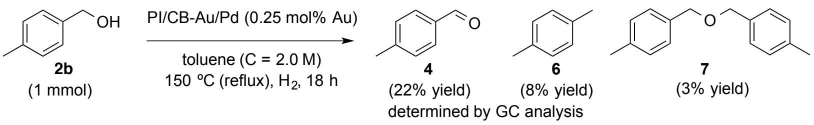

A Carousel® tube was charged with 4-methylbenzaldehyde (118.4 mg, 0.97 mmol), PI/CB-Au/Pd (11.9 mg, 0.0025 mmol wrt Au) and a stirring bar. Deoxidised toluene (0.5 mL) was added, and a three-way cock was attached to the tube. A balloon filled with H_2 was attached and the tube was flushed with hydrogen gas 10 times. The tube was then placed on a Carousel® (parallel synthesiser from Radleys) that had been preheated to 150 ºC and it was left to stir for 18 hours, after which it was allowed to cool to room temperature. Ethyl acetate was added to dilute the mixture and dodecane (GC internal standard) was added. An aliquot was passed through a thin pad of Celite into a GC vial for GC analysis. Yields of products were determined by GC analysis.

4-16 Control experiment (12) – Hydrogenation of ether

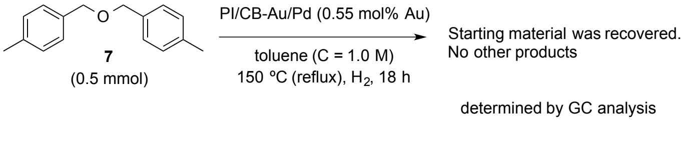

A Carousel® tube was charged with 4,4'-(oxybis(methylene))bis(methylbenzene) (116.1 mg, 0.51 mmol), PI/CB-Au/Pd (11.2 mg, 0.0025 mmol wrt Au) and a stirring bar. Deoxidised toluene (0.5 mL) was added, and a three-way cock was attached to the tube. A balloon filled with H_2 was attached and the tube was flushed with hydrogen gas 10 times. The tube was then placed on a Carousel® (parallel synthesiser from Radleys) that had been preheated to 150 ºC and it was left to stir for 18 hours, after which it was allowed to cool to room temperature. Ethyl acetate was added to dilute the mixture and dodecane (GC internal standard) was added. An aliquot was passed through a thin pad of Celite into a GC vial for GC analysis. Yields of products were determined by GC analysis.

4-17 Control experiment (13) – Transfer hydrogenation of ether

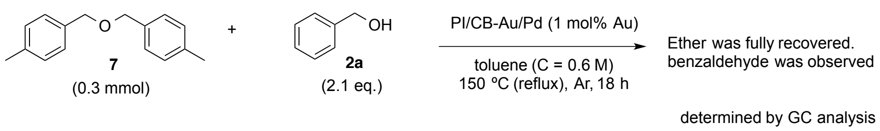

A Carousel® tube was charged with 4,4'-(oxybis(methylene))bis(methylbenzene) (70.3 mg, 0.31 mmol), PI/CB-Au/Pd (10.3 mg, 0.0025 mmol wrt Au) and a stirring bar. Deoxidised toluene (0.5 mL) was added, and a three-way cock was attached to the tube. A balloon filled with Ar was attached and the tube was flushed with argon gas 10 times. The tube was then placed on a Carousel® (parallel synthesiser from Radleys) that had been preheated to 150 ºC and it was left to stir for 18 hours, after which it was allowed to cool to room temperature. Ethyl acetate was added to dilute the mixture and dodecane (GC internal standard) was added. An aliquot was passed through a thin pad of Celite into a GC vial for GC analysis. Yields of products were determined by GC analysis.

4-18 Procedure for profiling of reaction

Each data point at a particular time on the reaction profile represents the data obtained from *one independent experiment*. In other words a number of reactions were set up at the same time with exactly the same conditions. The amount of catalyst and substrate used were the same as well. Each reaction was then removed from the heating plate after the stipulated time for that particular reaction. The reaction mixture was allowed to cool to room temperature. Ethyl acetate was then added to dilute the mixture and the GC internal standard (dodecane) was added. An aliquot was passed through Celite into a GC phial and the clear solution was subjected to GC analysis.

4-19 Reaction profile without $Ba(OTf)_2$

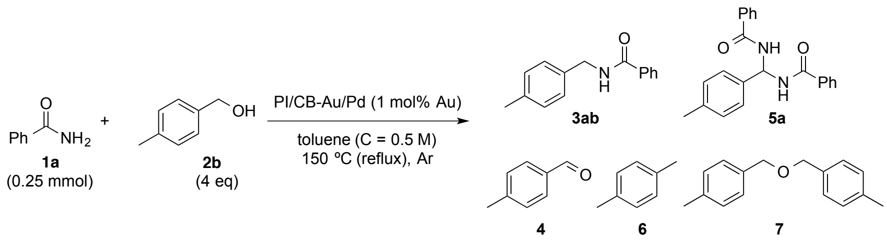

Yield was calculated based on the amount of benzamide. i.e., 0.25 mmol = 100%

time (h)	**2b** (%)	**3ab** (%)	**5a** (%)	**4** (%)	**6** (%)	**7** (%)
0.167	351	0	0	28	8	0
0.5	325	0	0	56	10	0
1	304	2	0	80	16	0
2	255	6	0.3	88	28	1
3	210	12	0.5	117	34	3
6	186	20	0.5	110	41	4

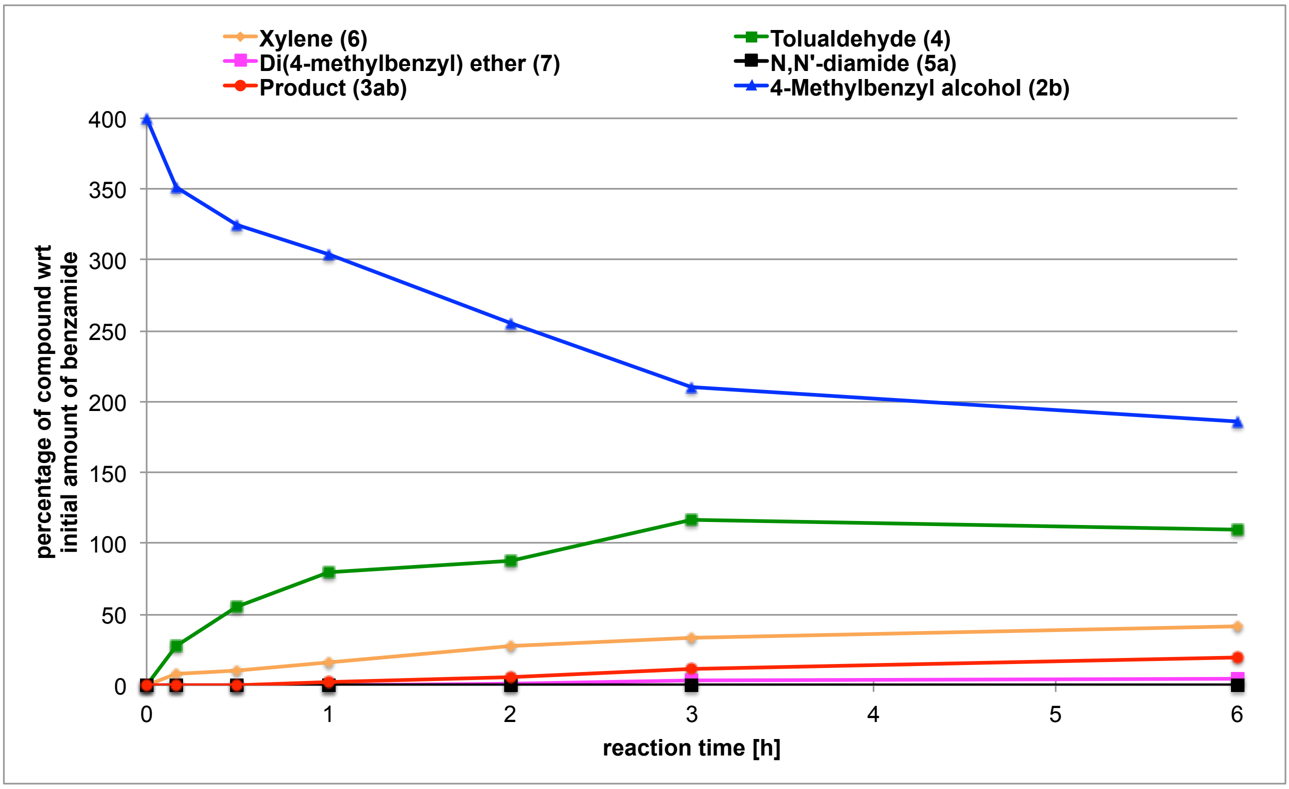

4-20 Reaction profile with $Ba(OTf)_2$

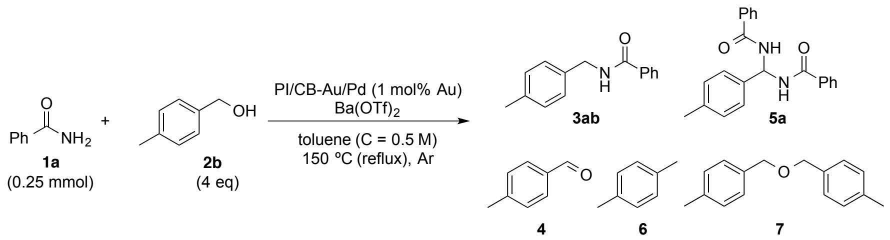

Yield was calculated based on the amount of benzamide. ie. 0.25 mmol = 100%

time (h)	**2b** (%)	**3ab** (%)	**5a** (%)	**4** (%)	**6** (%)	**7** (%)
0.167	343	4	4	18	23	2
0.5	244	23	12	38	49	7
1	166	49	13	49	63	15
2	79	72	12	53	84	25
3	45	87	3	60	87	25
6	17	96	0	50	101	27

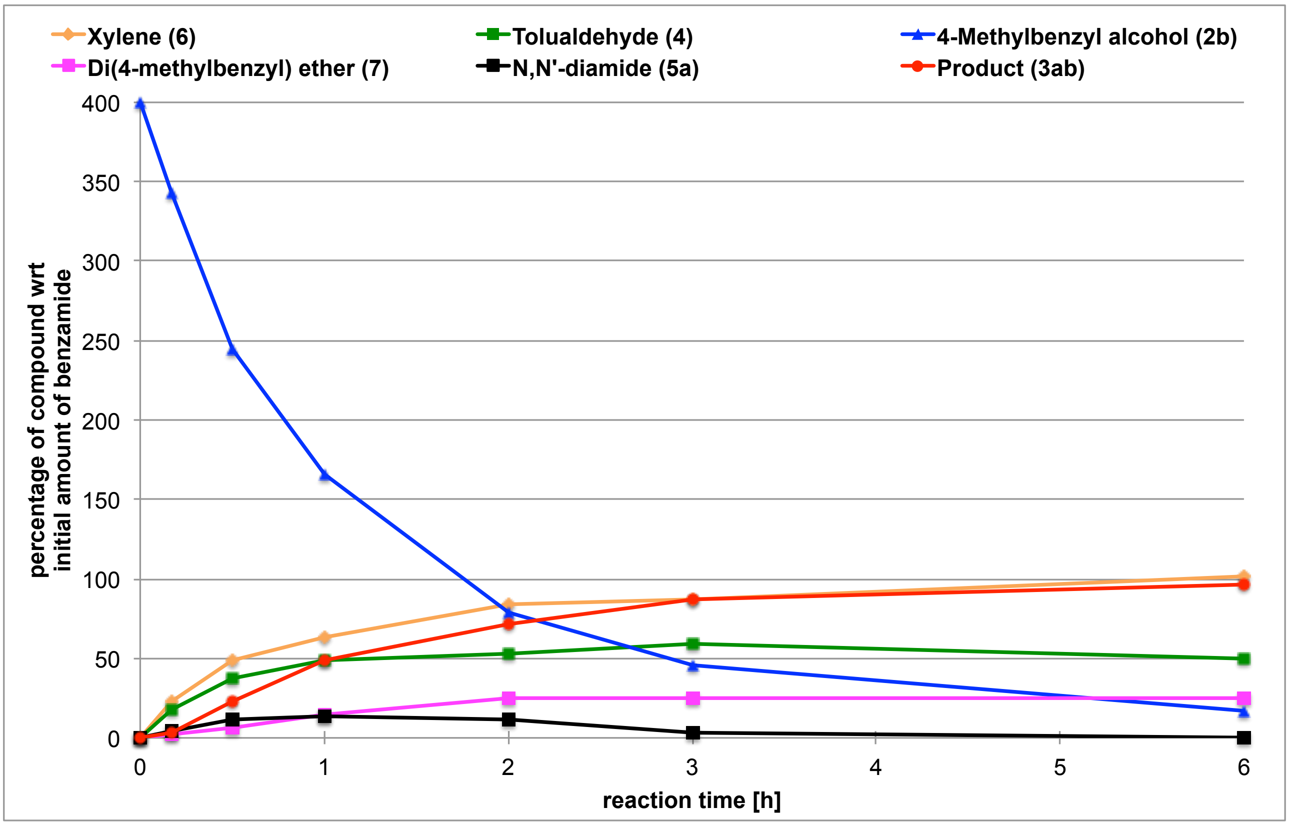

4-21 Reaction profile when $MgSO_4$ was employed with benzamide and benzyl alcohol

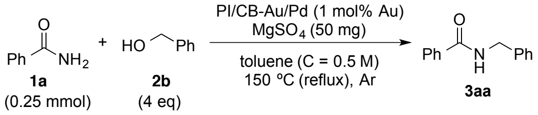

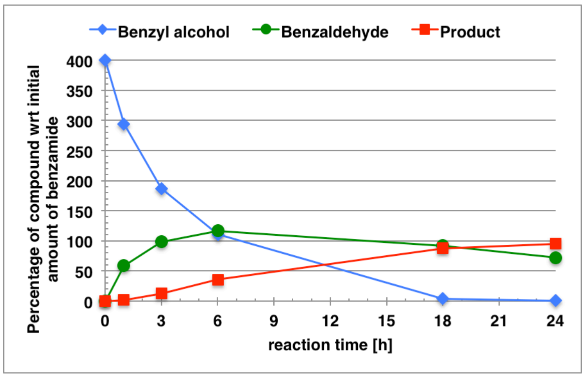

4-22 Reaction profile when $Ba(OTf)_2$ was employed with benzamide and benzyl alcohol

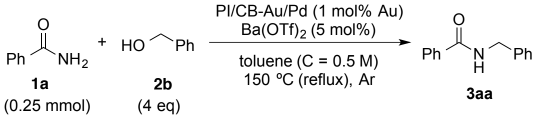

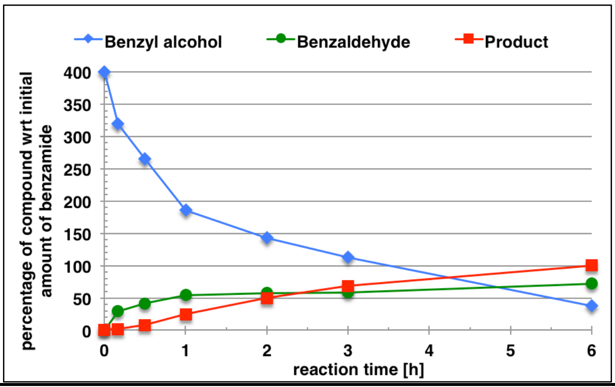

	$Ba(OTf)_2$	$MgSO_4$
time taken for consumption of half of benzyl alcohol	1 h	3 h
time taken to reach steady state for benzaldehyde	1 h	3 h
amount of benzaldehyde at steady state	0.5 eq	1 eq
time taken for reaction to reach completion	6 h	24 h
amount of alcohol left at the end of the reaction	0.5 eq	0 eq

4-23 Comparison of reaction profiles ($MgSO_4$ and $Ba(OTf)_2$)

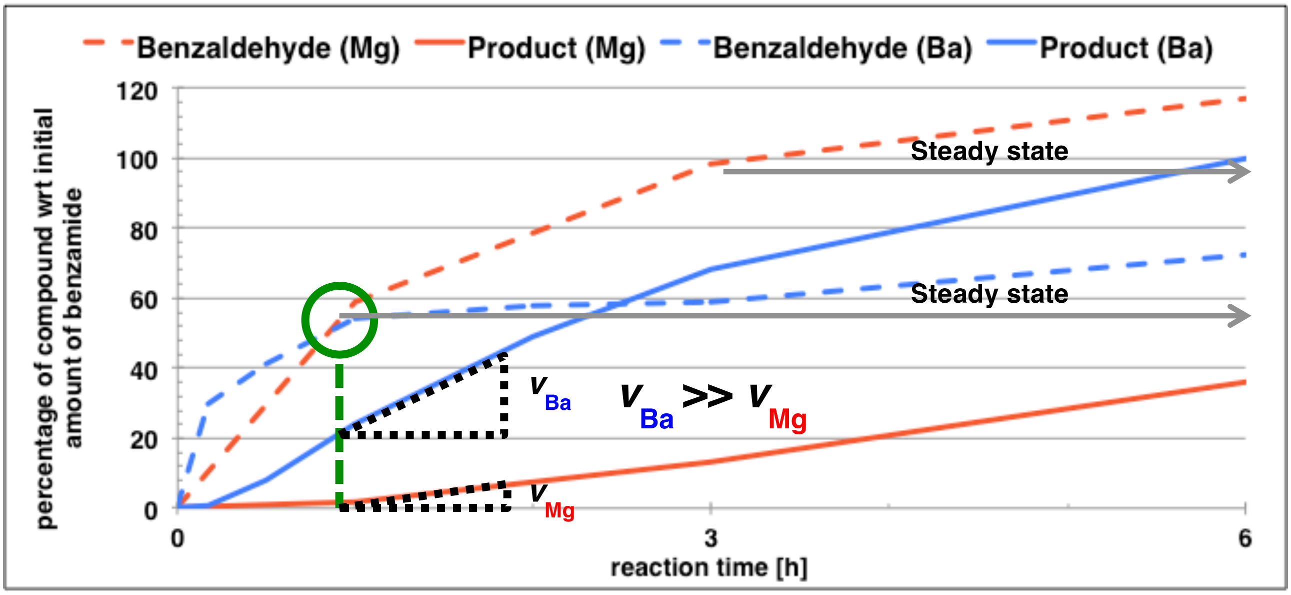

Even at the same concentration of benzaldehyde (approx. 0.55 eq, 1 h), the rate of product formation was significantly faster when $Ba(OTf)_2$ was used, as compared to when $MgSO_4$ was used.

4-24 Full consumption of alcohol when benzamide is present

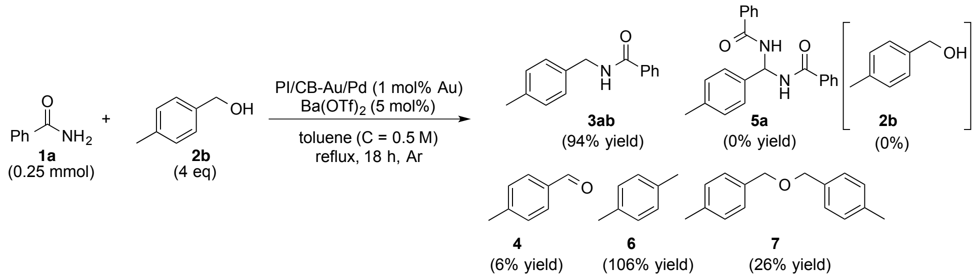

5 NMR Spectra

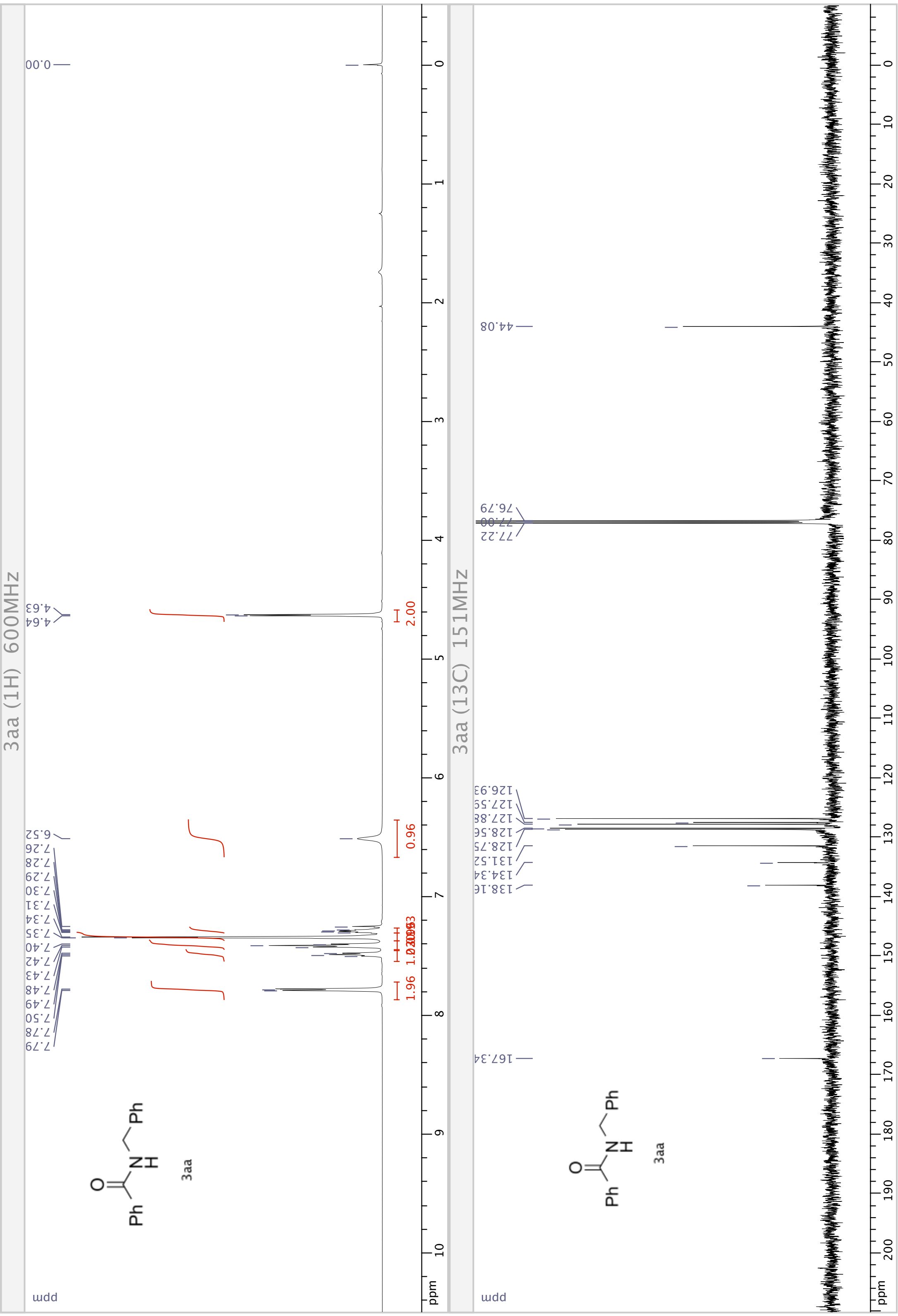

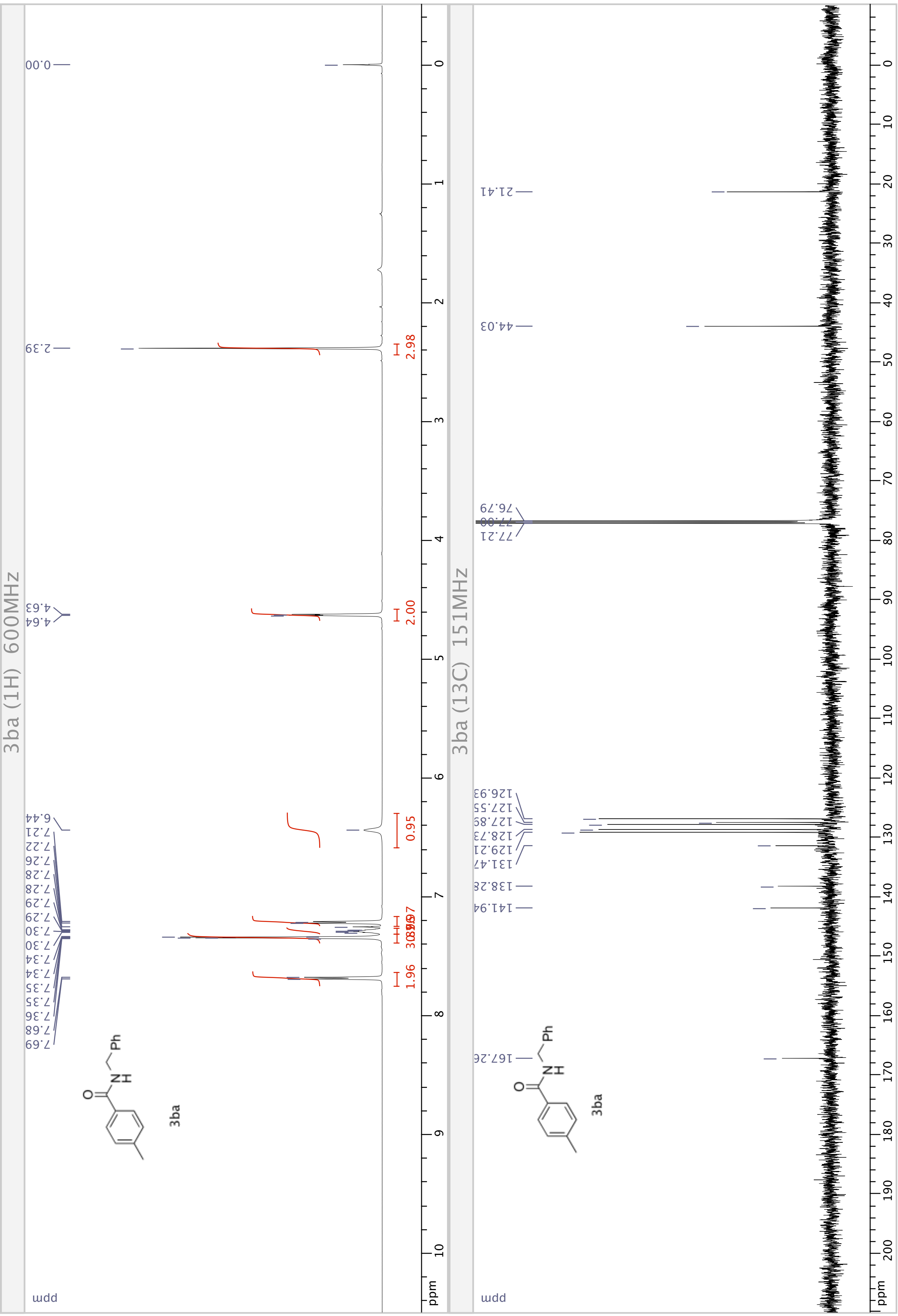
3ba (1H) 600MHz
0.00
2.39
4.63
4.64
6.44
7.21
7.22
7.26
7.28
7.28
7.29
7.29
7.30
7.30
7.34
7.34
7.35
7.35
7.36
7.68
7.69
2.98
2.00
0.95
1.96
3ba
ppm
3ba (13C) 151MHz
21.41
44.03
76.79
77.00
77.21
126.93
127.55
127.85
128.73
129.21
131.47
138.28
141.94
167.26
3ba
ppm

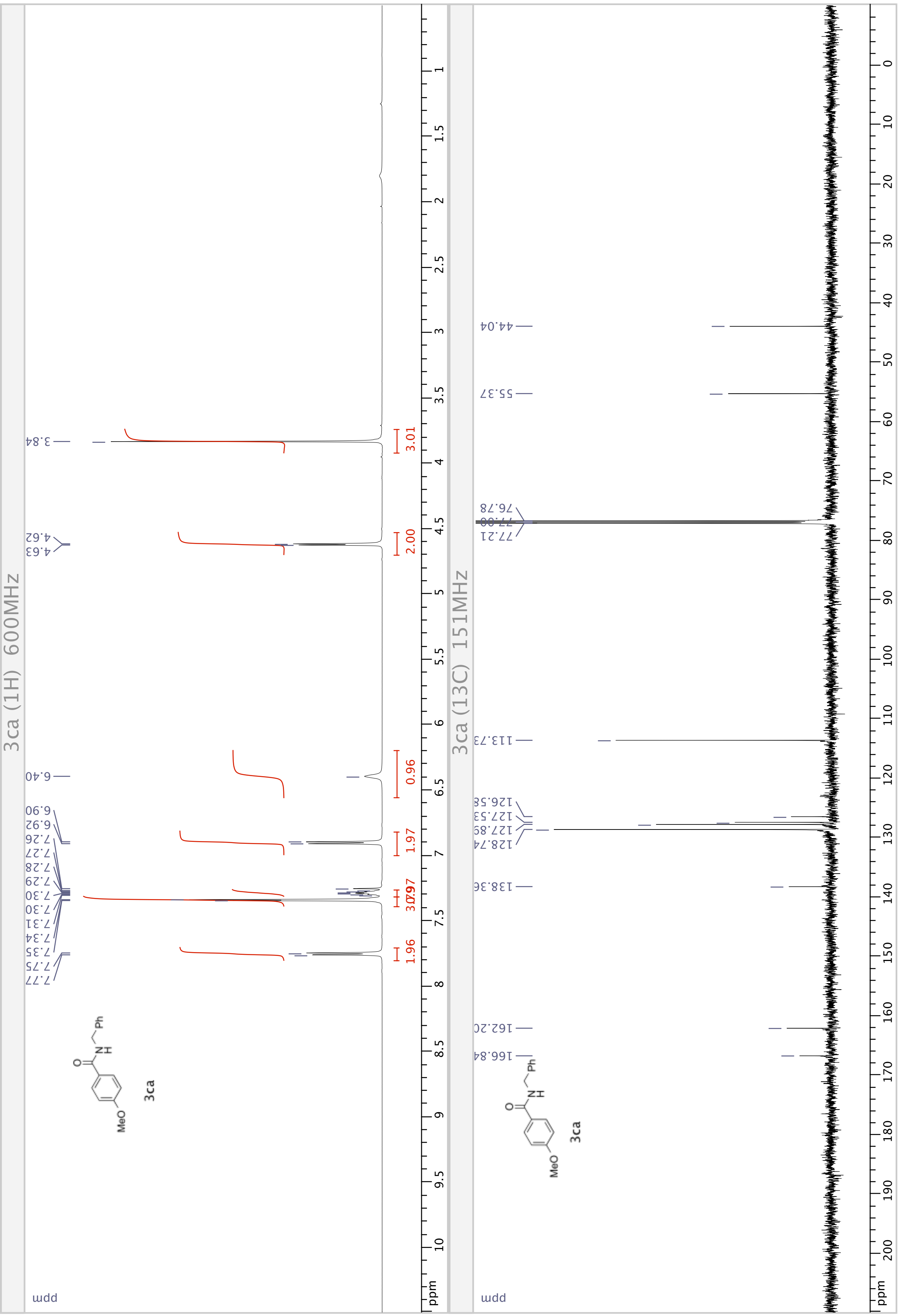
3ca (1H) 600MHz
3ca (13C) 151MHz
3ca
MeO
Ph
ppm

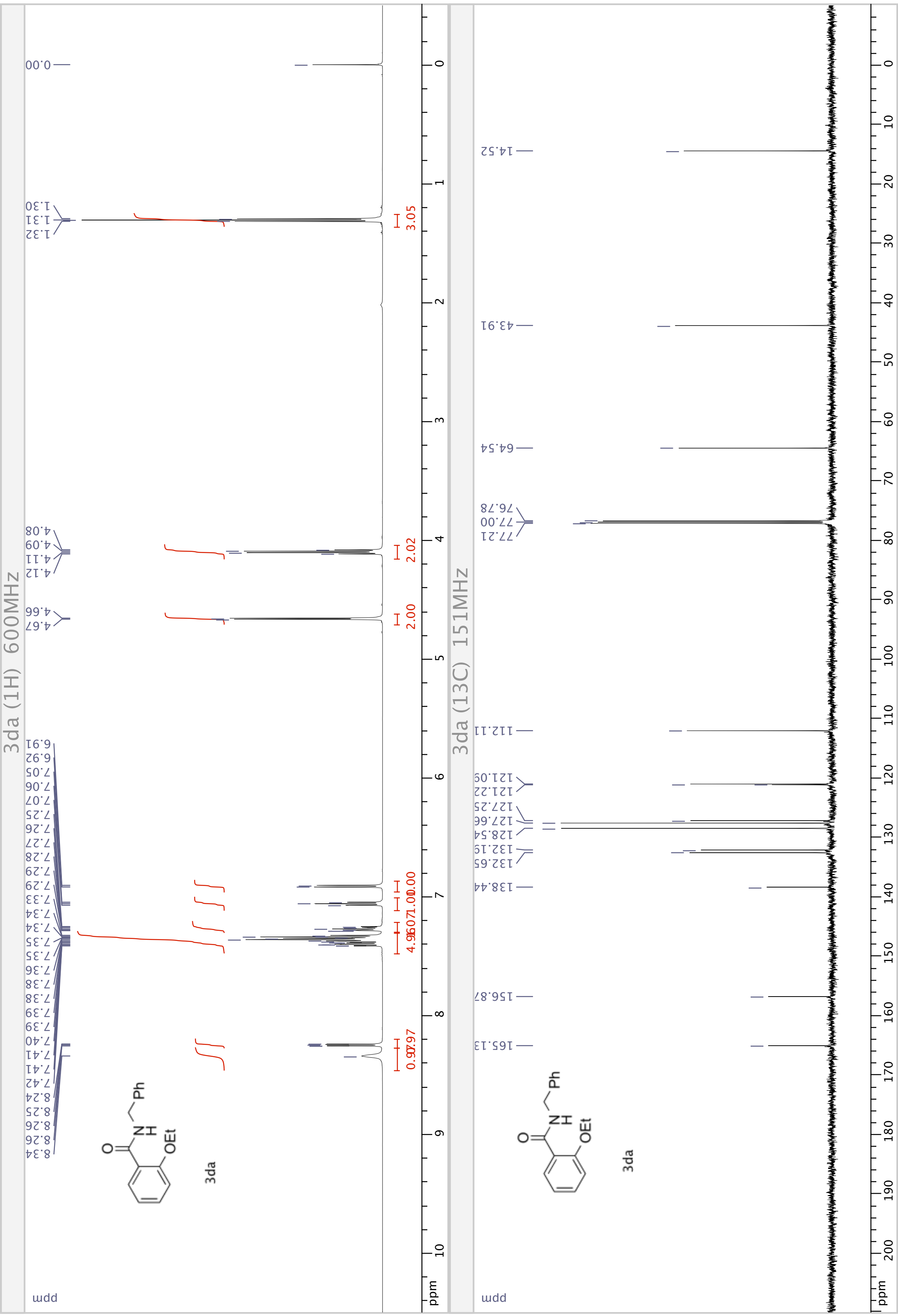
3da (1H) 600MHz
3da (13C) 151MHz
3da
3da

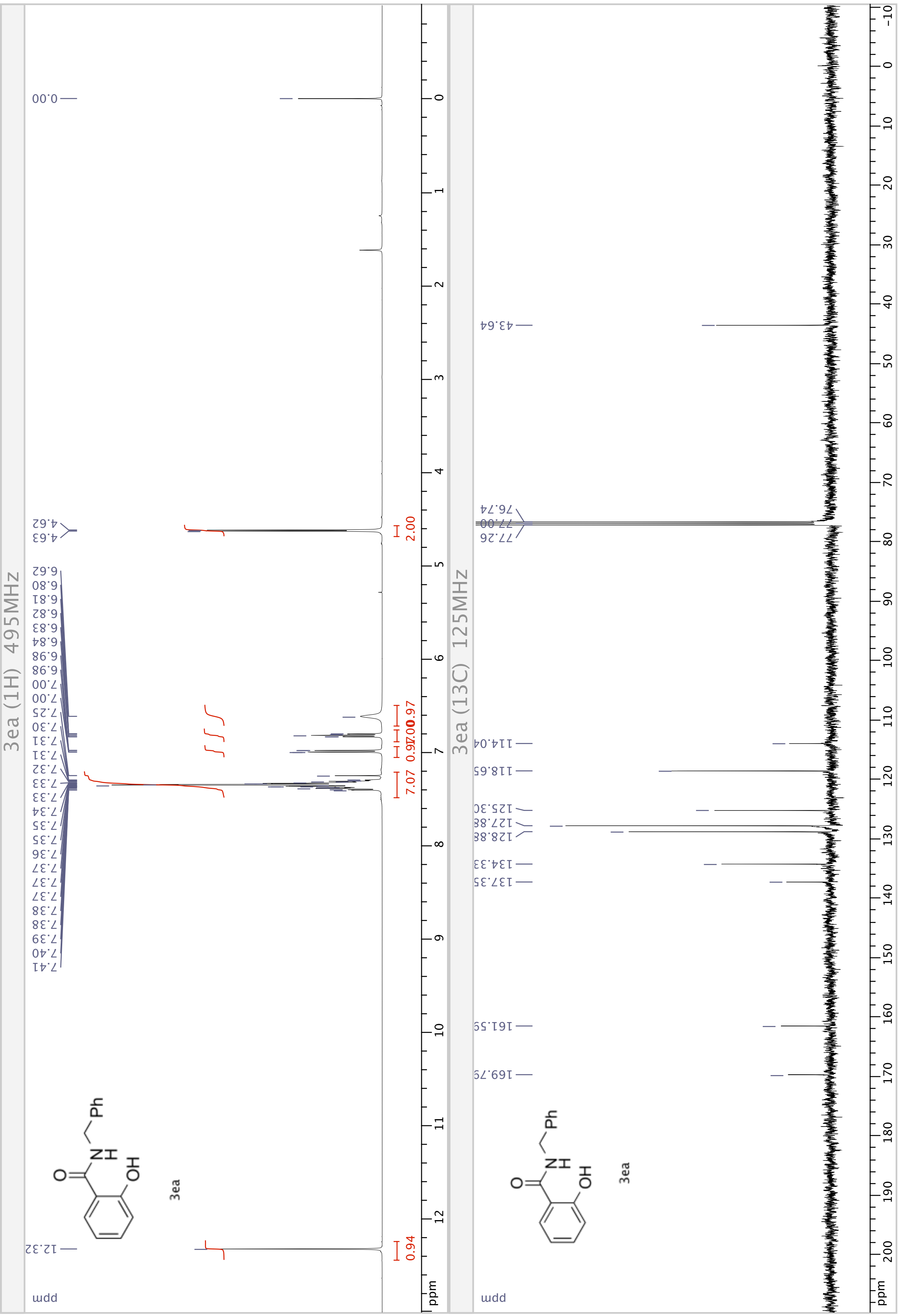
3ea (1H) 495MHz
0.00
4.62
4.63
6.62
6.80
6.81
6.82
6.83
6.84
6.98
6.98
7.00
7.00
7.25
7.30
7.31
7.31
7.32
7.33
7.33
7.34
7.35
7.35
7.36
7.37
7.37
7.37
7.38
7.38
7.39
7.40
7.41
12.32
2.00
7.07
0.94
ppm
3ea
3ea (13C) 125MHz
43.64
76.74
77.00
77.26
114.04
118.65
125.30
127.88
128.88
134.33
137.35
161.59
169.79
ppm
3ea

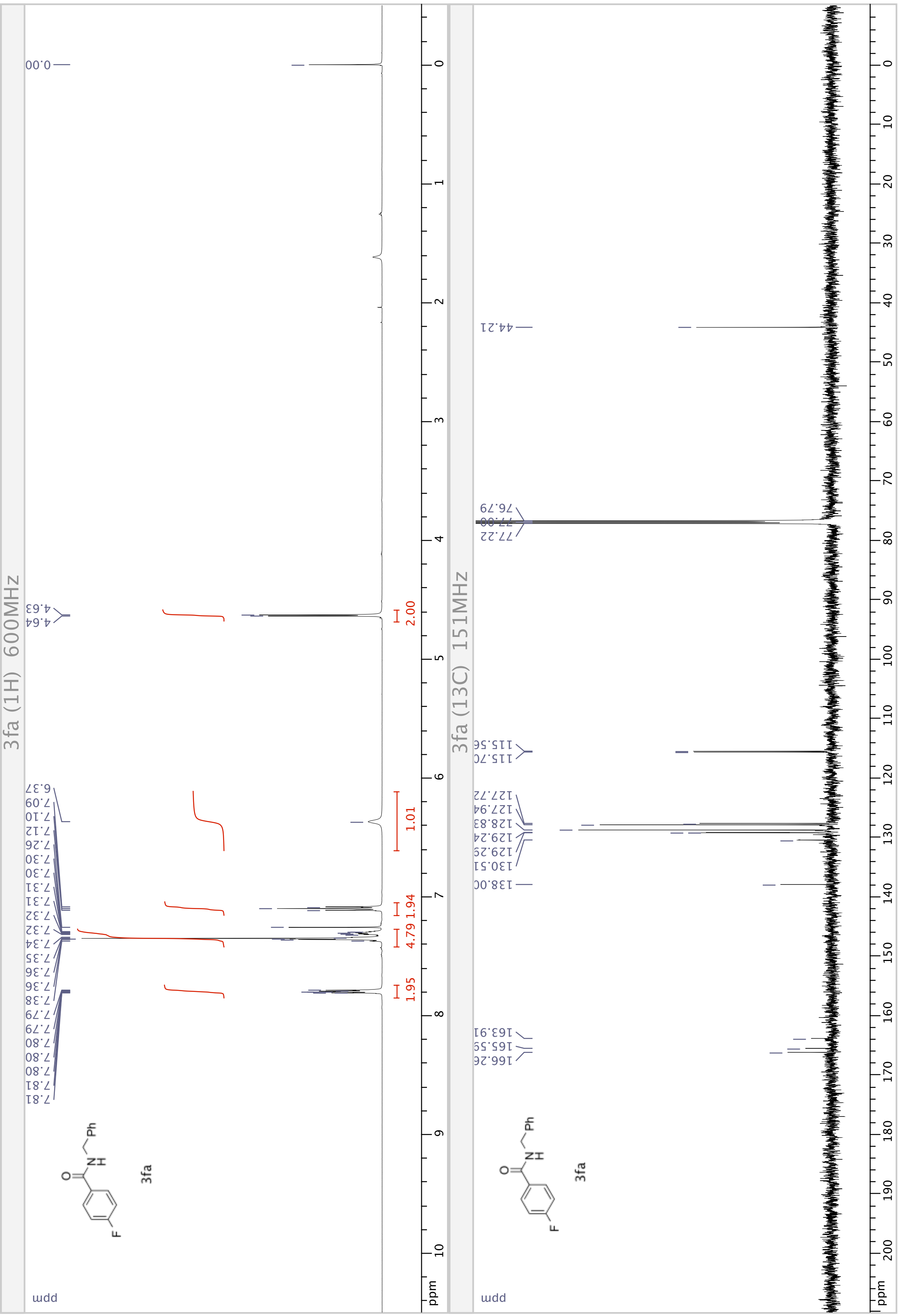
3fa (1H) 600MHz
0.00
4.63
4.64
6.37
7.09
7.10
7.12
7.26
7.30
7.30
7.31
7.31
7.32
7.32
7.34
7.35
7.36
7.36
7.38
7.79
7.79
7.80
7.80
7.80
7.81
7.81
2.00
1.01
1.94
4.79
1.95
3fa
ppm
3fa (13C) 151MHz
44.21
76.79
77.00
77.22
115.56
115.70
127.72
127.94
128.83
129.24
129.29
130.51
138.00
163.91
165.55
166.26
3fa
ppm

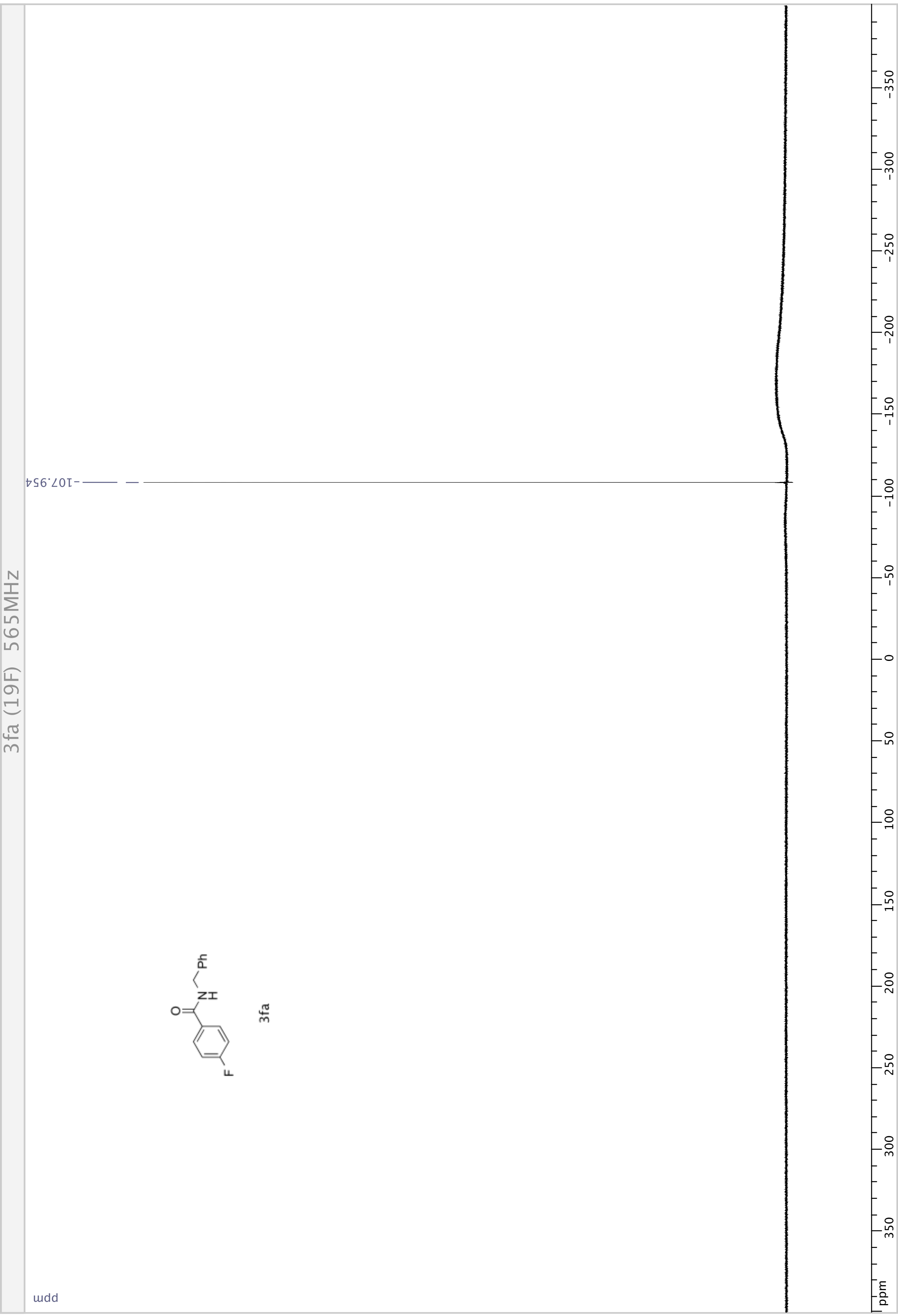
3fa (19F) 565MHz
-107.954
3fa
ppm

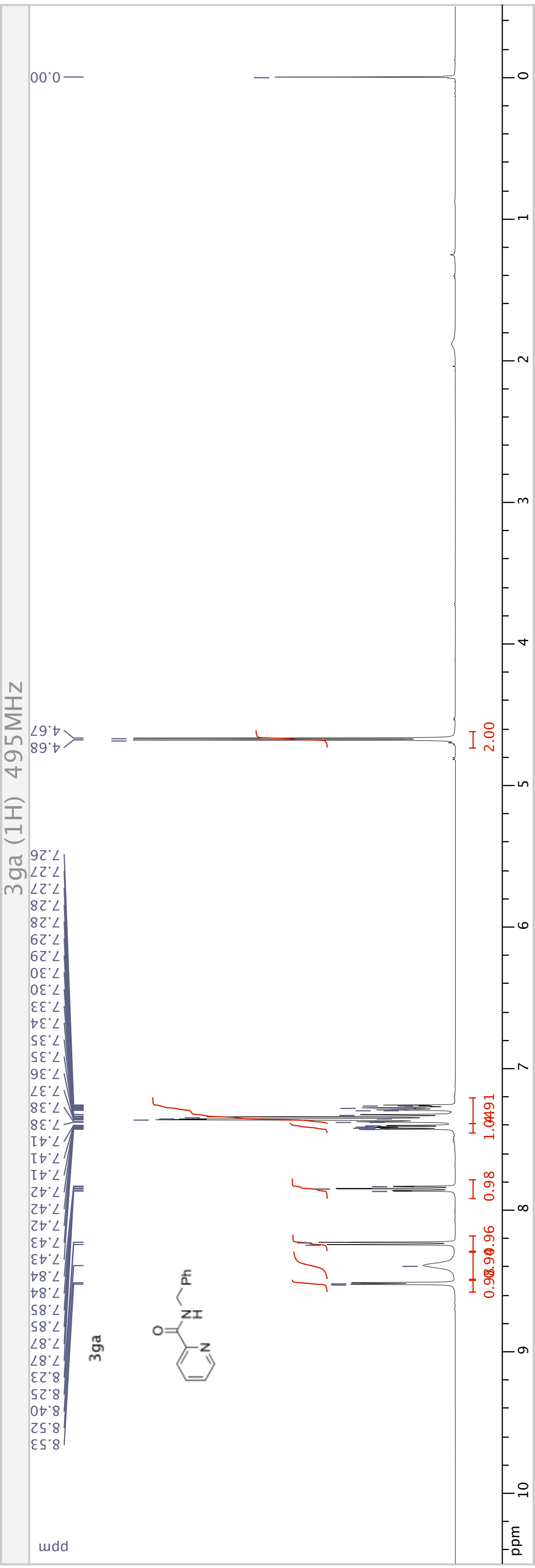
3ga (1H) 495MHz
3ga
2.00
0.98
ppm

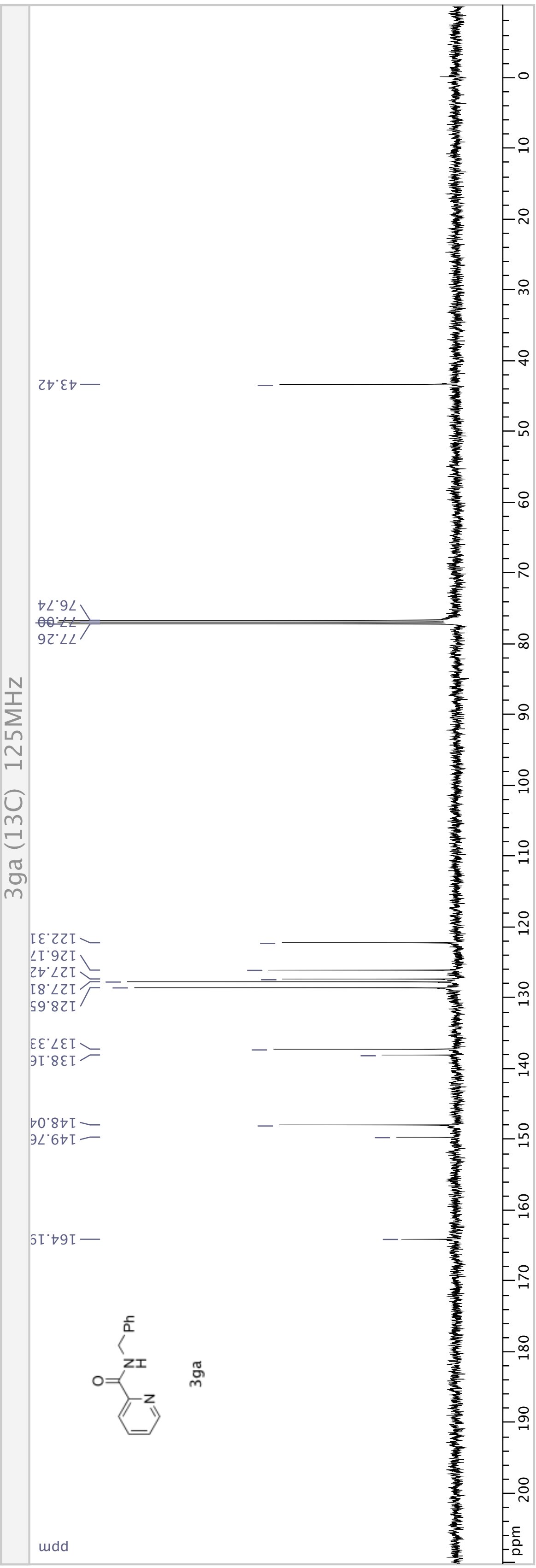
3ga (13C) 125MHz
3ga
43.42
76.74
77.26
122.31
126.17
127.42
127.81
128.65
137.33
138.16
148.04
149.76
164.19
ppm

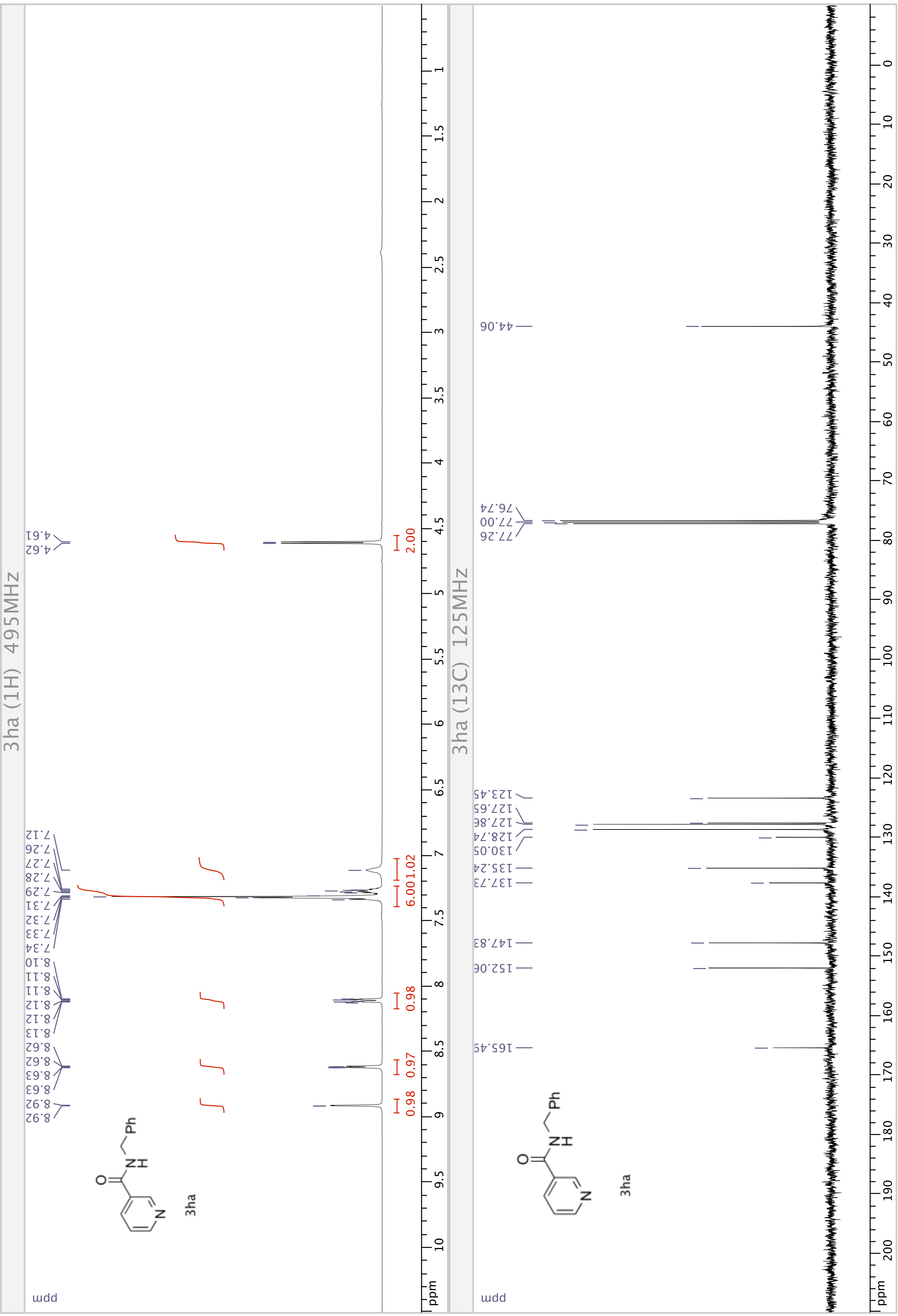
3ha (1H) 495MHz
3ha (13C) 125MHz
3ha
ppm

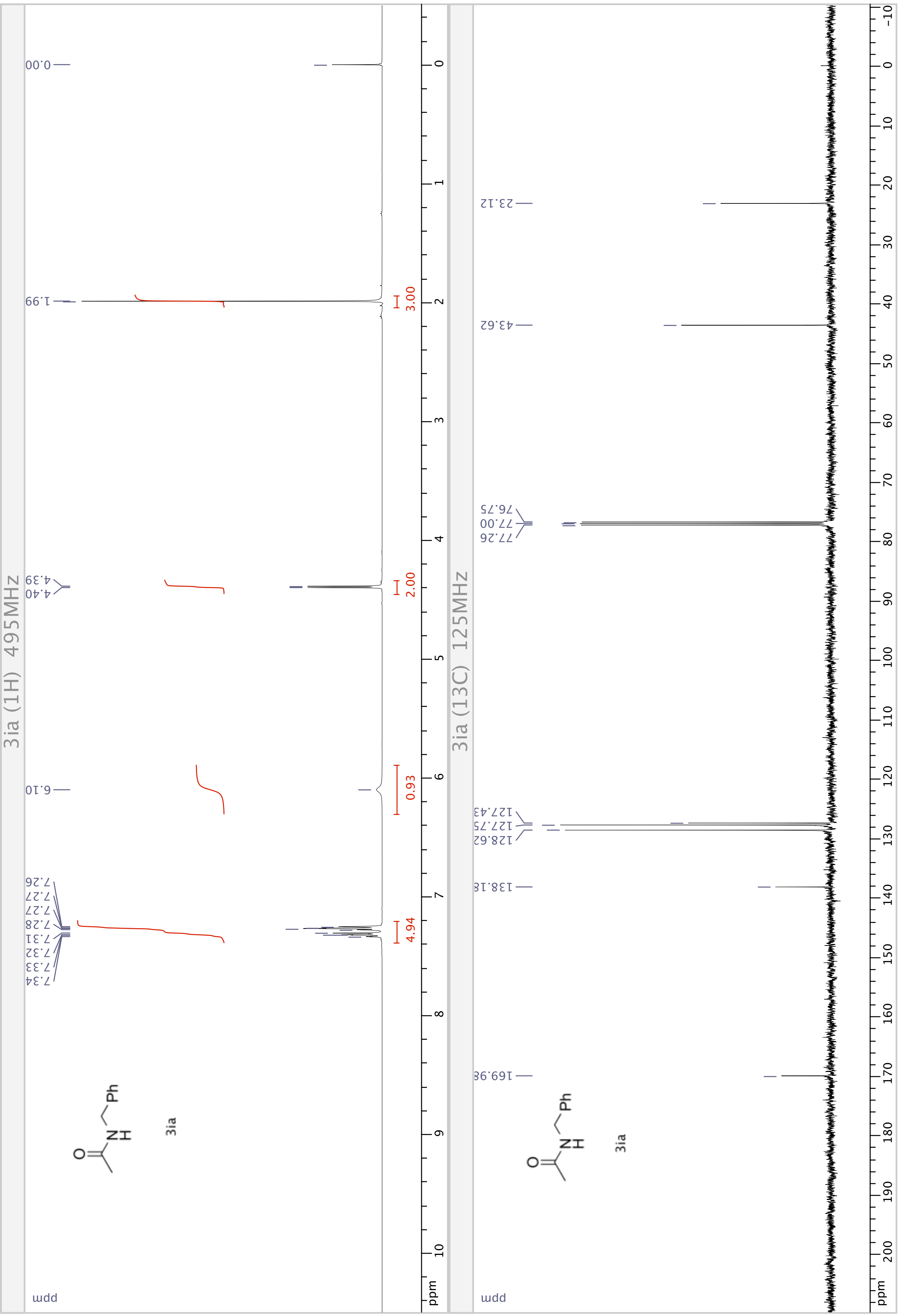
3ia (1H) 495MHz
0.00
1.99
4.39
4.40
6.10
7.26
7.27
7.27
7.28
7.31
7.32
7.33
7.34
3.00
2.00
0.93
4.94
3ia
3ia (13C) 125MHz
23.12
43.62
76.75
77.00
77.26
127.43
127.75
128.62
138.18
169.98
3ia
ppm

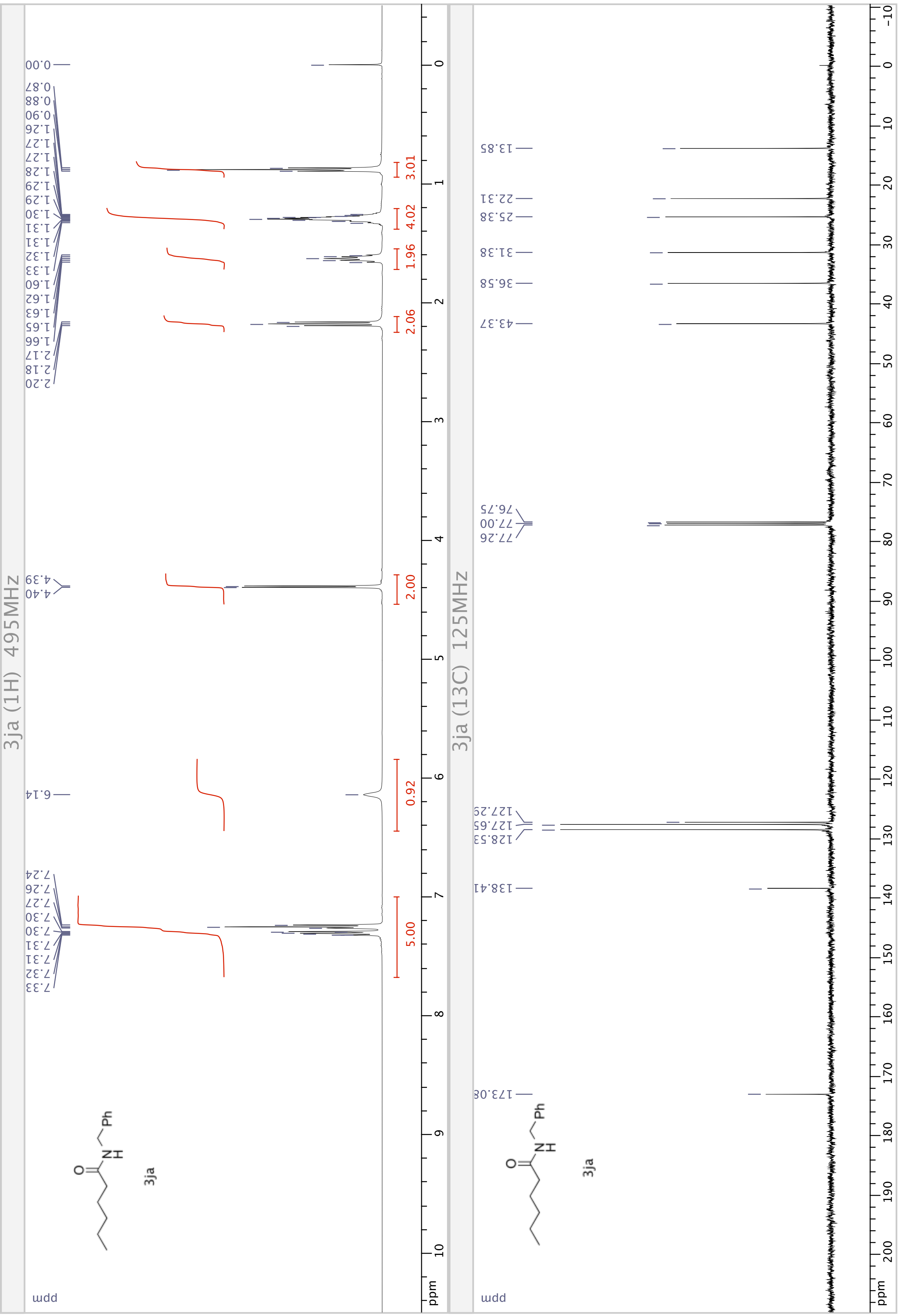
3ja (1H) 495MHz
0.00
0.87
0.88
0.90
1.26
1.27
1.27
1.28
1.29
1.29
1.30
1.31
1.31
1.32
1.33
1.60
1.62
1.63
1.65
1.66
2.17
2.18
2.20
4.39
4.40
6.14
7.24
7.26
7.27
7.30
7.30
7.31
7.31
7.32
7.33
3.01
4.02
1.96
2.06
2.00
0.92
5.00
3ja
ppm
3ja (13C) 125MHz
13.85
22.31
25.38
31.38
36.58
43.37
76.75
77.00
77.26
127.29
127.65
128.53
138.41
173.08
3ja
ppm

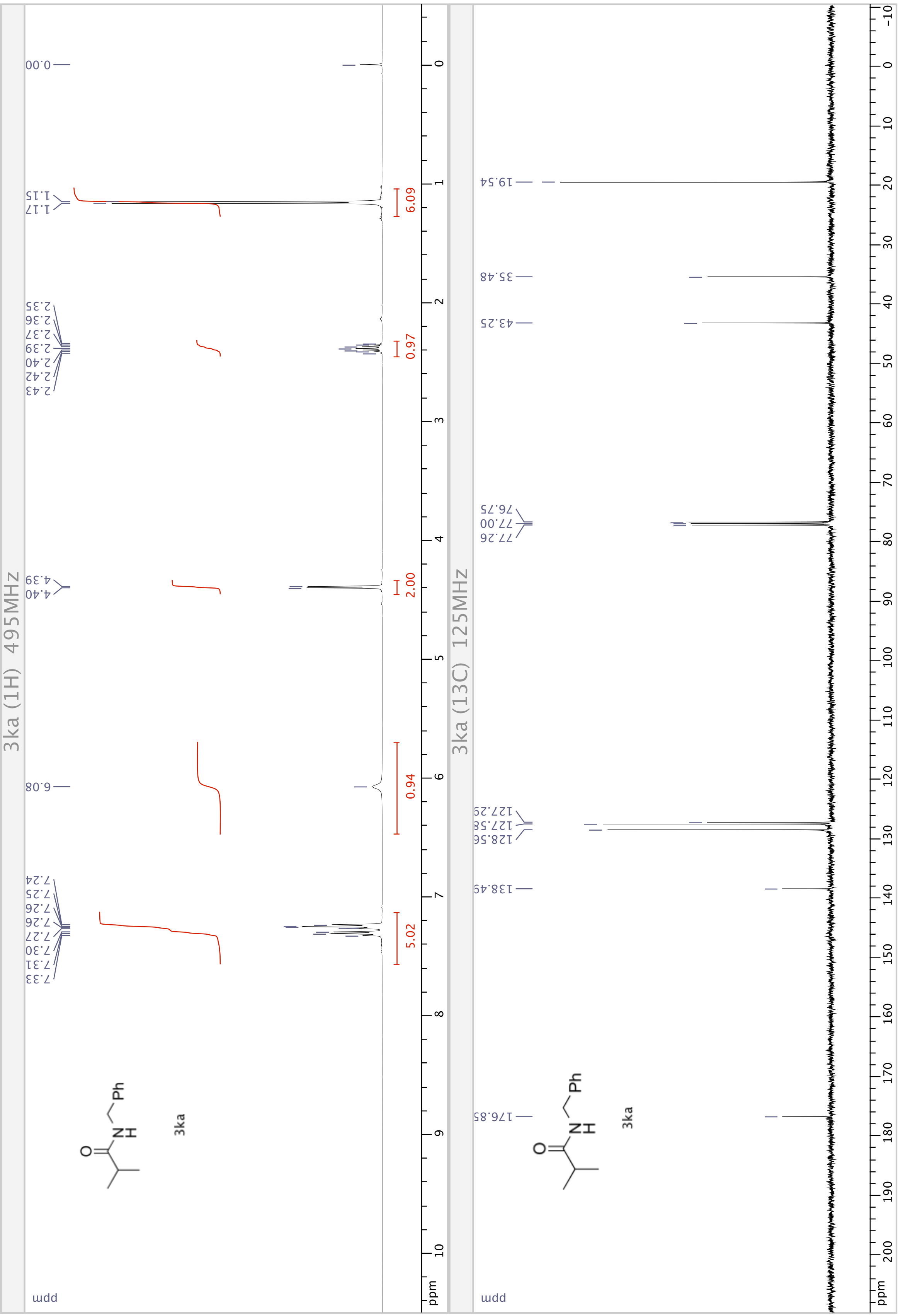
3ka (1H) 495MHz
0.00
1.15
1.17
2.35
2.36
2.37
2.39
2.40
2.42
2.43
4.39
4.40
6.08
7.24
7.25
7.26
7.26
7.27
7.30
7.31
7.33
6.09
0.97
2.00
0.94
5.02
3ka
ppm
3ka (13C) 125MHz
19.54
35.48
43.25
76.75
77.00
77.26
127.29
127.58
128.56
138.49
176.85
3ka
ppm

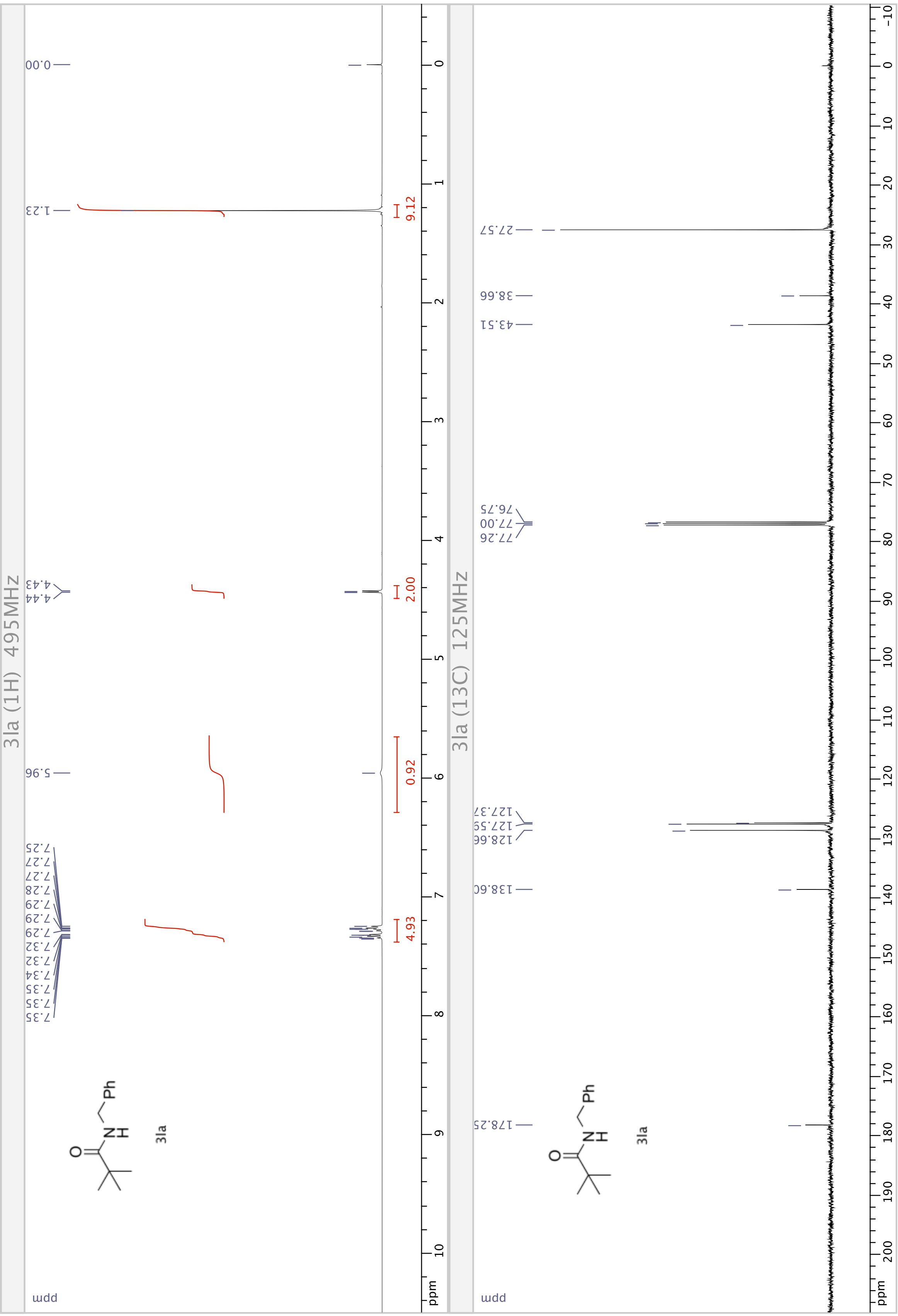
3la (1H) 495MHz
0.00
1.23
4.43
4.44
5.96
7.25
7.27
7.27
7.28
7.29
7.29
7.29
7.32
7.32
7.34
7.35
7.35
7.35
9.12
2.00
0.92
4.93
3la
ppm
3la (13C) 125MHz
27.57
38.66
43.51
76.75
77.00
77.26
127.37
127.59
128.66
138.60
178.25
3la
ppm

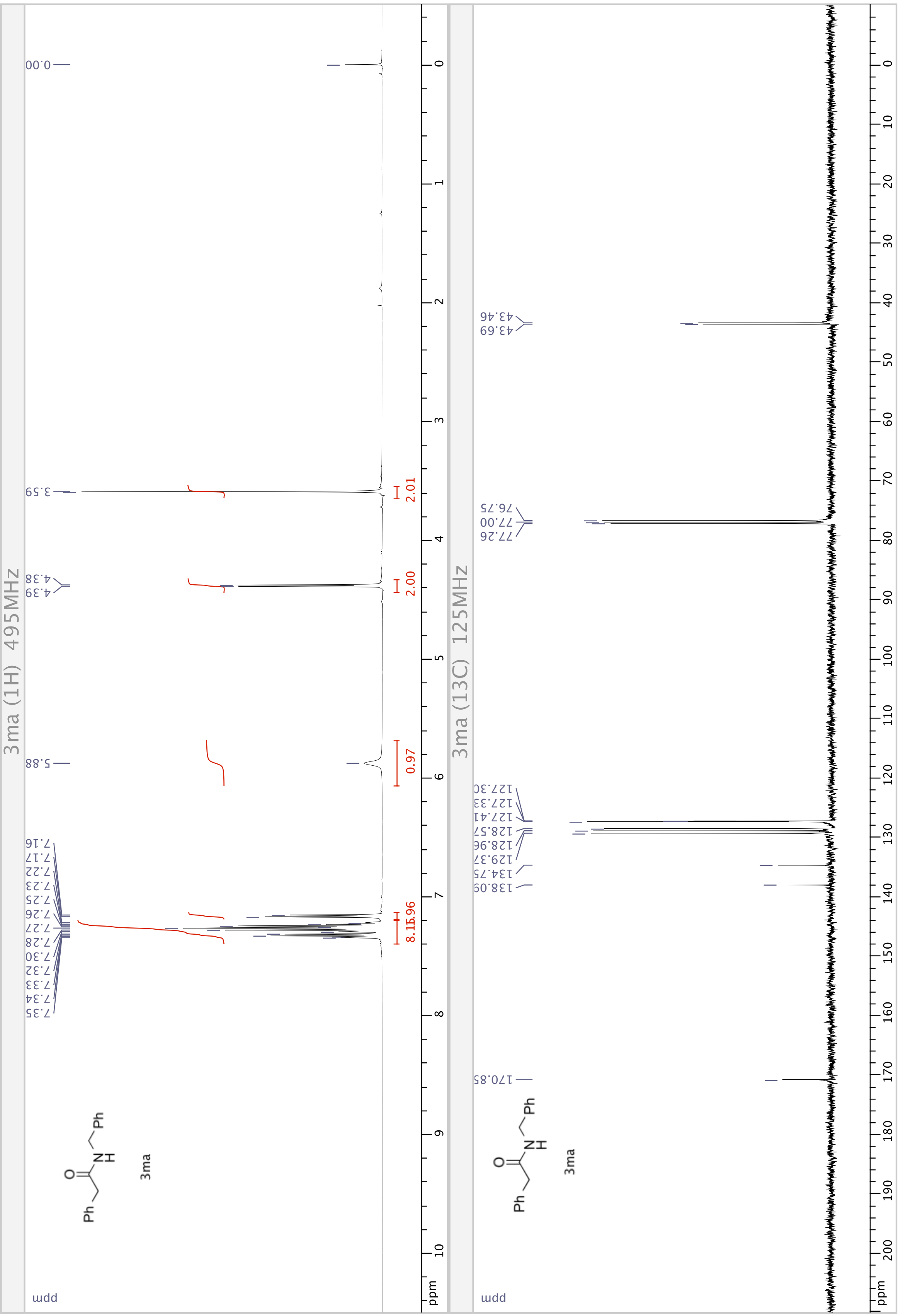
3ma (1H) 495MHz
0.00
3.59
4.38
4.39
5.88
7.16
7.17
7.22
7.23
7.25
7.26
7.27
7.28
7.30
7.32
7.33
7.34
7.35
2.01
2.00
0.97
8.15
5.96
3ma
ppm
3ma (13C) 125MHz
43.46
43.69
76.75
77.00
77.26
127.30
127.33
127.41
128.57
128.96
129.37
134.75
138.05
170.85
3ma
ppm

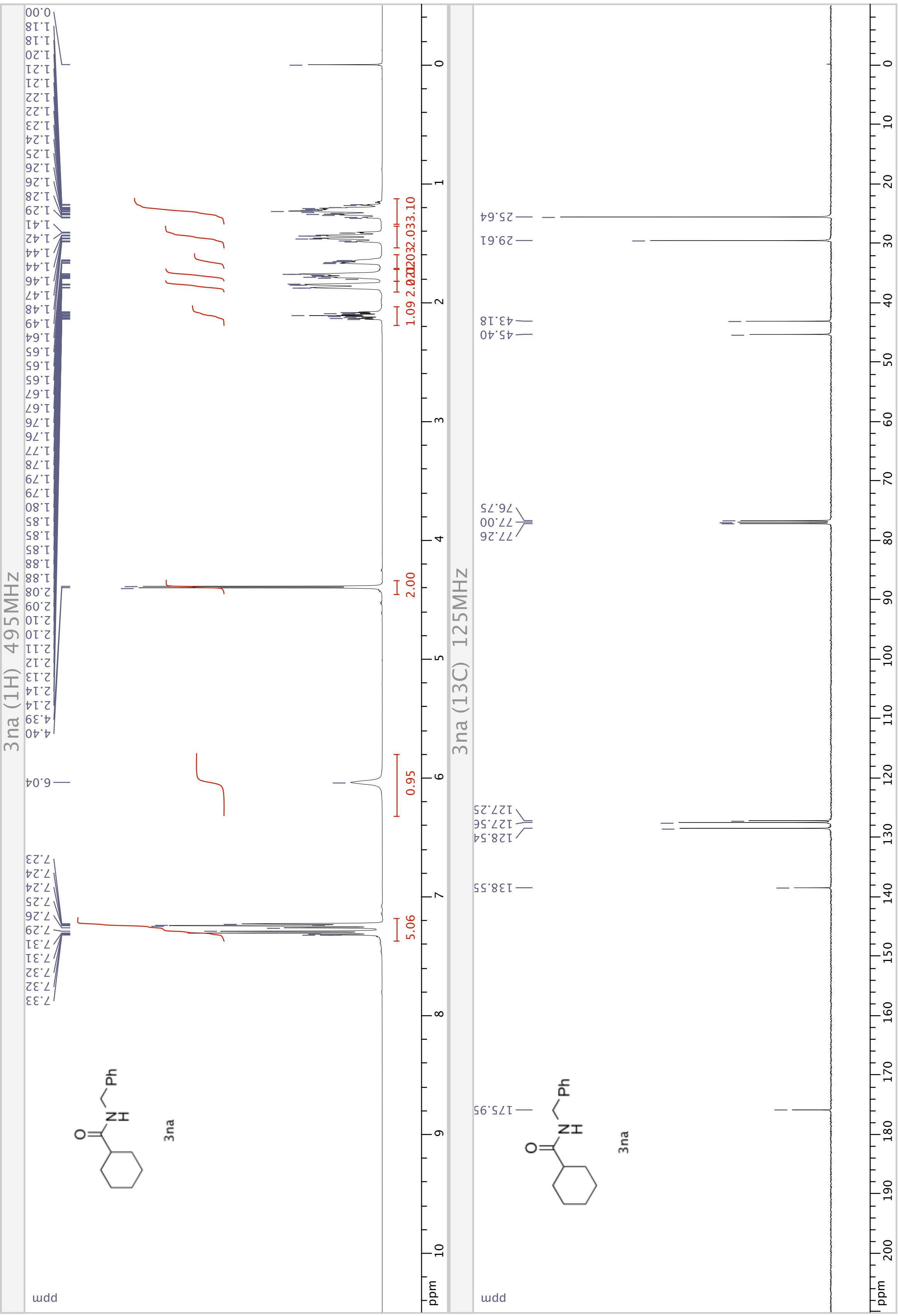
3na (1H) 495MHz
3na (13C) 125MHz
3na
ppm

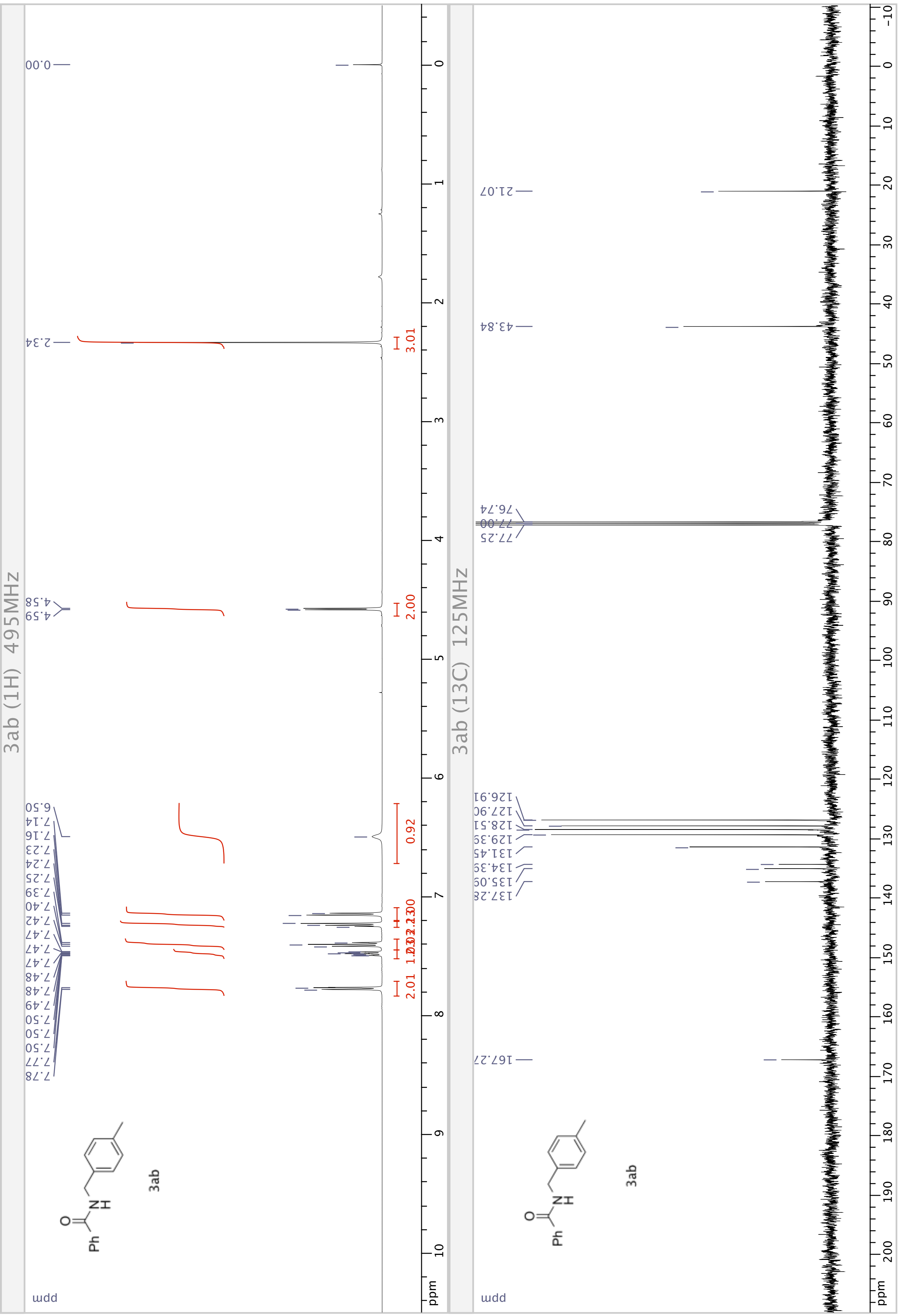
3ab (1H) 495MHz
0.00
2.34
4.58
4.59
6.50
7.14
7.16
7.23
7.24
7.25
7.39
7.40
7.42
7.47
7.47
7.47
7.48
7.48
7.49
7.50
7.50
7.50
7.77
7.78
3.01
2.00
0.92
2.01
Ph
O
N
H
3ab
ppm
3ab (13C) 125MHz
21.07
43.84
76.74
77.00
77.25
126.91
127.90
128.51
129.39
131.45
134.39
135.09
137.28
167.27

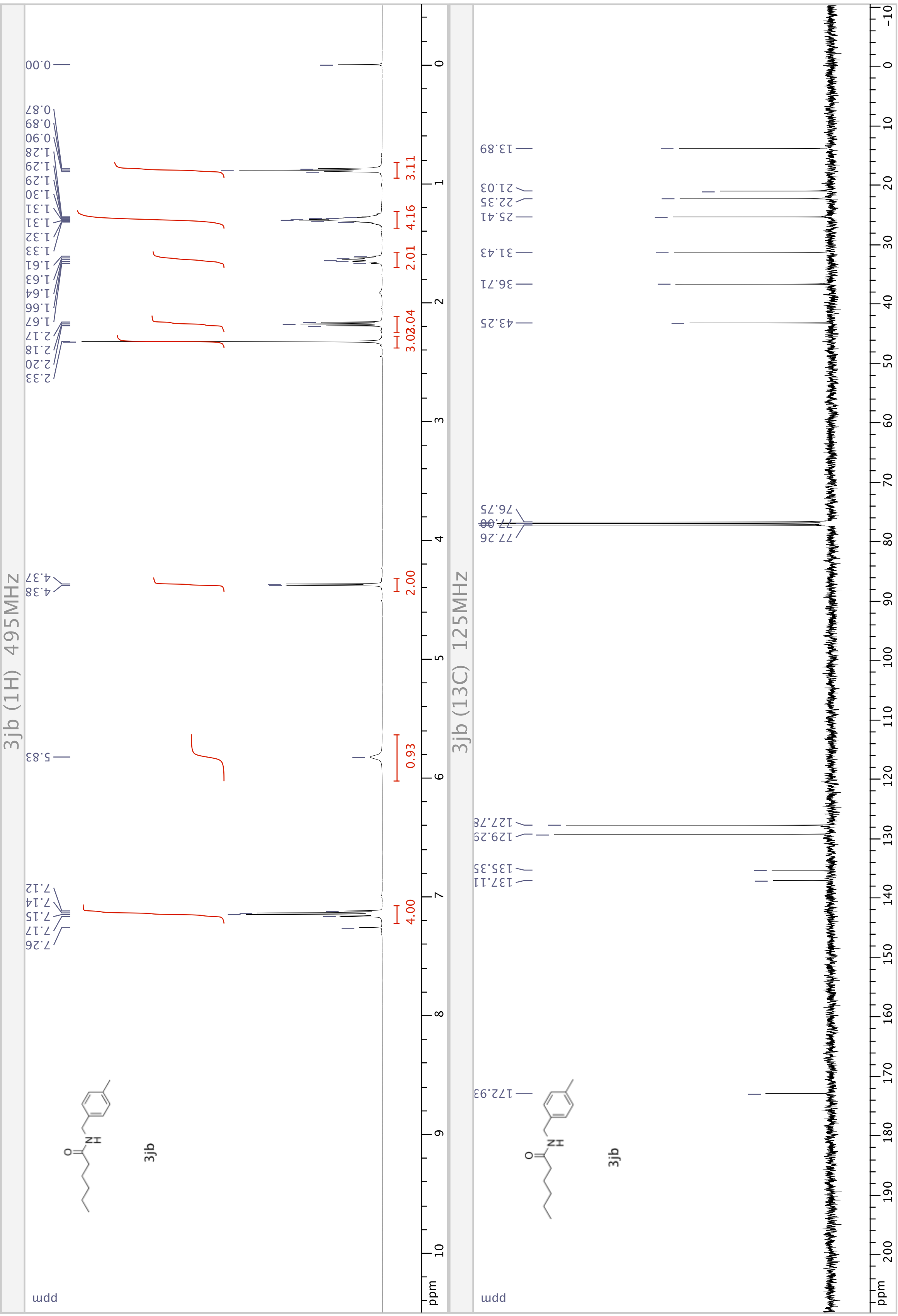
3jb (1H) 495MHz
3jb (13C) 125MHz
3jb
ppm

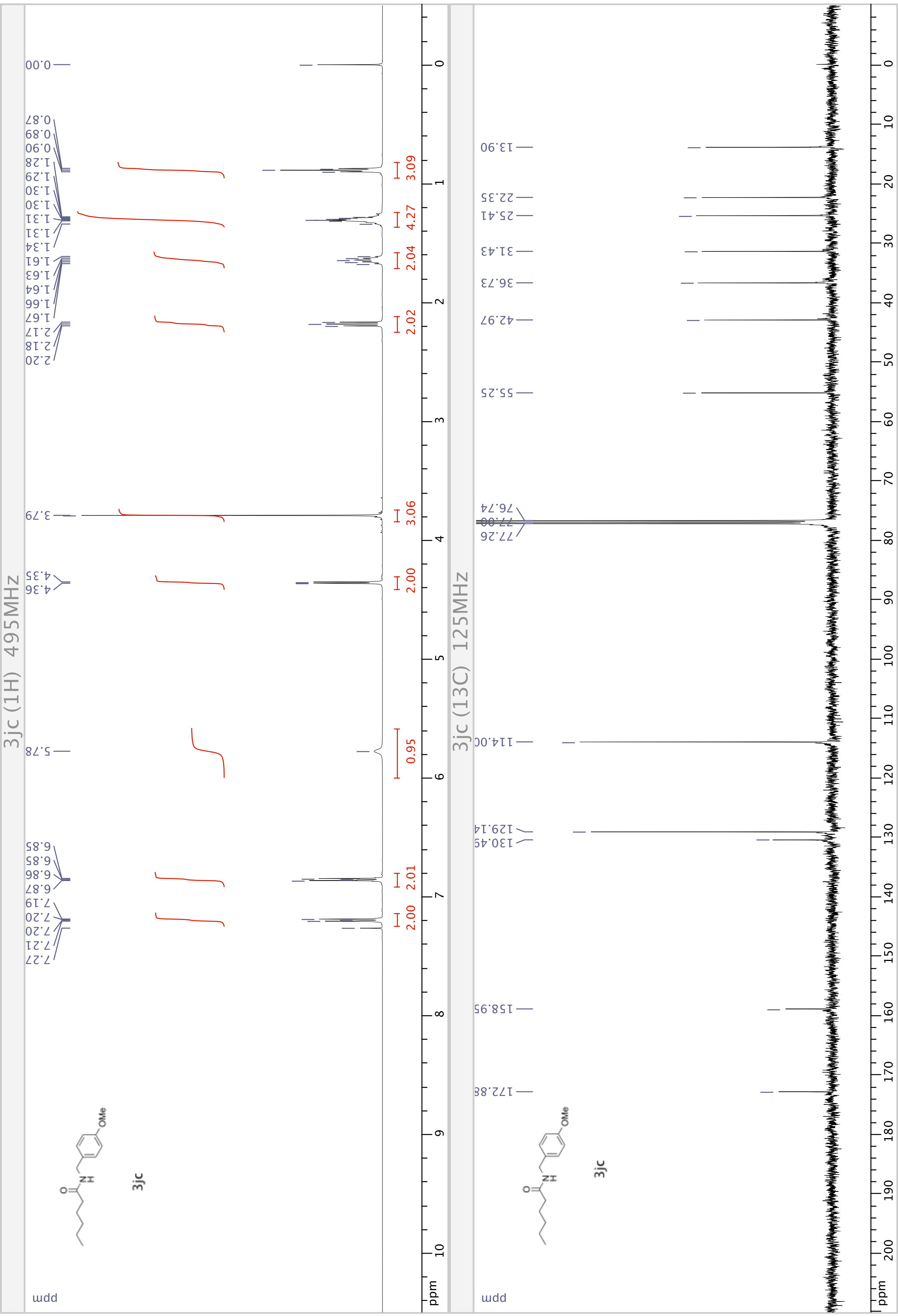
3jc (1H) 495MHz
0.00
0.87
0.89
0.90
1.28
1.29
1.30
1.30
1.31
1.31
1.34
1.61
1.63
1.64
1.66
1.67
2.17
2.18
2.20
3.79
4.35
4.36
5.78
6.85
6.85
6.86
6.87
7.19
7.20
7.20
7.21
7.27
3.09
4.27
2.04
2.02
3.06
2.00
0.95
2.01
2.00
ppm
3jc
OMe
3jc (13C) 125MHz
13.90
22.35
25.41
31.43
36.73
42.97
55.25
76.74
77.00
77.26
114.00
129.14
130.49
158.95
172.88
ppm
3jc
OMe

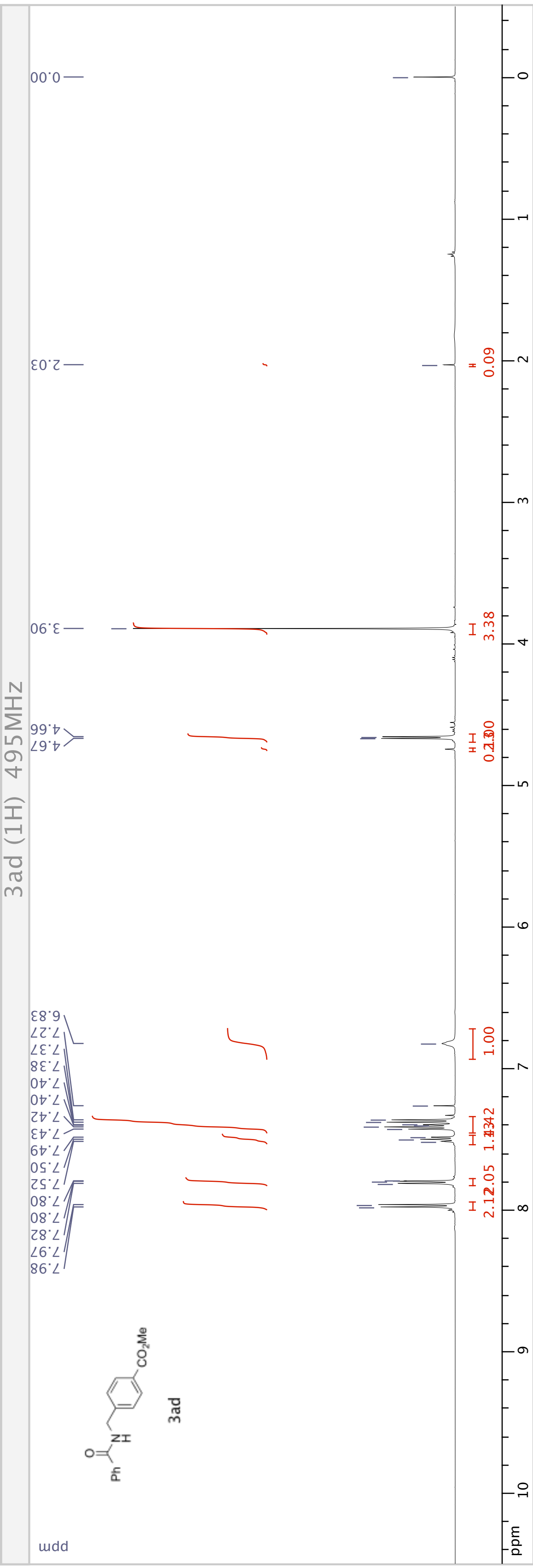
3ad (1H) 495MHz
3ad
ppm

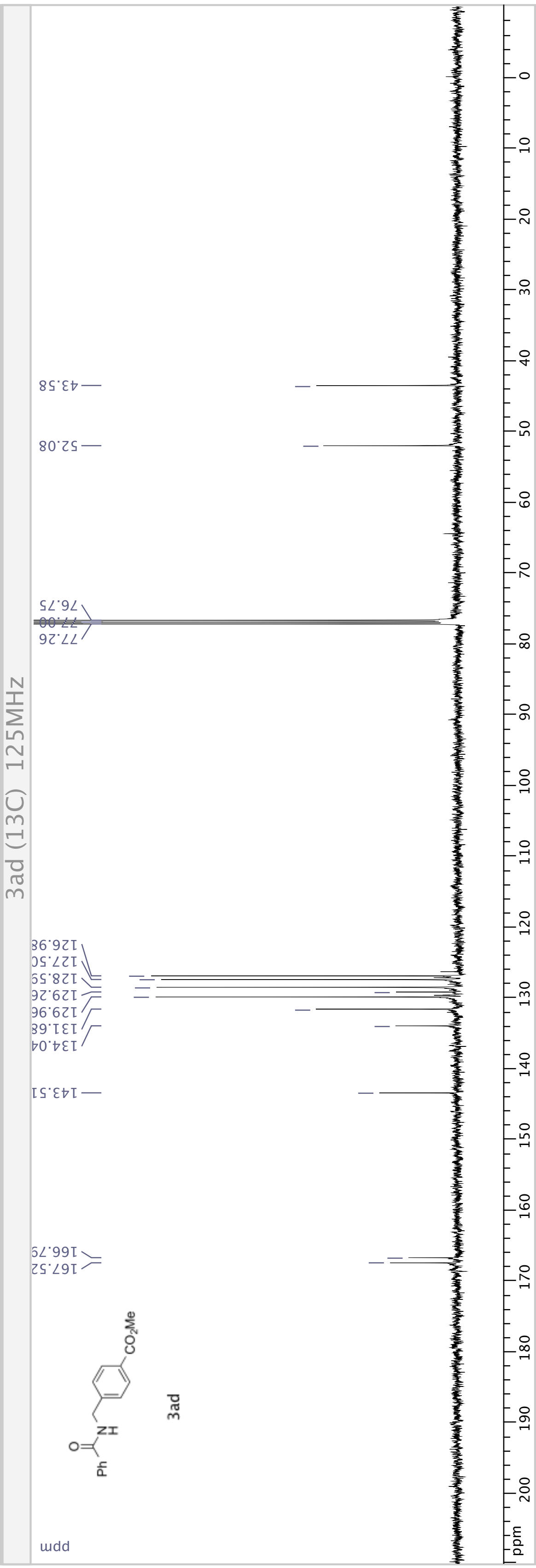
3ad (13C) 125MHz
3ad
ppm

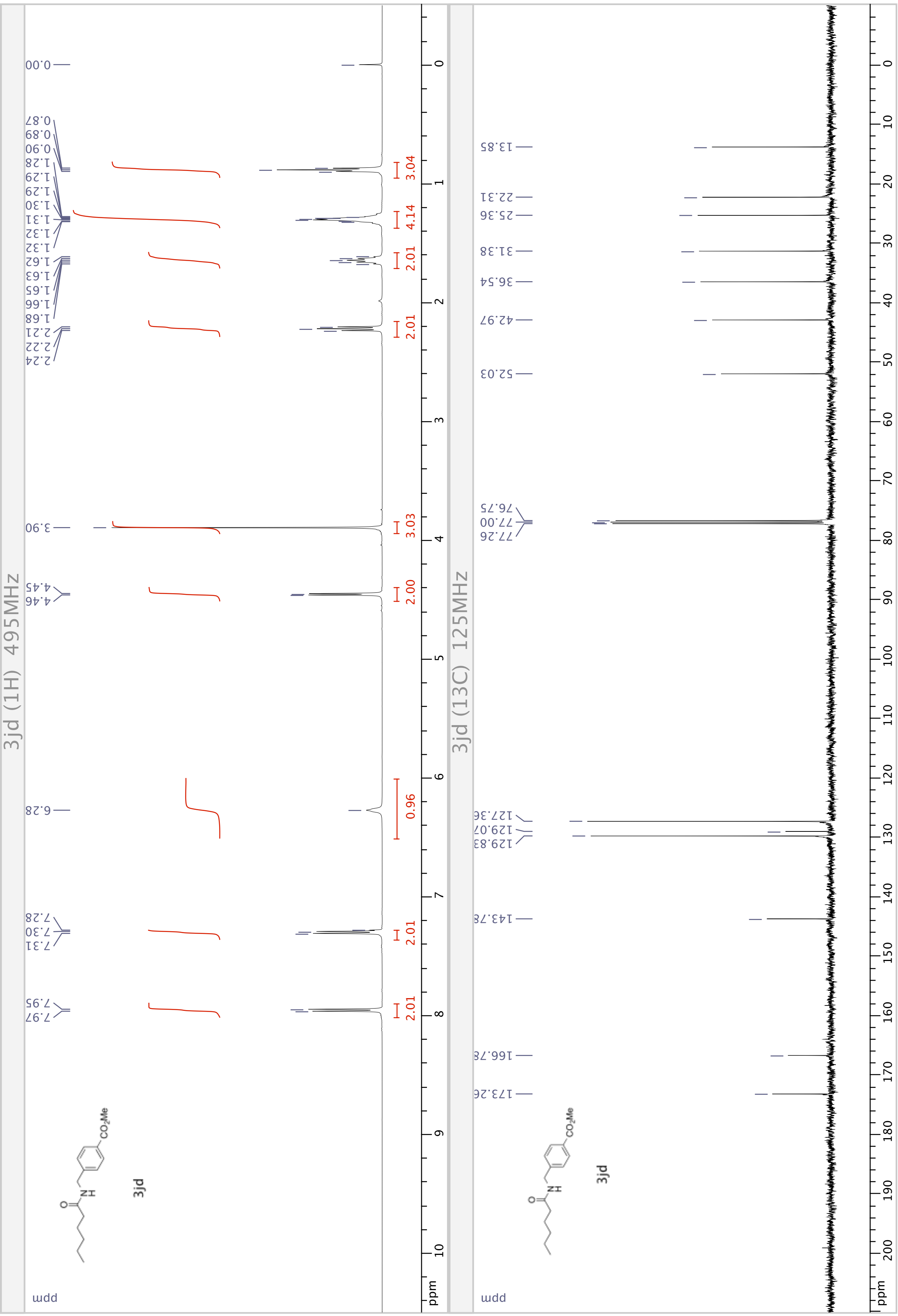
3jd (1H) 495MHz
0.00
0.87
0.89
0.90
1.28
1.29
1.29
1.30
1.31
1.32
1.32
1.62
1.63
1.65
1.66
1.68
2.21
2.22
2.24
3.90
4.45
4.46
6.28
7.28
7.30
7.31
7.95
7.97
3.04
4.14
2.01
2.01
3.03
2.00
0.96
2.01
2.01
ppm
3jd
3jd (13C) 125MHz
13.85
22.31
25.36
31.38
36.54
42.97
52.03
76.75
77.00
77.26
127.36
129.07
129.83
143.78
166.78
173.26
ppm
3jd

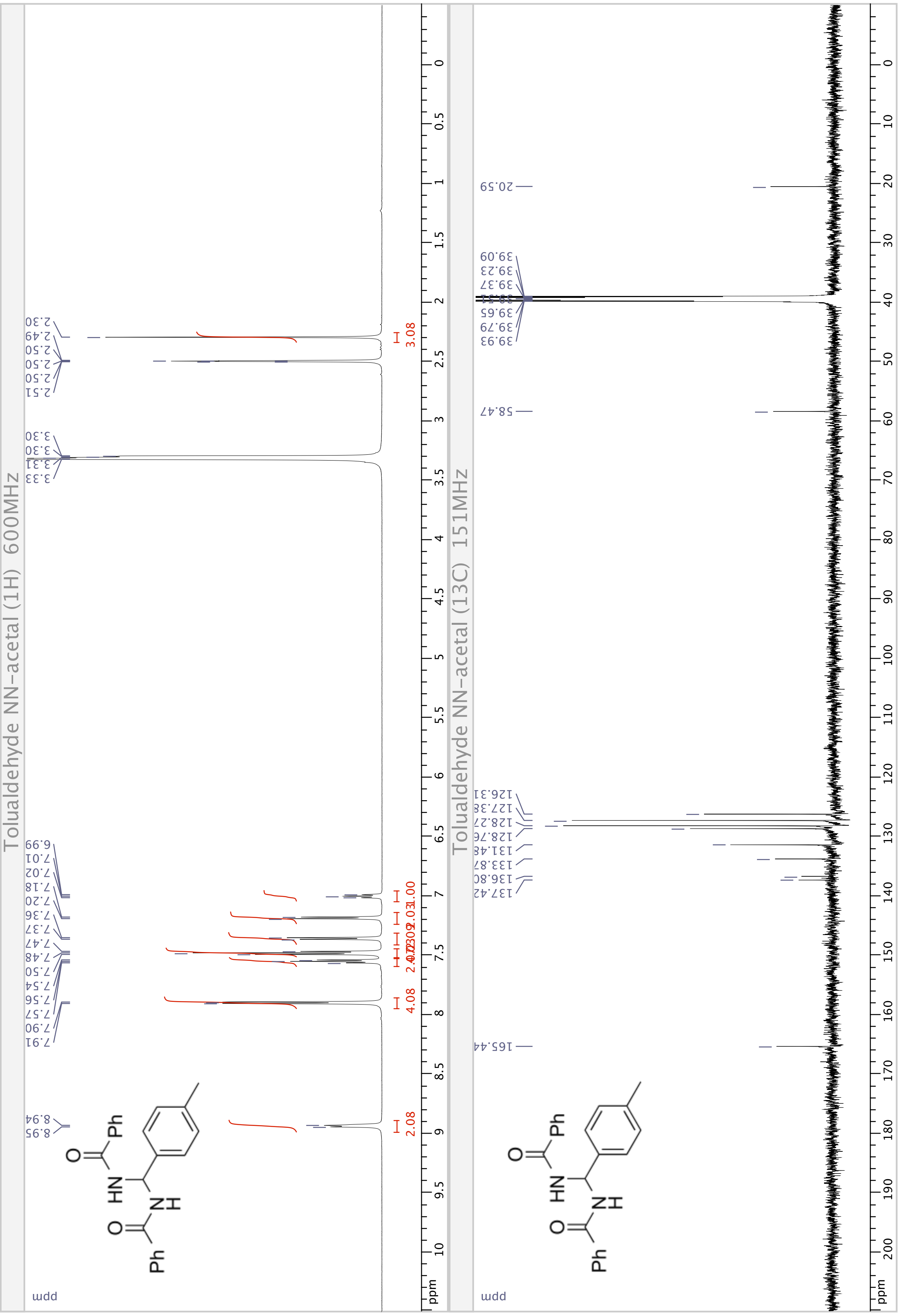
Tolualdehyde NN-acetal (1H) 600MHz
Tolualdehyde NN-acetal (13C) 151MHz

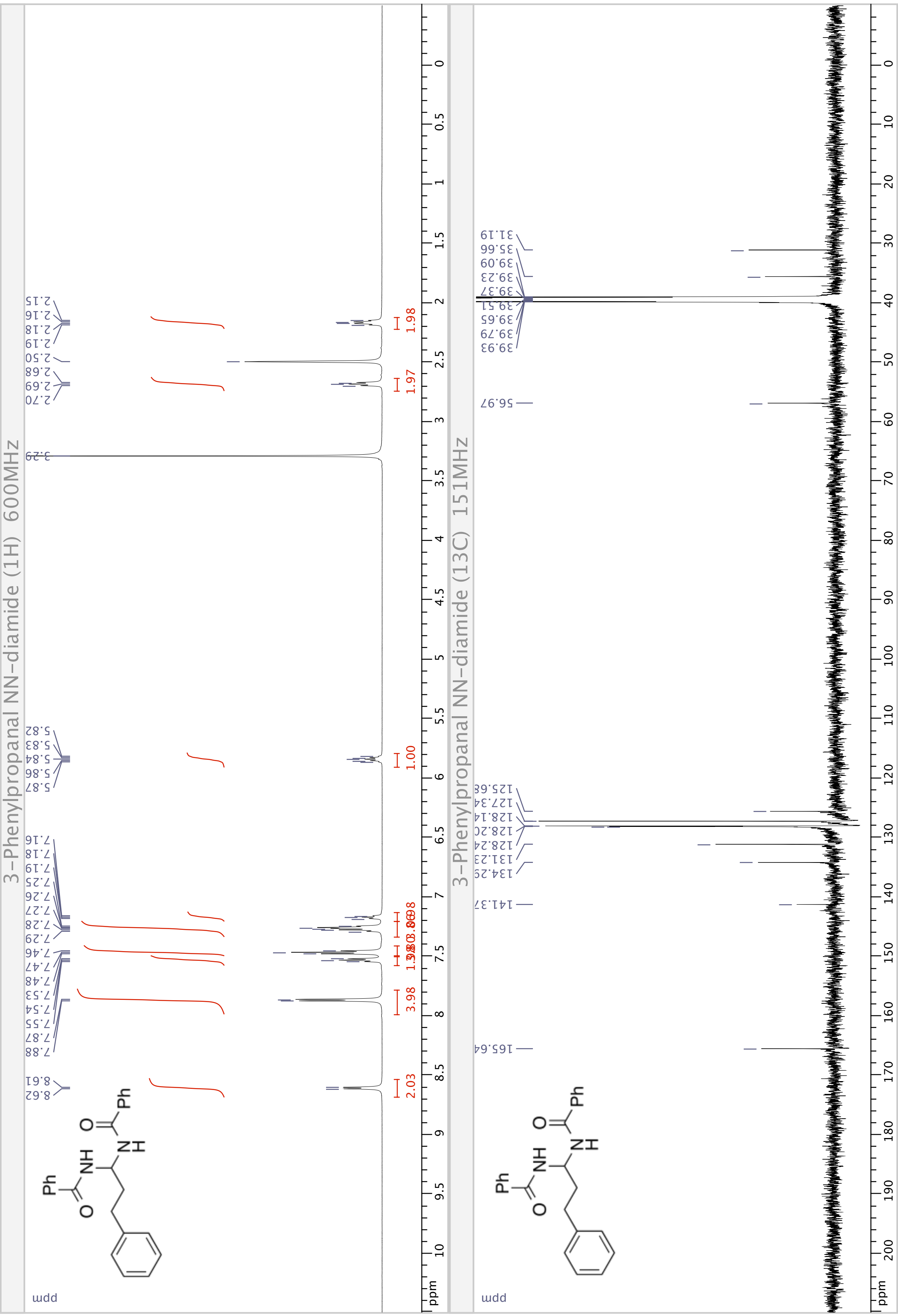
3-Phenylpropanal NN-diamide (1H) 600MHz
2.15
2.16
2.18
2.19
2.50
2.68
2.69
2.70
3.29
5.82
5.83
5.84
5.86
5.87
7.16
7.18
7.19
7.25
7.26
7.27
7.28
7.29
7.46
7.47
7.48
7.53
7.54
7.55
7.87
7.88
8.61
8.62
1.98
1.97
1.00
3.98
2.03
ppm
3-Phenylpropanal NN-diamide (13C) 151MHz
31.19
35.66
39.09
39.23
39.37
39.51
39.65
39.79
39.93
56.97
125.68
127.34
128.14
128.20
128.24
131.23
134.25
141.37
165.64
ppm

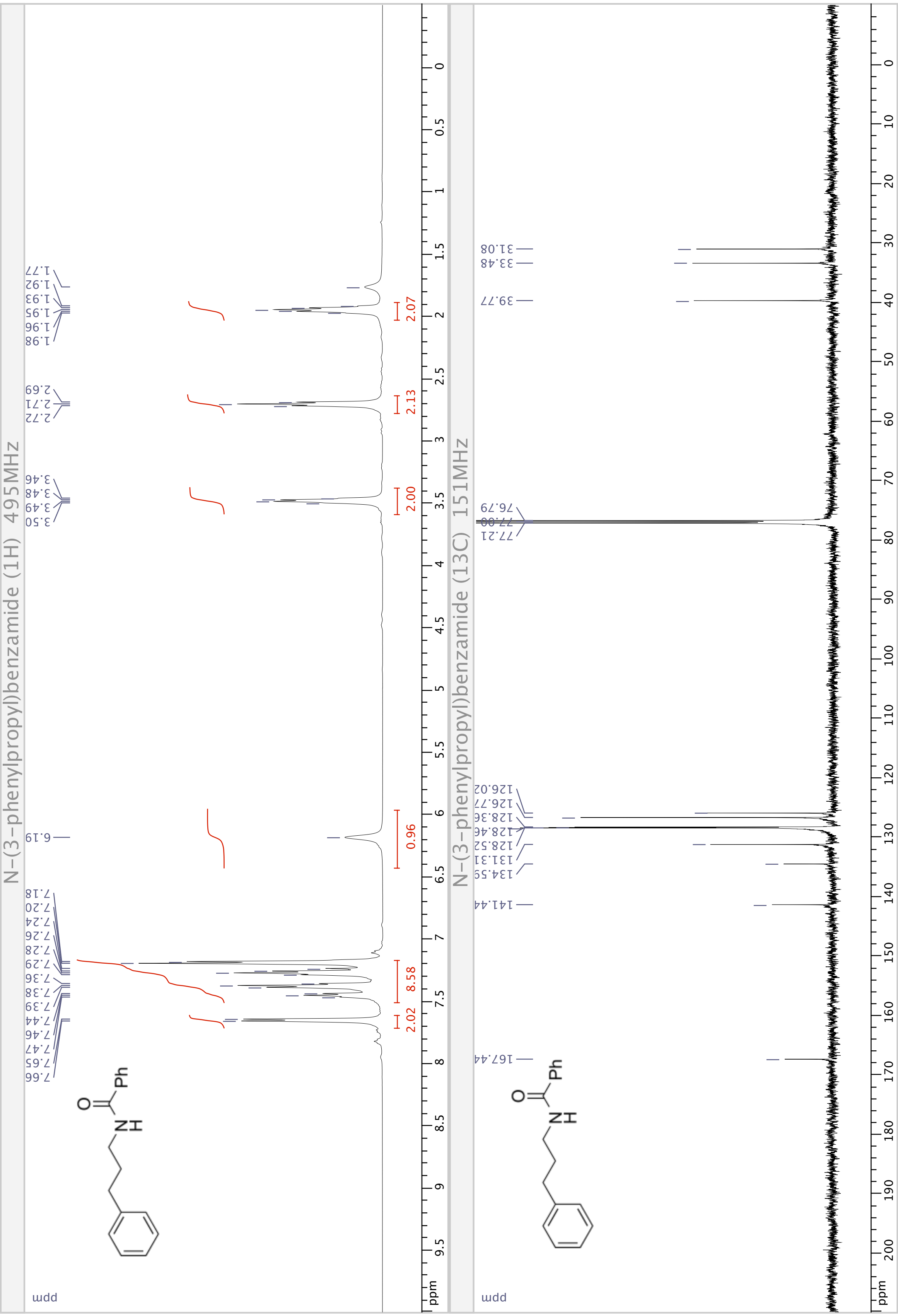
N-(3-phenylpropyl)benzamide (1H) 495MHz
1.77 1.92 1.93 1.95 1.96 1.98
2.69 2.71 2.72
3.46 3.48 3.49 3.50
6.19
7.18 7.20 7.24 7.26 7.28 7.29 7.36 7.38 7.39 7.44 7.46 7.47 7.65 7.66
2.07 2.13 2.00 0.96 8.58 2.02
N-(3-phenylpropyl)benzamide (13C) 151MHz
31.08 33.48 39.77
76.79 77.00 77.21
126.02 126.77 128.36 128.46 128.52 131.31 134.59 141.44 167.44
ppm

6 References

1 H. C. Brown, E. J. Mead and B. C. Subba Rao, *J. Am. Chem. Soc.*, 1955, **77**, 6209-6213.

2 H. Miyamura, R. Matsubara, Y. Miyazaki and S. Kobayashi, *Angew. Chem. Int. Ed.*, 2007, **46**, 4151-4154.

3 J.-F. Soulé, H. Miyamura and S. Kobayashi, *J. Am. Chem. Soc.*, 2011, **133**, 18550-18553.

4 J.-F. Soulé, H. Miyamura and S. Kobayashi, *Chem. Asian J.*, 2013, **8**, 2614-2626.

5 R. Yamashita, A. Sakakura and K. Ishihara, *Org. Lett.*, 2013, **15**, 3654-3657.

6 C. Mamat, A. Flemming, M. Köckerling, J. Steinbach and F. R. Wuest, *Synthesis*, 2009, **2009**, 3311-3321.

7 X. Cui, Y. Zhang, F. Shi and Y. Deng, *Chem. Eur. J.*, 2011, **17**, 1021-1028.

8 L. U. Nordstrøm, H. Vogt and R. Madsen, *J. Am. Chem. Soc.*, 2008, **130**, 17672-17673.

9 T. K. Houlding, K. Tchabanenko, M. T. Rahman and E. V. Rebrov, *Org. Biomol. Chem.*, 2013, **11**, 4171-4177.

10 A. Martínez-Asencio, M. Yus and D. J. Ramón, *Synthesis*, 2011, **2011**, 3730-3740.

11 G. Harichandran, S. D. Amalraj and P. Shanmugam, *Indian J. Chem., Sect. B: Org. Chem. Incl. Med. Chem.*, 2011, **50B**, 77-82.

12 W. A. Nack, G. He, S.-Y. Zhang, C. Lu and G. Chen, *Org. Lett.*, 2013, **15**, 3440-3443.
